# Supplementary material for: All-trans retinoic acid downregulates HBx levels via E6-associated protein-mediated proteasomal degradation to suppress hepatitis B virus replication
Source: PLoS One. 2024 Jun 11;19(6):e0305350. doi: 10.1371/journal.pone.0305350 (PMC11166335; doi:10.1371/journal.pone.0305350)

Fig. 1a

Lanes	1	2	3	4
HBV (MOI)	0	50	50	50
ATRA (μM)	0	0	2	5

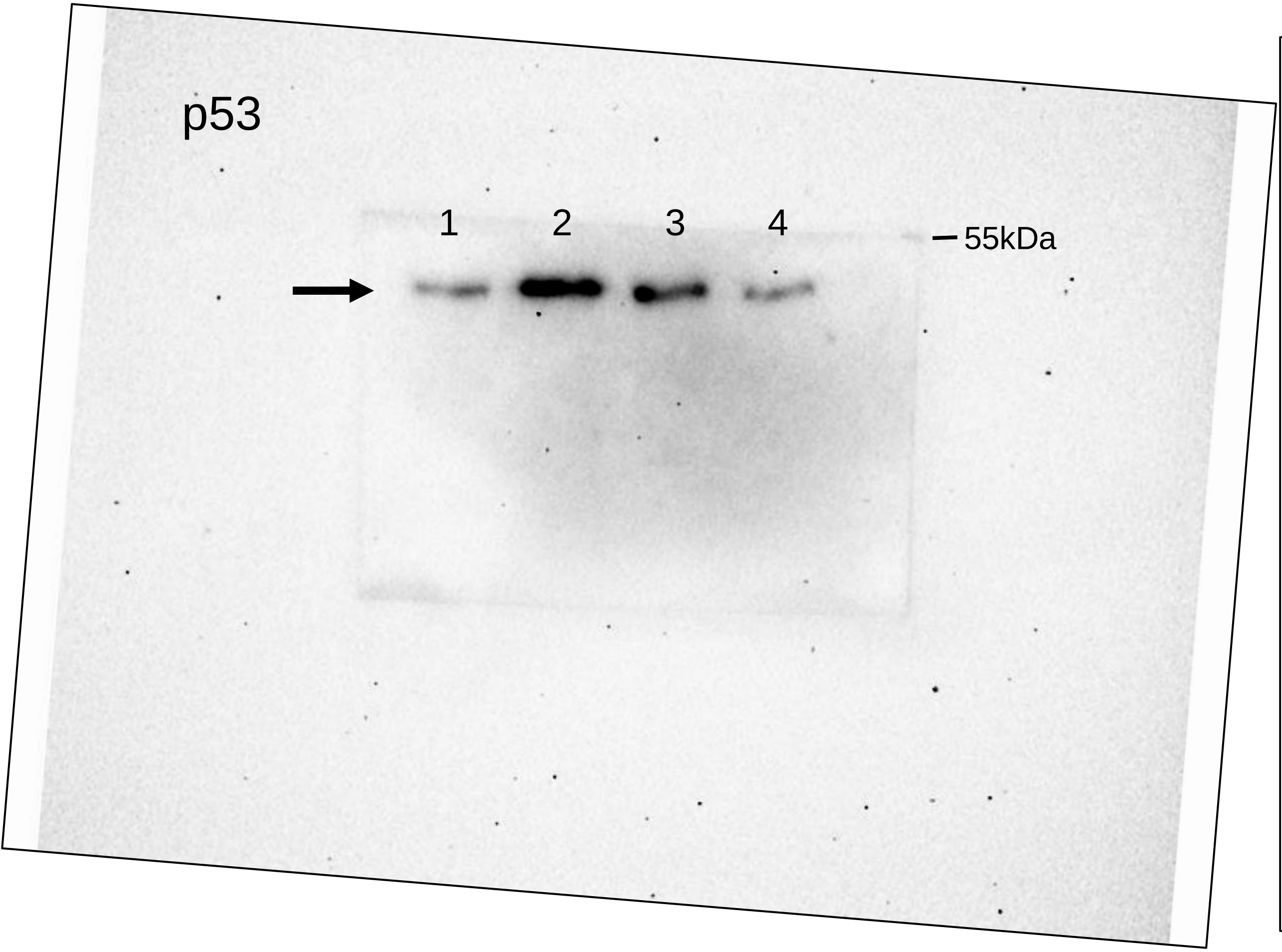

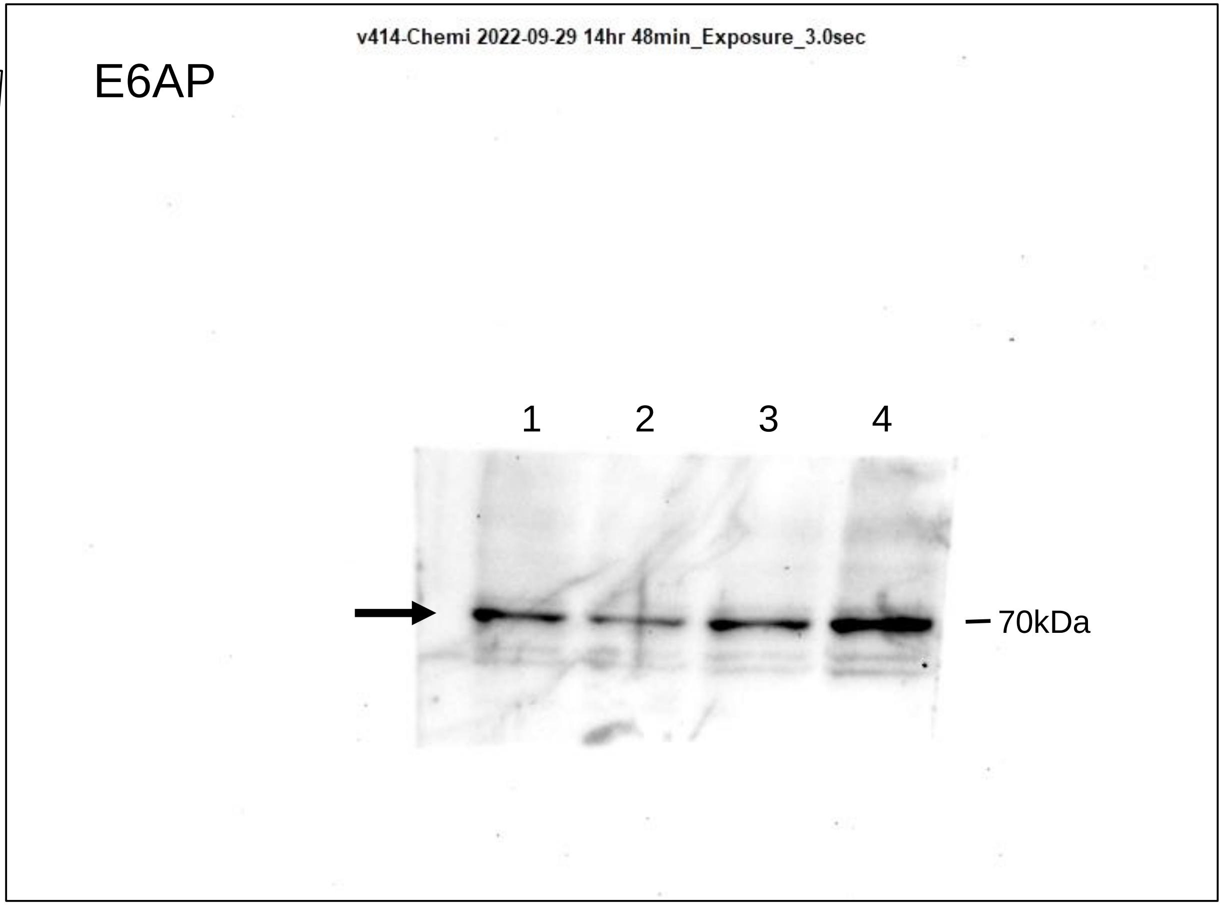

Fig. 1a

Lanes	1	2	3	4
HBV (MOI)	0	50	50	50
ATRA (μM)	0	0	2	5

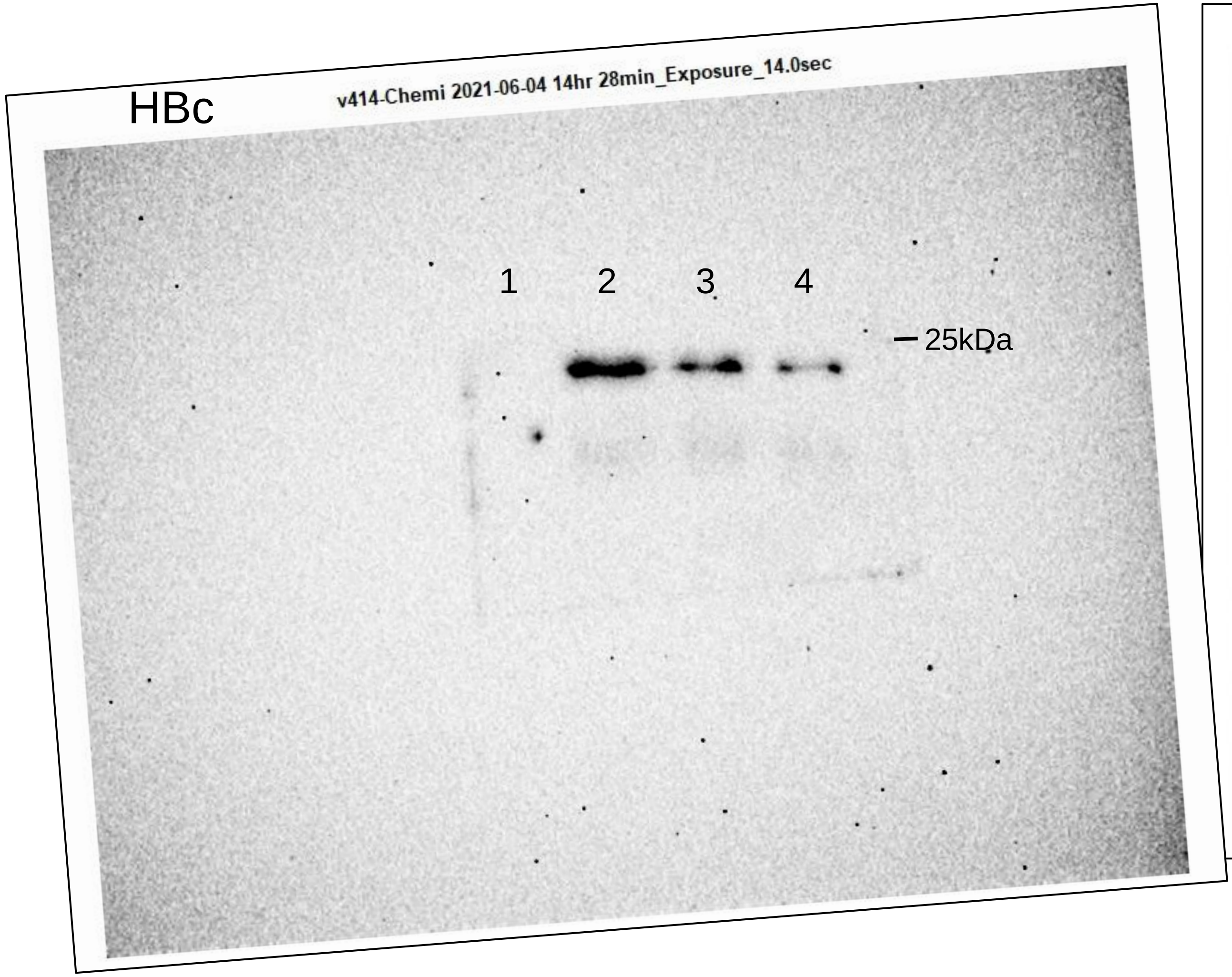

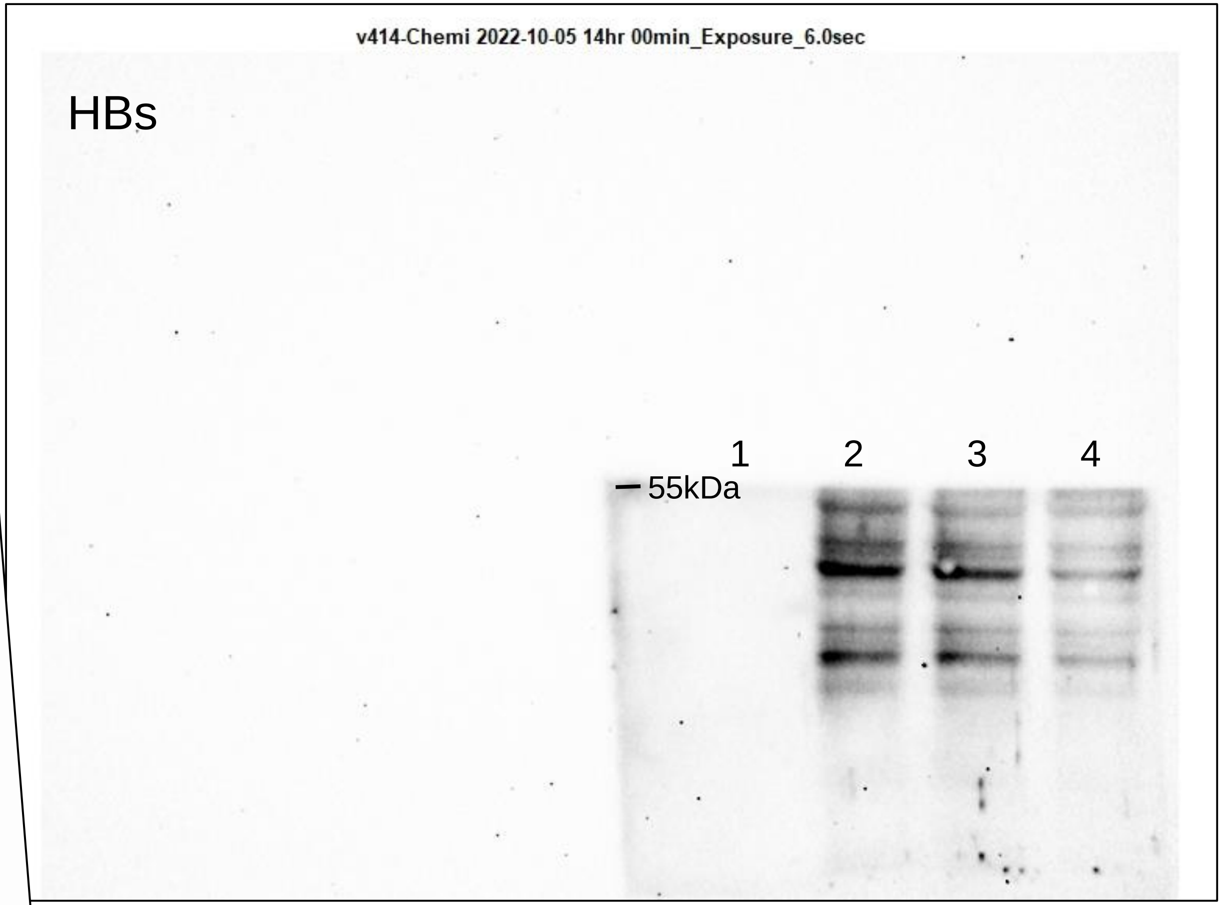

Fig. 1d

Lanes	1	2	3	4
HBV (MOI)	0	50	50	50
ATRA (μM)	0	0	2	5

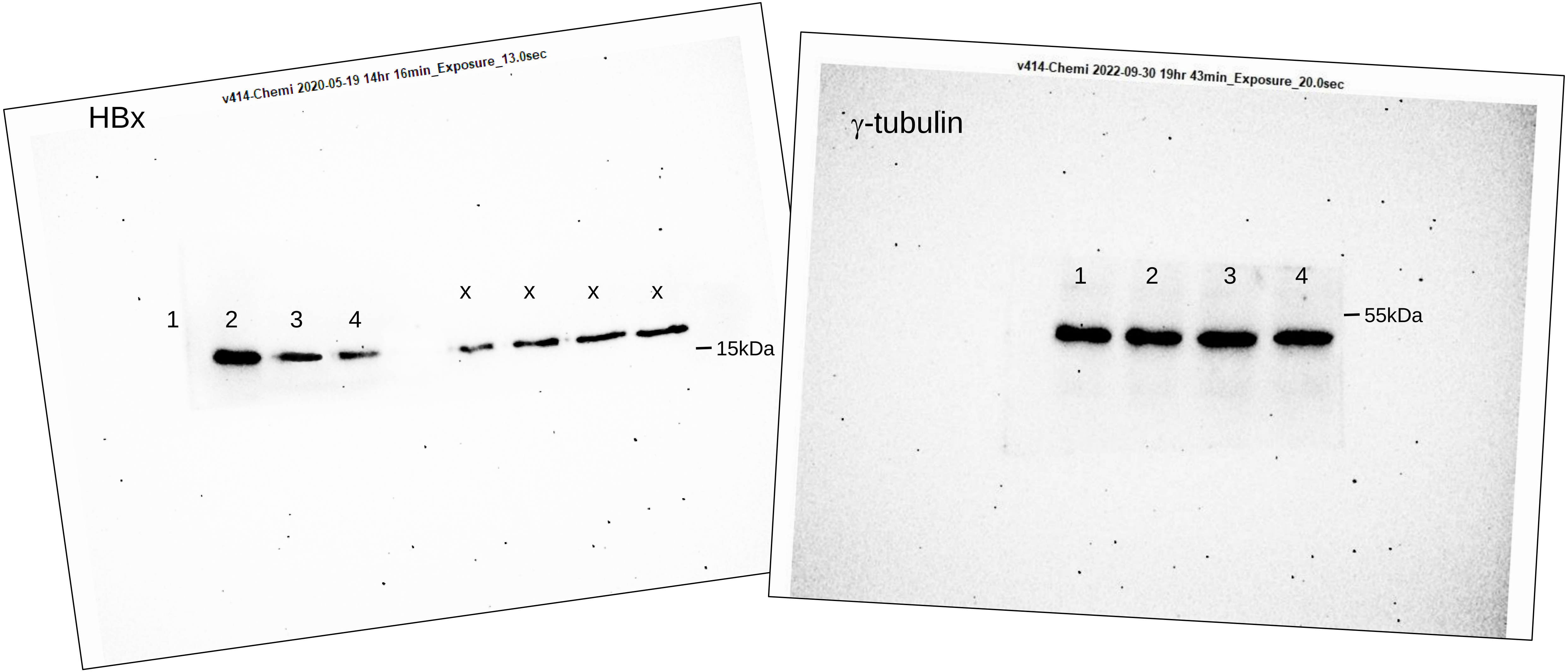

Fig. 1d

Lanes	1	2	3	4
HBV (MOI)	0	50	50	50
ATRA (μM)	0	0	2	5

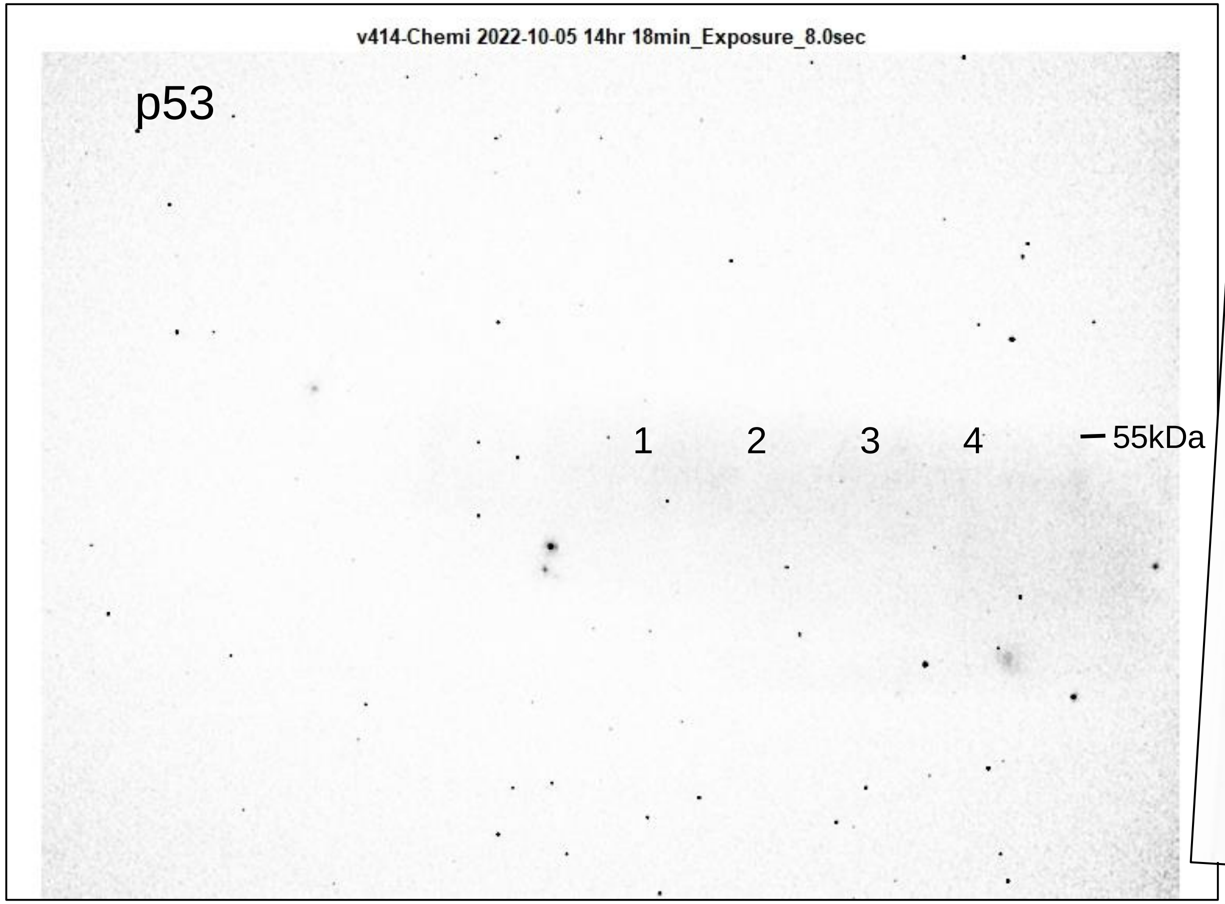

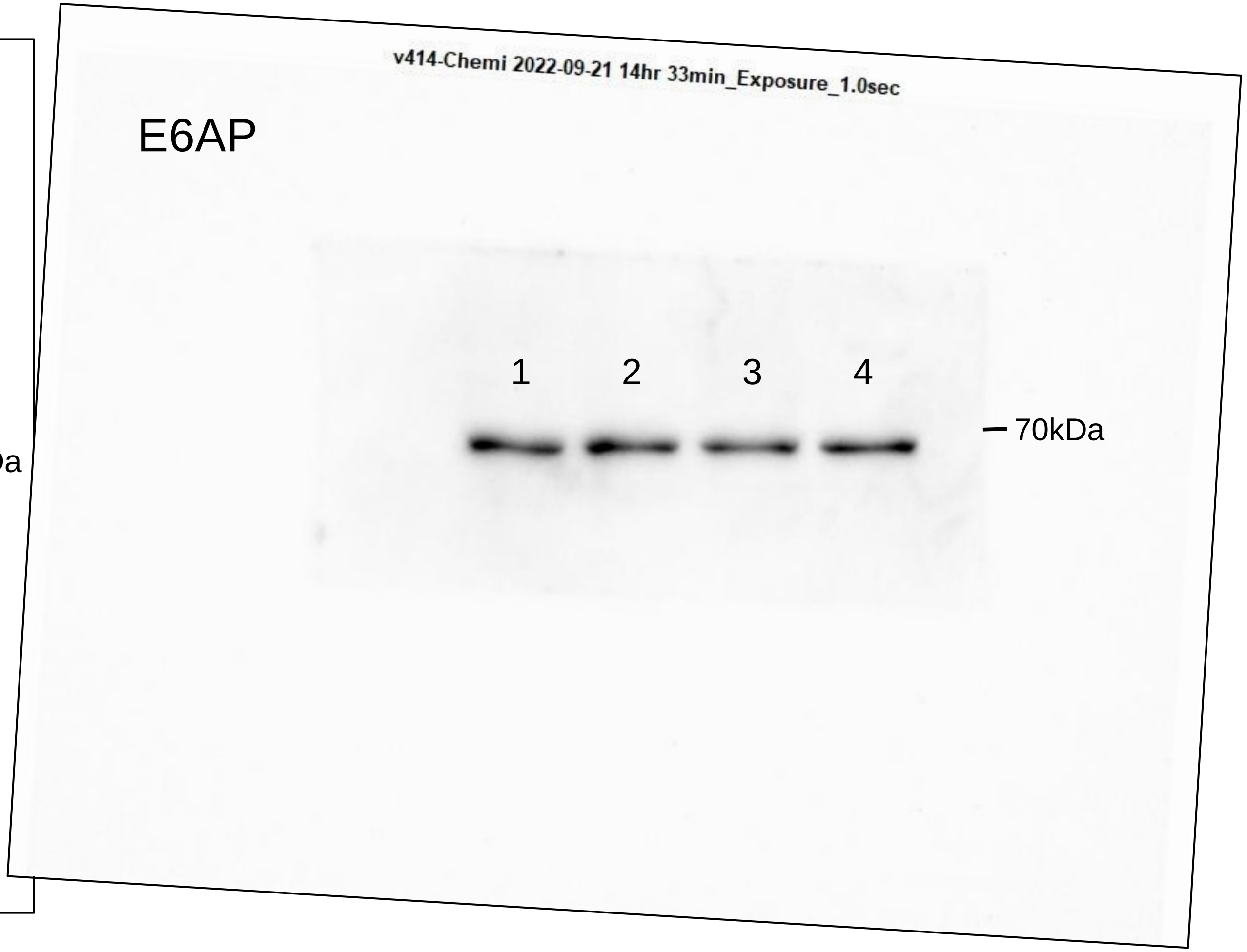

Fig. 1d

Lanes	1	2	3	4
HBV (MOI)	0	50	50	50
ATRA (μM)	0	0	2	5

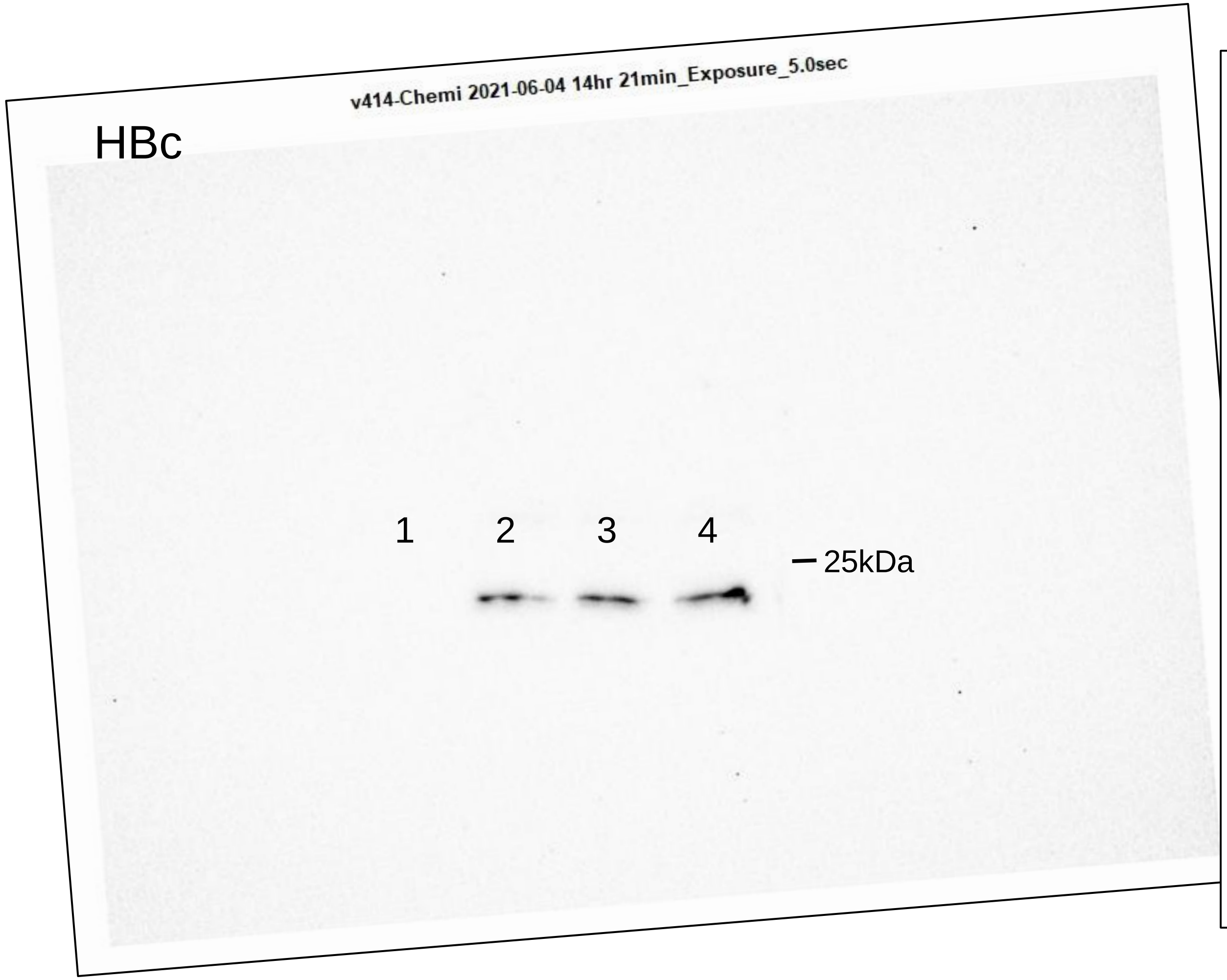

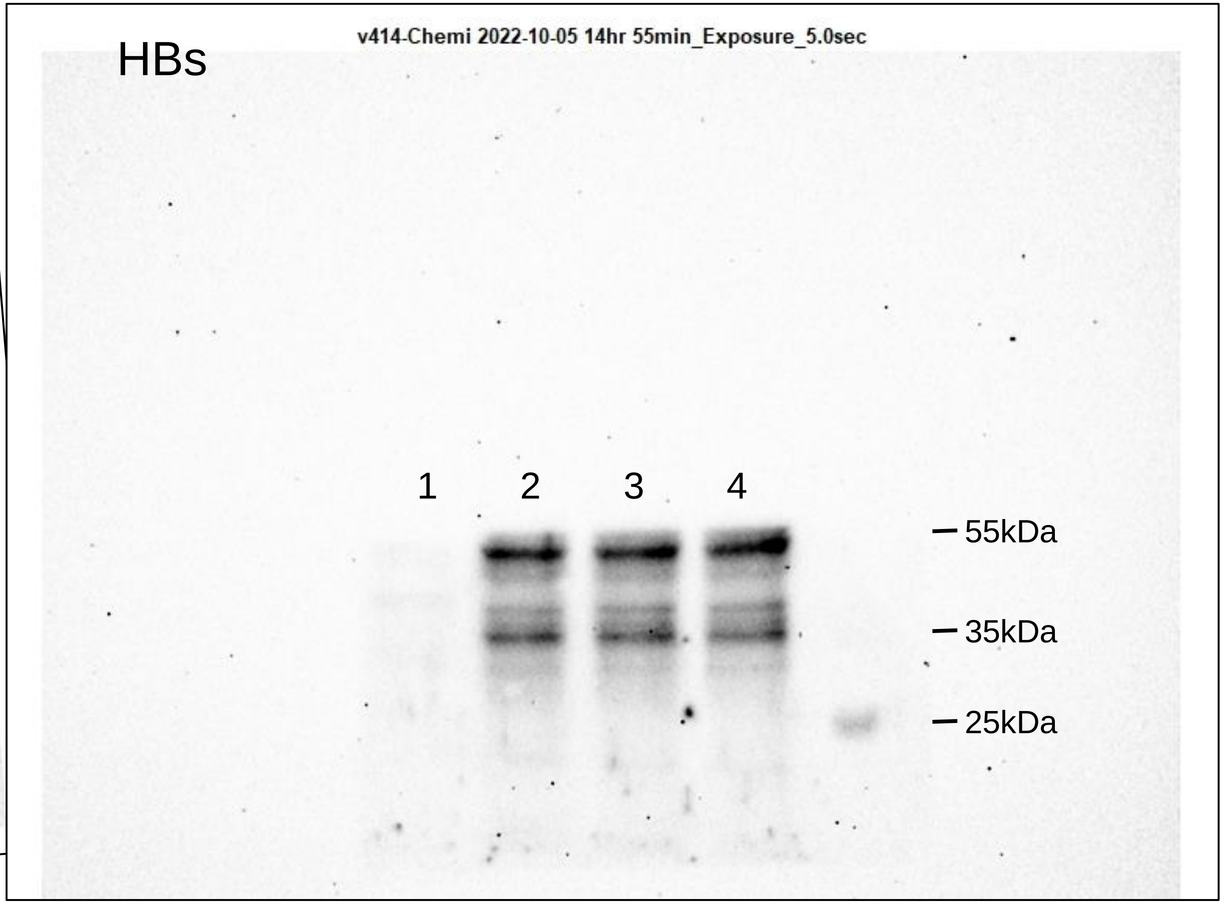

Fig. 1d

Lanes	1	2	3	4
HBV (MOI)	0	50	50	50
ATRA (μM)	0	0	2	5

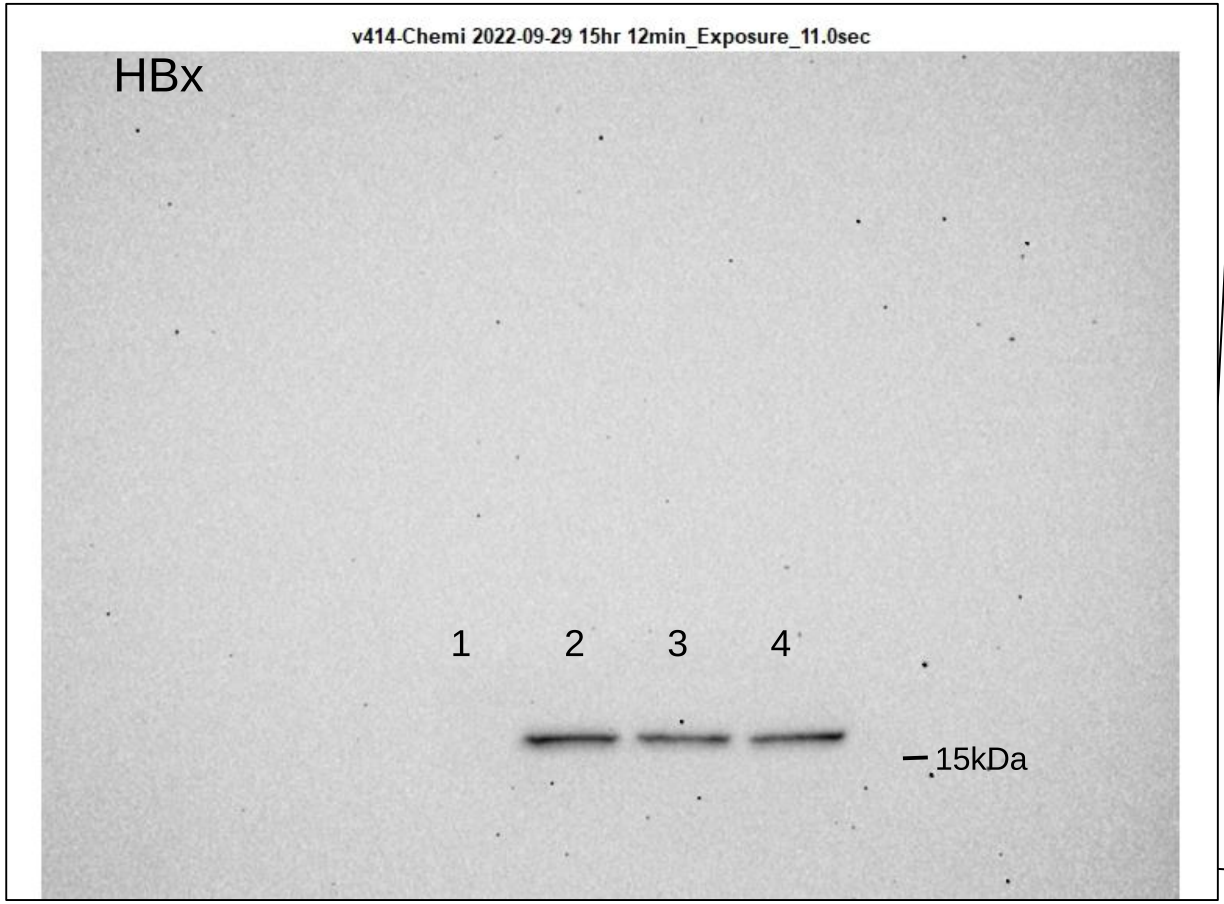

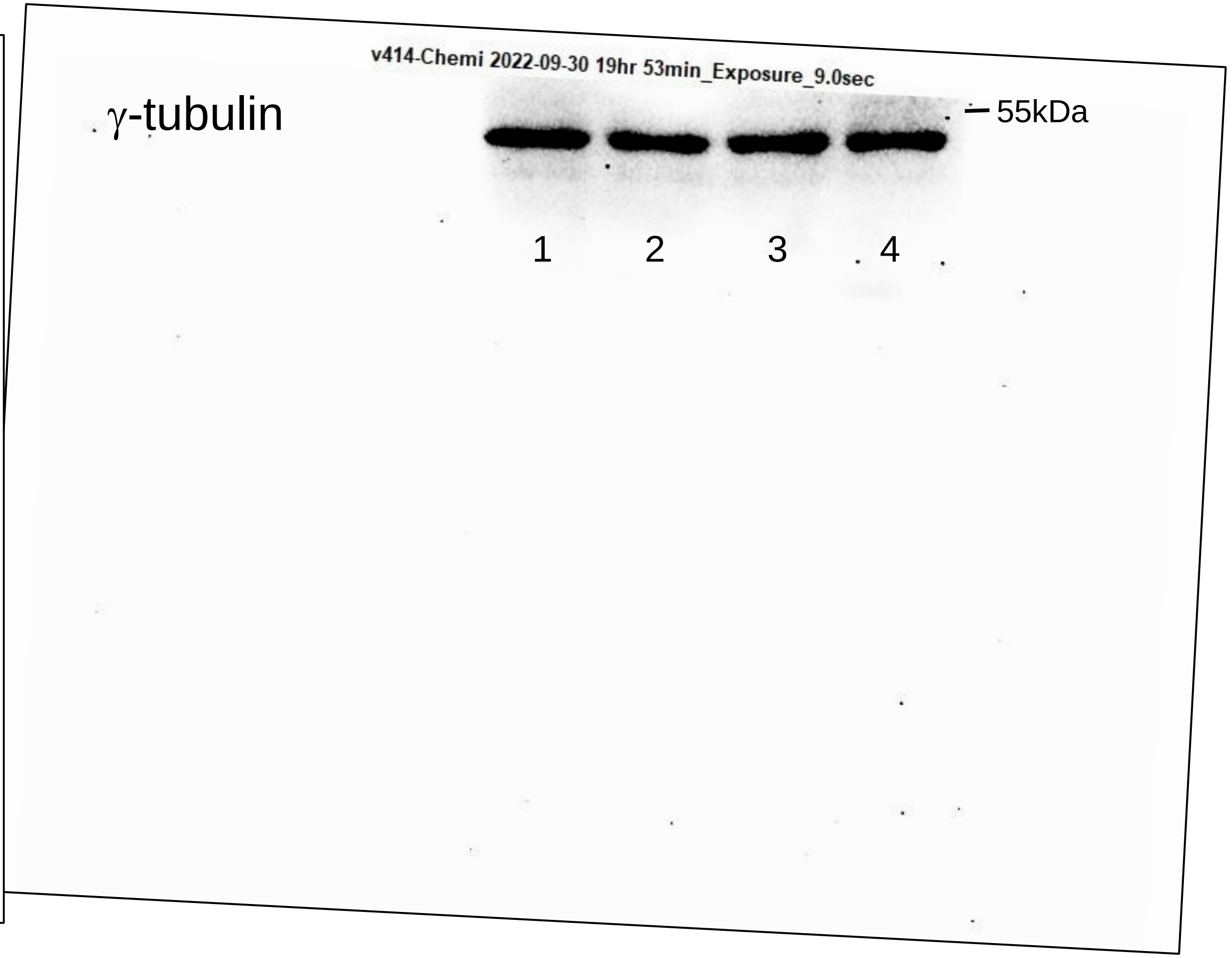

Fig. 2a

Lanes	1	2	3	4	5	6	7	8
HBx-null HBV (MOI)	0	50	50	50	0	50	50	50
ATRA (μM)	0	0	2	5	0	0	2	5

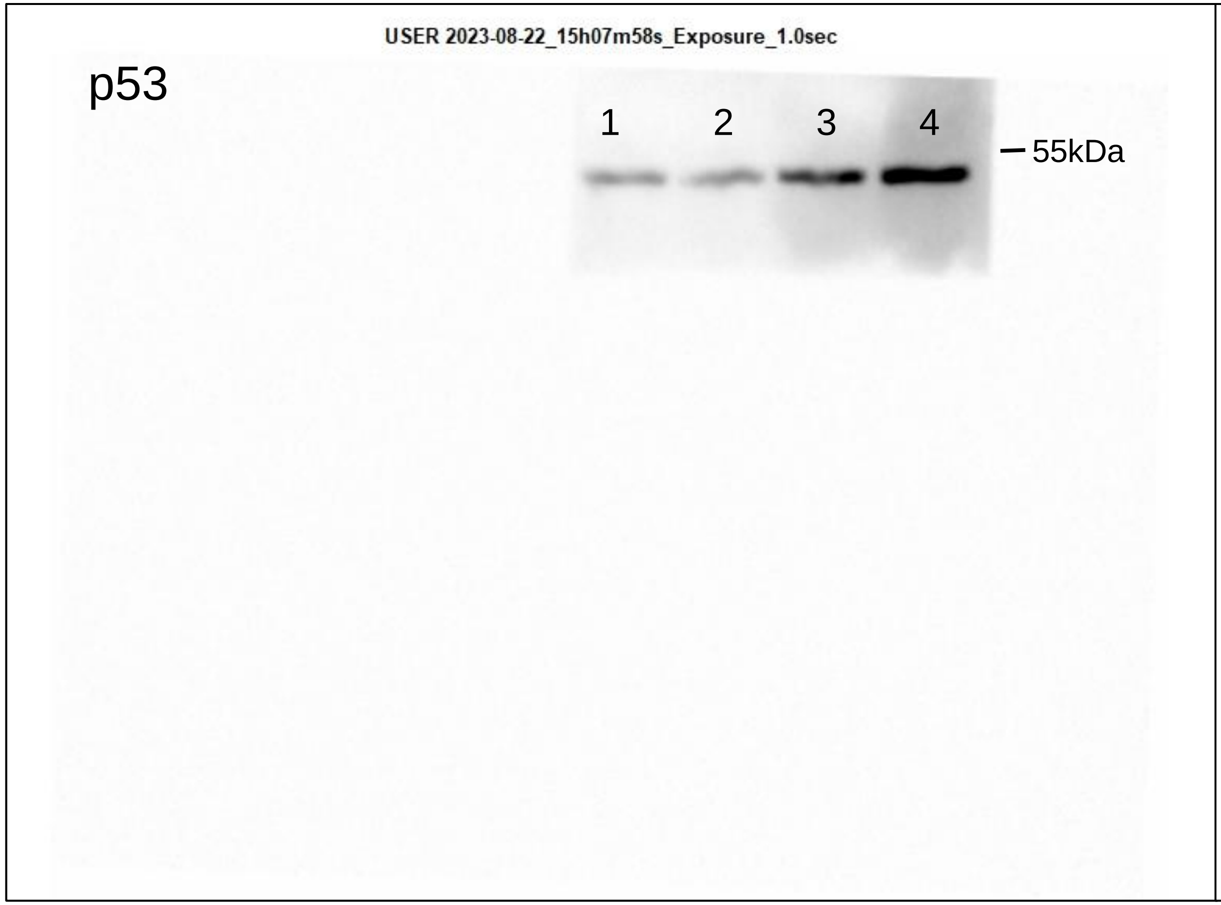

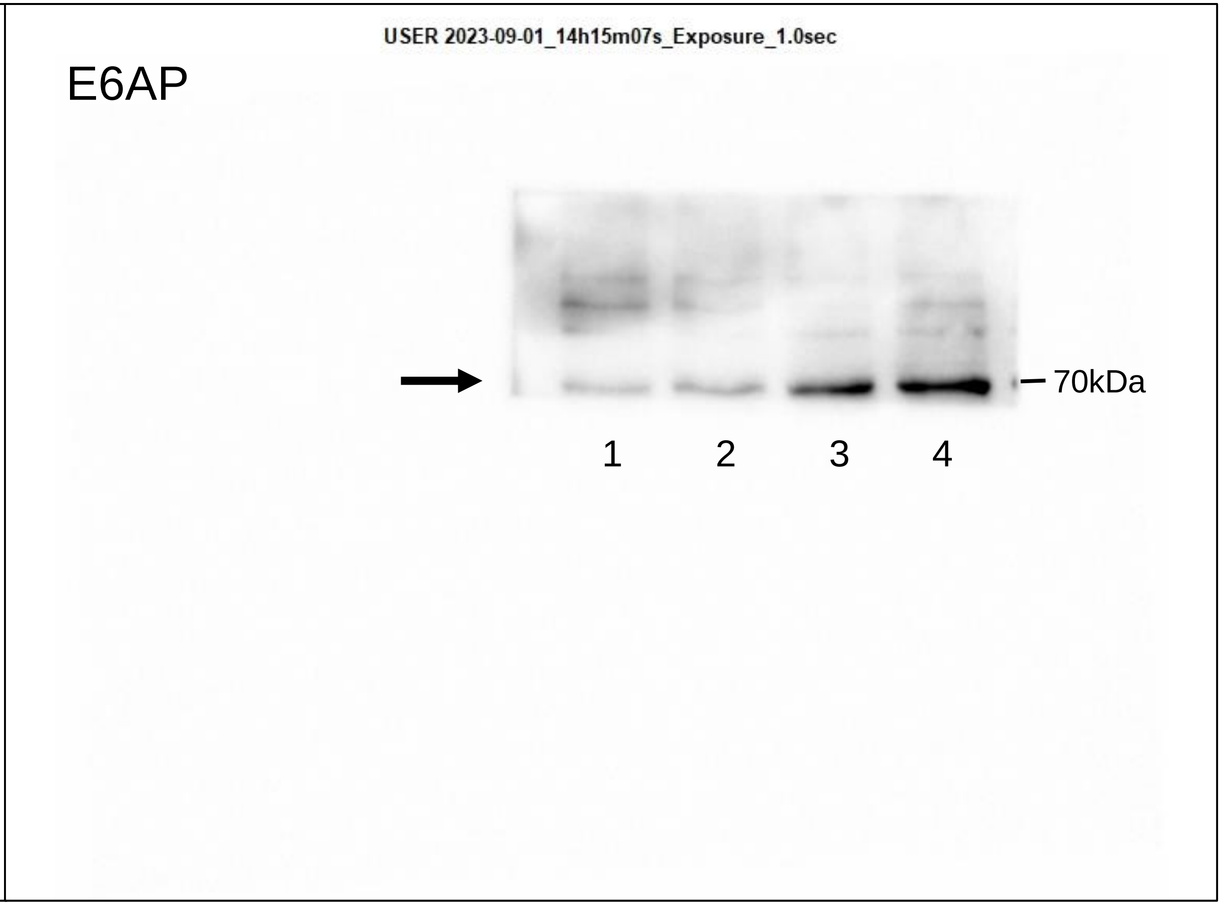

Fig. 2a

Lanes	1	2	3	4	5	6	7	8
HBx-null HBV (MOI)	0	50	50	50	0	50	50	50
ATRA (μM)	0	0	2	5	0	0	2	5

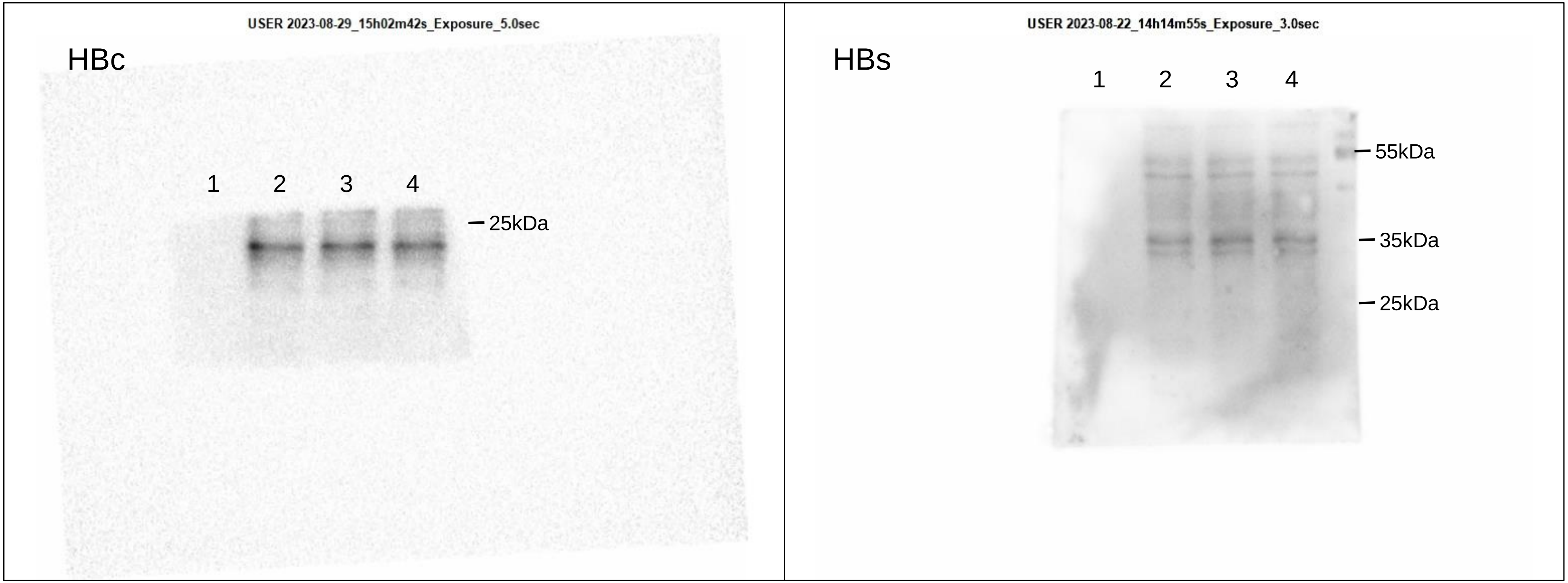

Fig. 2a

Lanes	1	2	3	4	5	6	7	8
HBx-null HBV (MOI)	0	50	50	50	0	50	50	50
ATRA (μM)	0	0	2	5	0	0	2	5

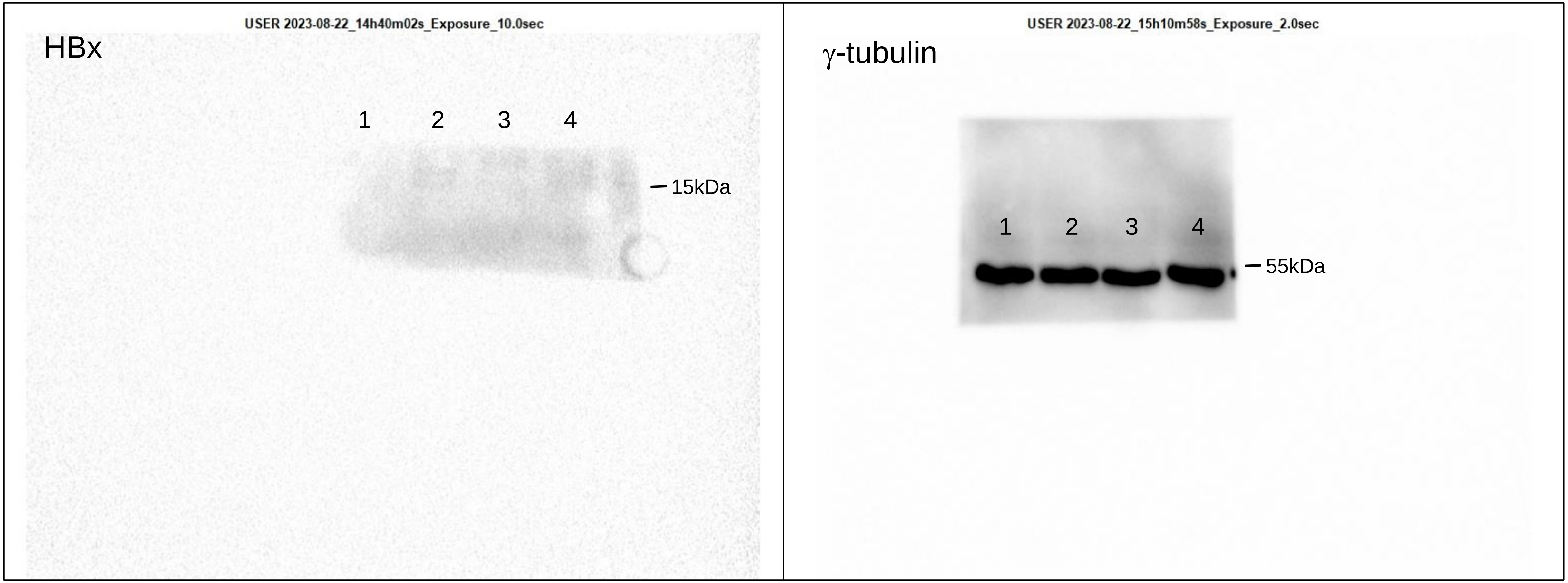

Fig. 2a

Lanes	1	2	3	4	5	6	7	8
HBx-null HBV (MOI)	0	50	50	50	0	50	50	50
ATRA (μM)	0	0	2	5	0	0	2	5

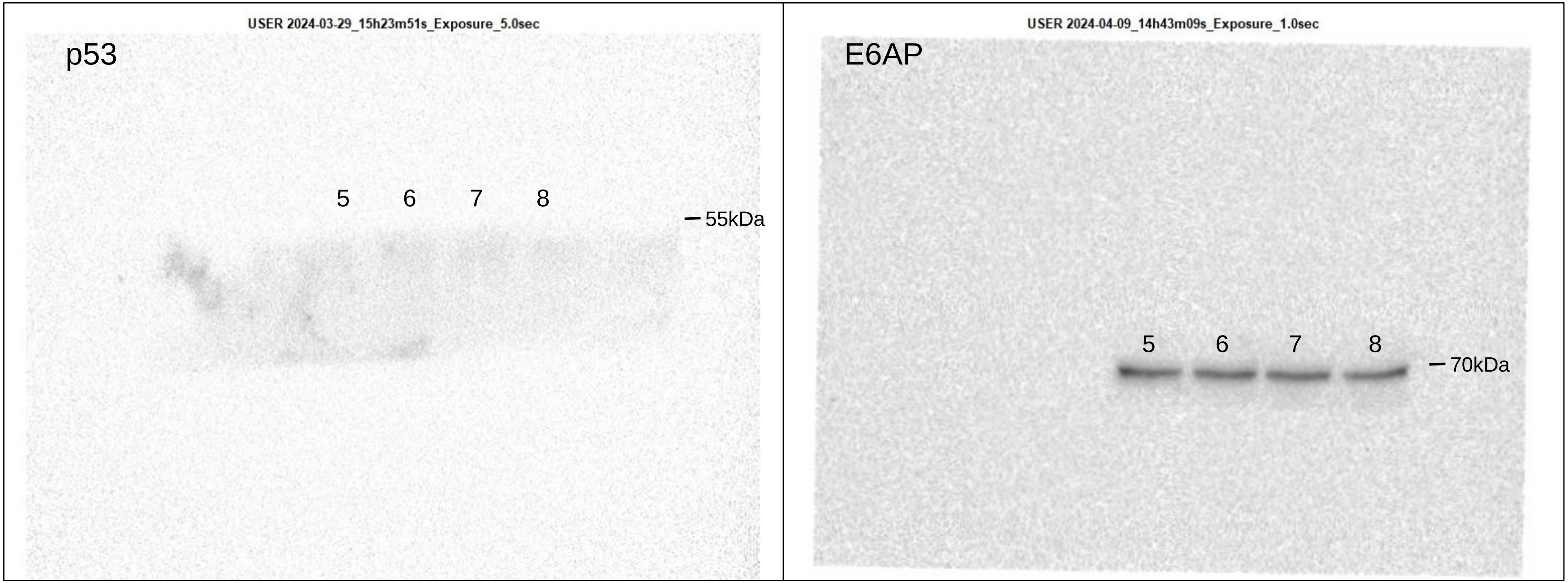

Fig. 2a

Lanes	1	2	3	4	5	6	7	8
HBx-null HBV (MOI)	0	50	50	50	0	50	50	50
ATRA (μM)	0	0	2	5	0	0	2	5

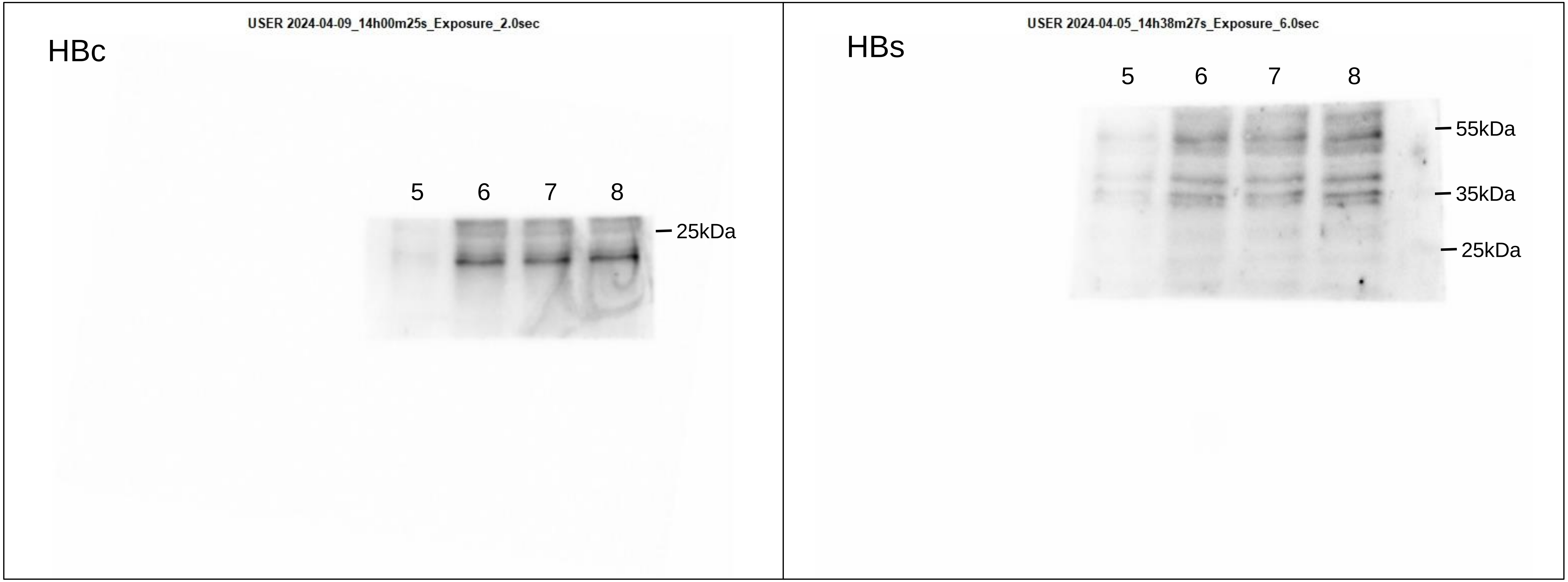

Fig. 2a

Lanes	1	2	3	4	5	6	7	8
HBx-null HBV (MOI)	0	50	50	50	0	50	50	50
ATRA (μM)	0	0	2	5	0	0	2	5

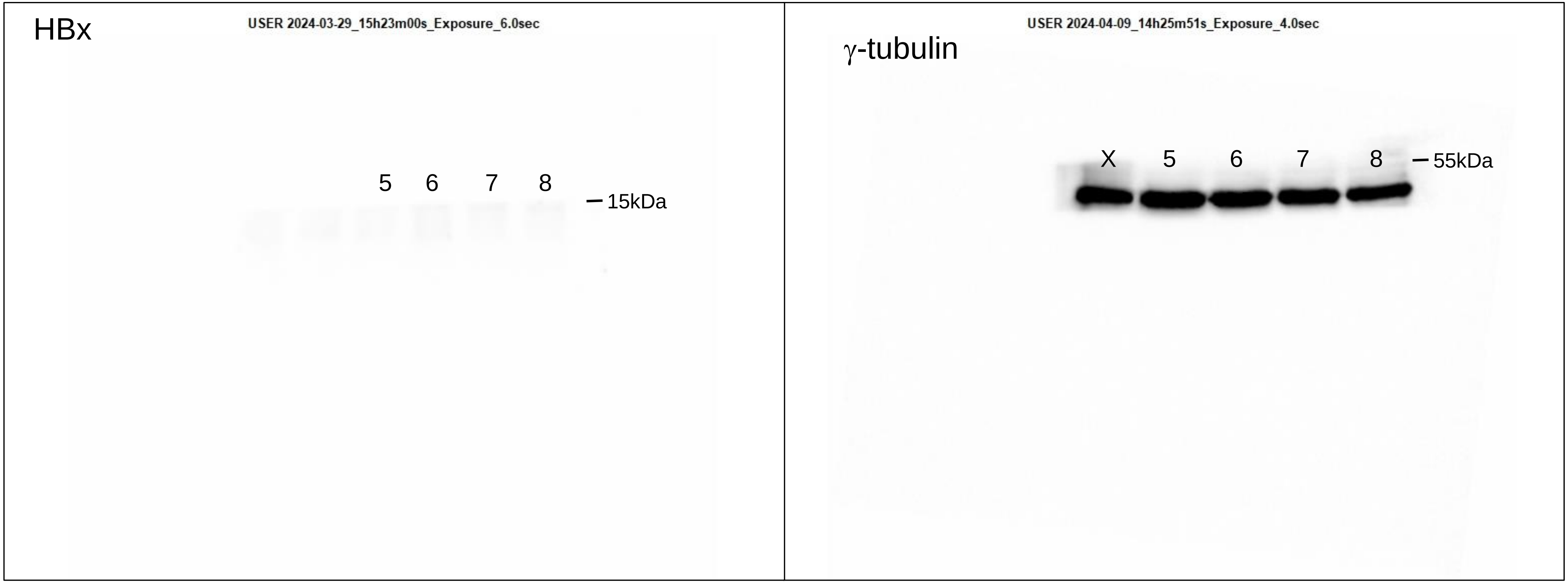

Fig. 2d

Lanes	1	2	3	4	5	6
ATRA (μM)	0	0.01	0.1	1.0	2.0	5.0

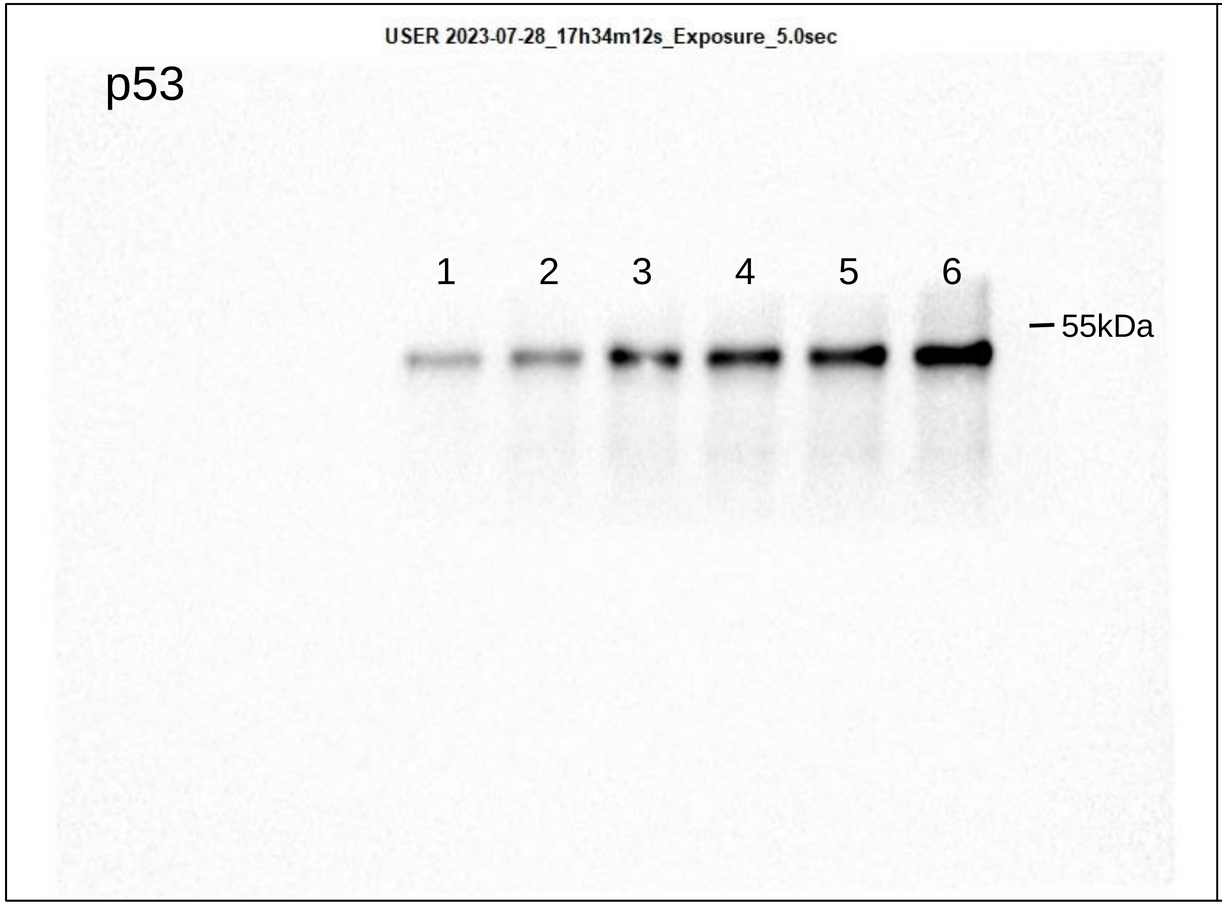

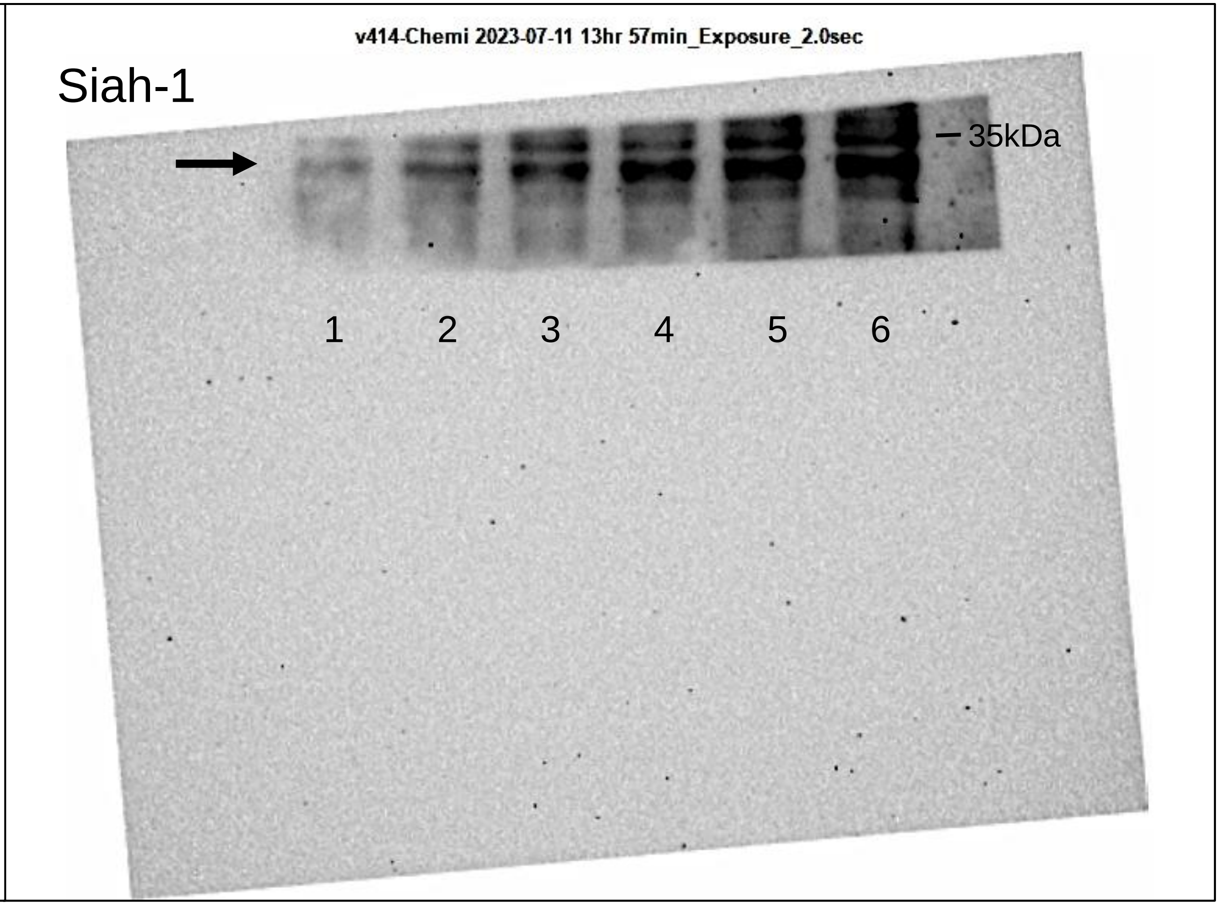

Fig. 2d

Lanes	1	2	3	4	5	6
ATRA (μM)	0	0.01	0.1	1.0	2.0	5.0

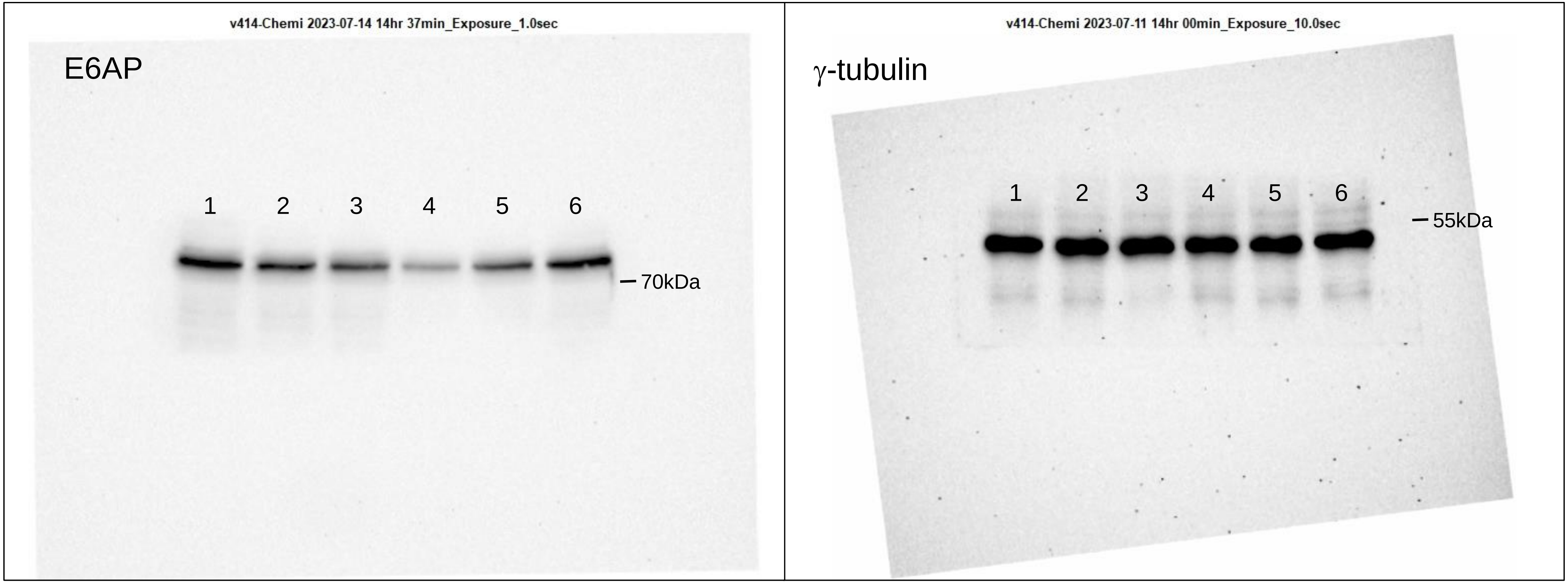

Fig. 2e

Lanes	1	2	3	4	5	6
HBx (μg)	0	0.5	0.5	0.5	0.5	0.5
ATRA (μM)	0	0	0.1	1.0	2.0	5.0

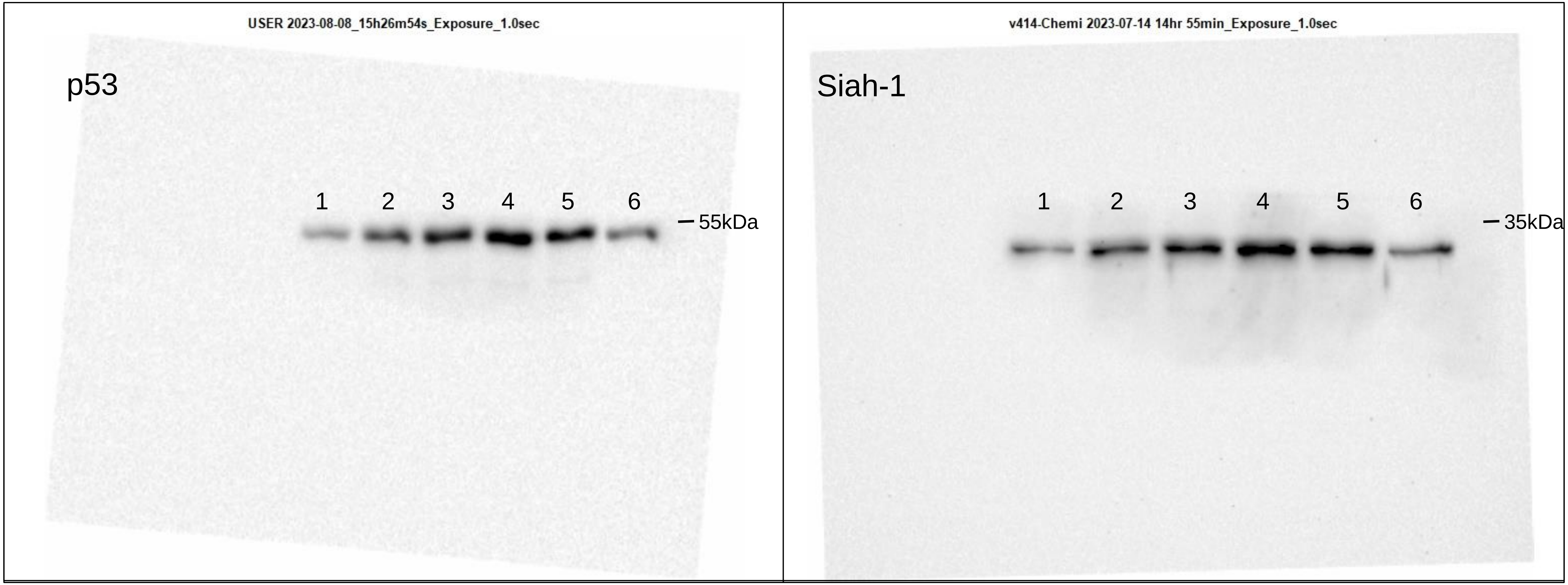

Fig. 2e

Lanes	1	2	3	4	5	6
HBx (μg)	0	0.5	0.5	0.5	0.5	0.5
ATRA (μM)	0	0	0.1	1.0	2.0	5.0

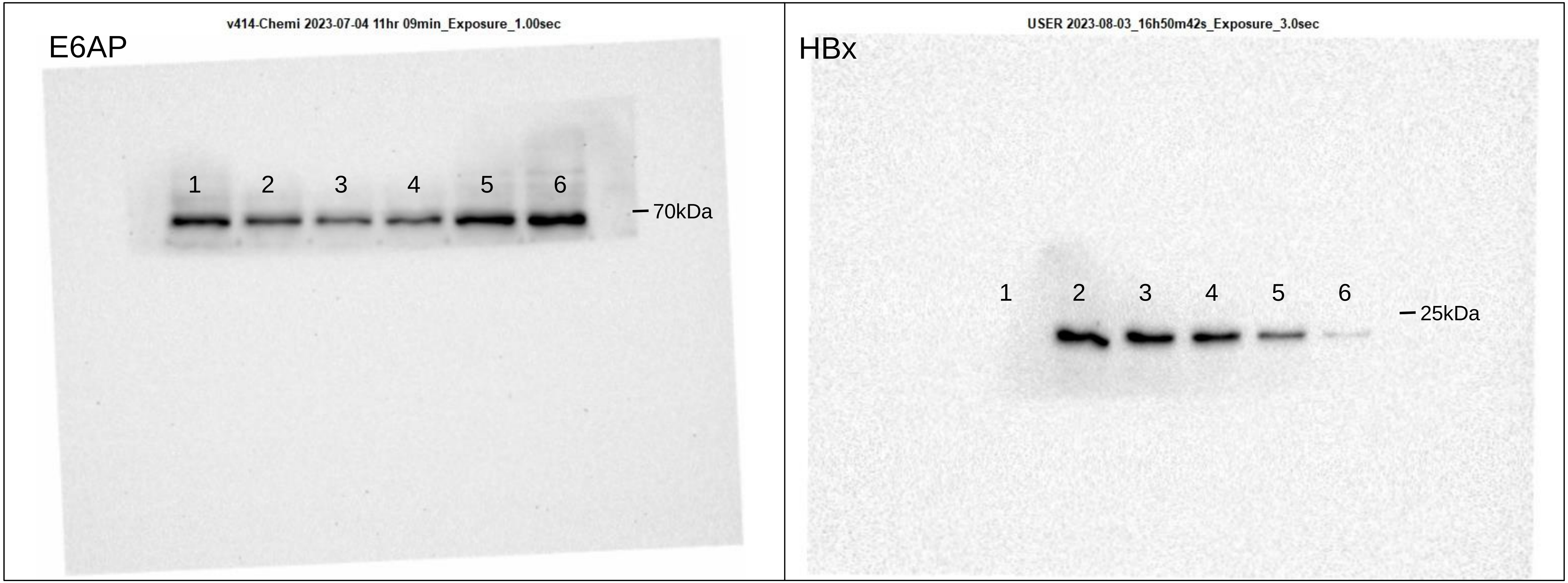

Fig. 2e

Lanes	1	2	3	4	5	6
HBx (μg)	0	0.5	0.5	0.5	0.5	0.5
ATRA (μM)	0	0	0.1	1.0	2.0	5.0

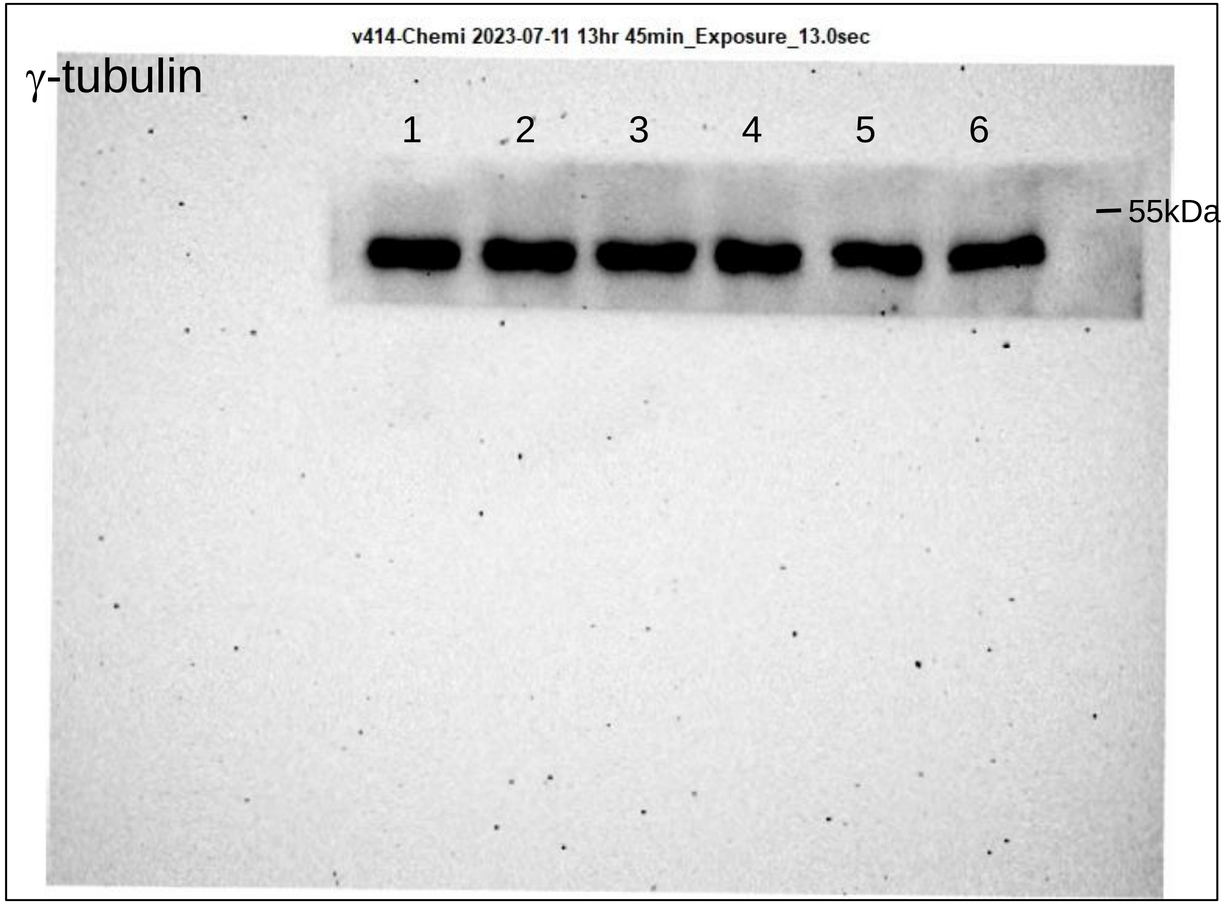

Fig. 2f

Lanes	1	2	3	4	5	6
HBx (μg)	0	0.5	0.5	0.5	0.5	0.5
ATRA (μM)	0	0	0.1	1.0	2.0	5.0

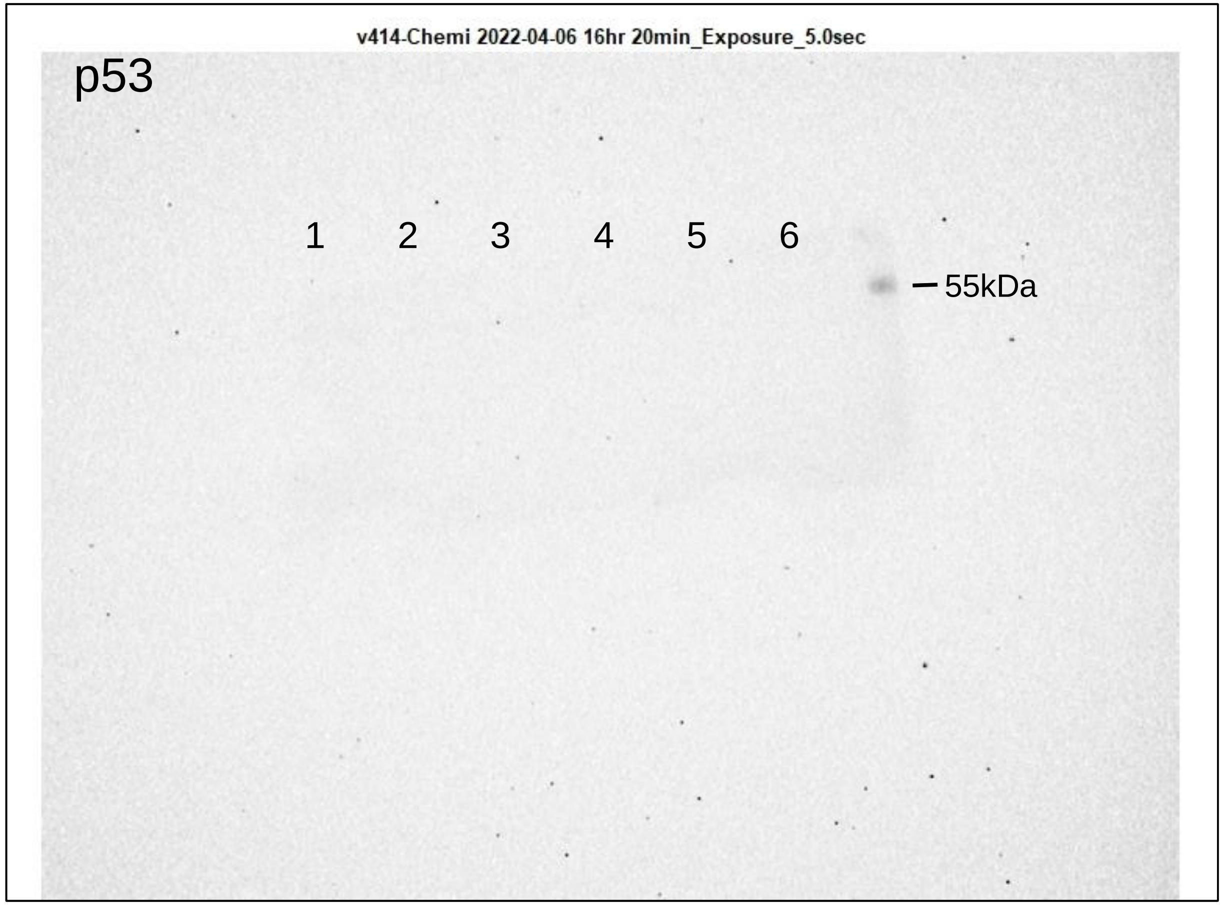

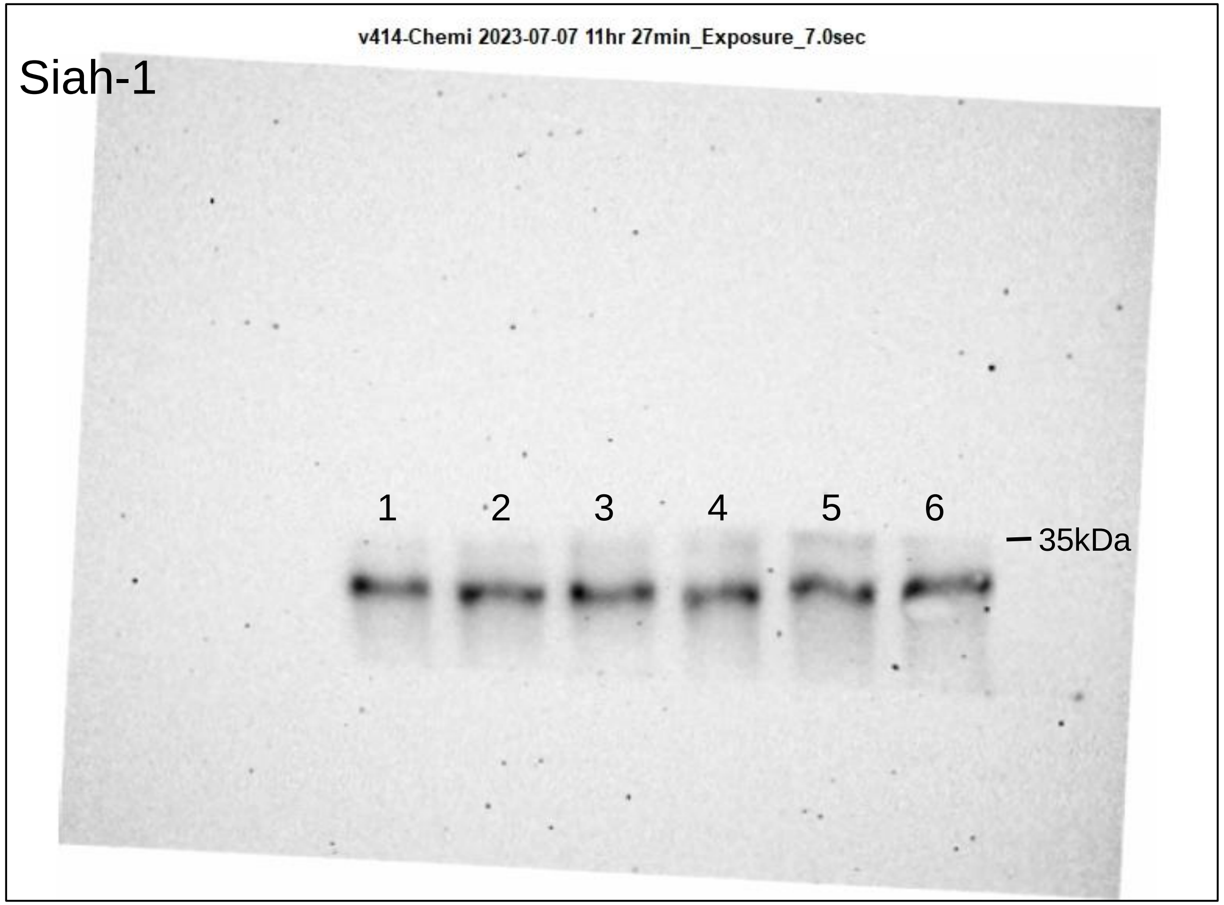

Fig. 2f

Lanes	1	2	3	4	5	6
HBx (μg)	0	0.5	0.5	0.5	0.5	0.5
ATRA (μM)	0	0	0.1	1.0	2.0	5.0

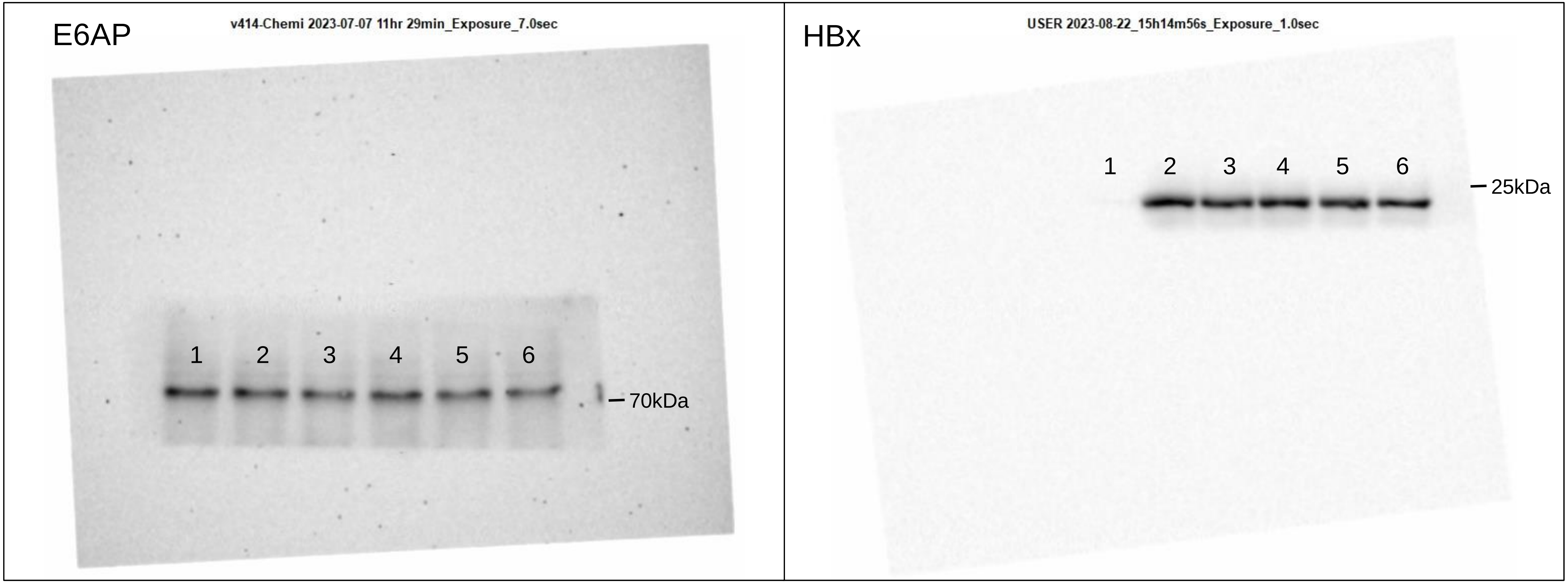

Fig. 2f

Lanes	1	2	3	4	5	6
HBx (μg)	0	0.5	0.5	0.5	0.5	0.5
ATRA (μM)	0	0	0.1	1.0	2.0	5.0

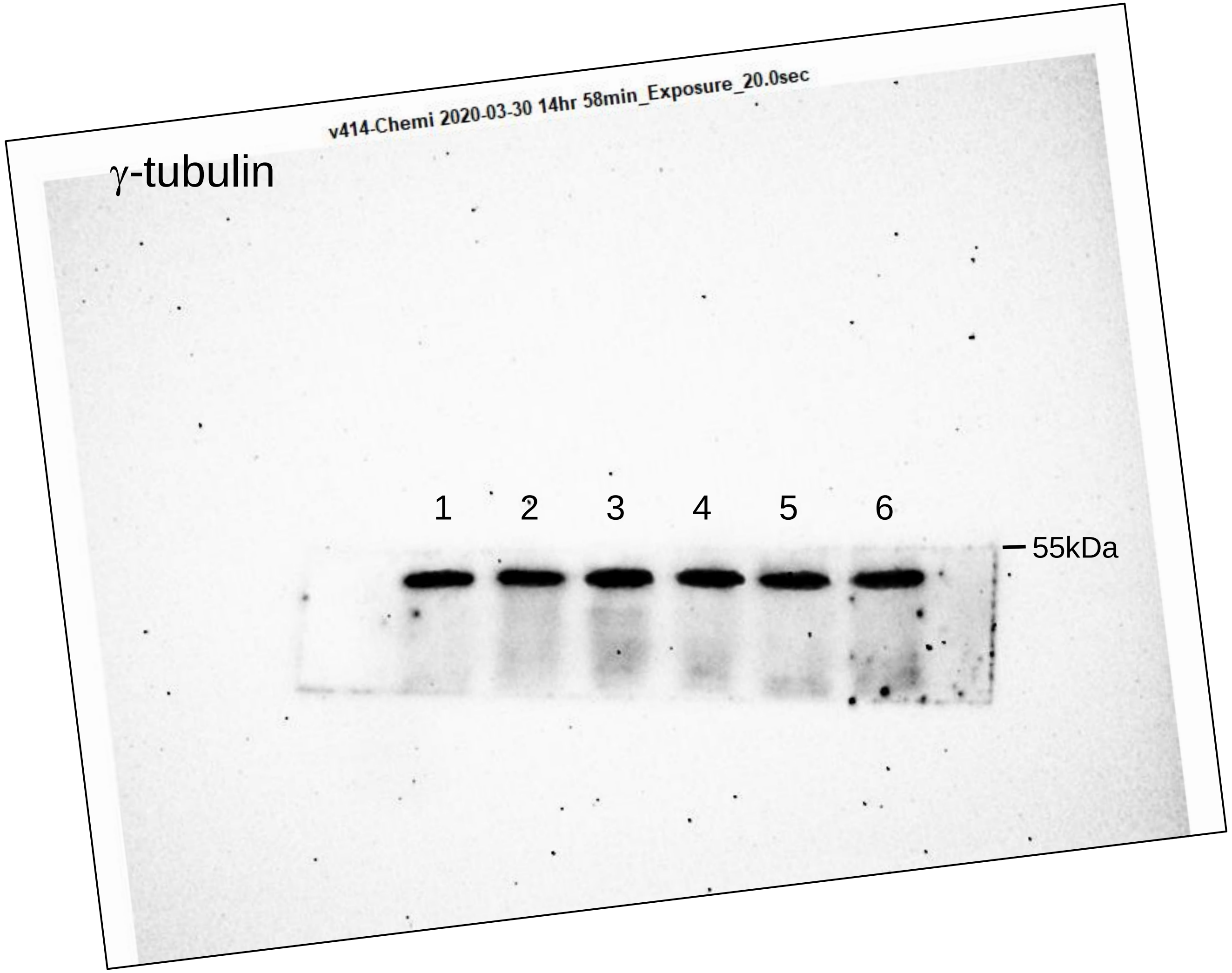

Fig. 2g

Lanes	1	2	3	4	5	6
HBx (μg)	0	0.5	0.5	0.5	0.5	0.5
ATRA (μM)	0	0	5	5	5	5
SC shRNA (μg)	0.5	0.5	0.5	0.48	0.4	0
p53 shRNA (μg)	0	0	0	0.02	0.1	0.5

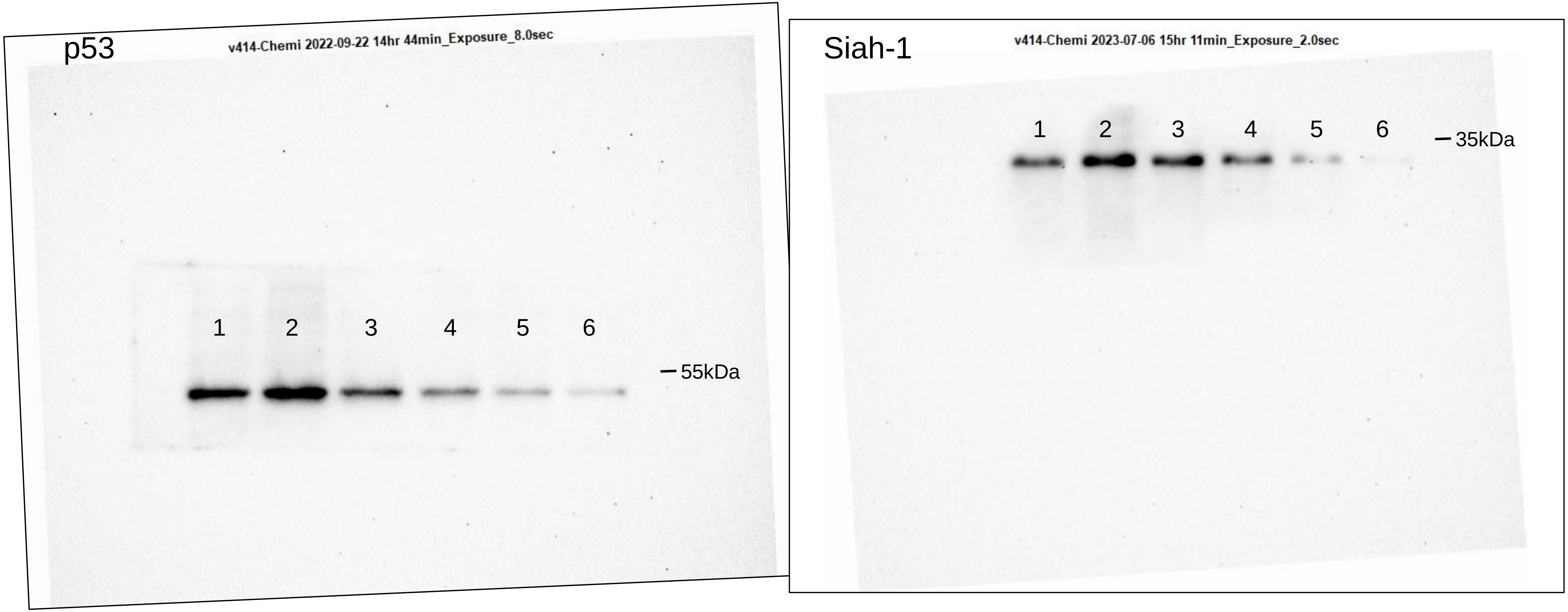

Fig. 2g

Lanes	1	2	3	4	5	6
HBx (μg)	0	0.5	0.5	0.5	0.5	0.5
ATRA (μM)	0	0	5	5	5	5
SC shRNA (μg)	0.5	0.5	0.5	0.48	0.4	0
p53 shRNA (μg)	0	0	0	0.02	0.1	0.5

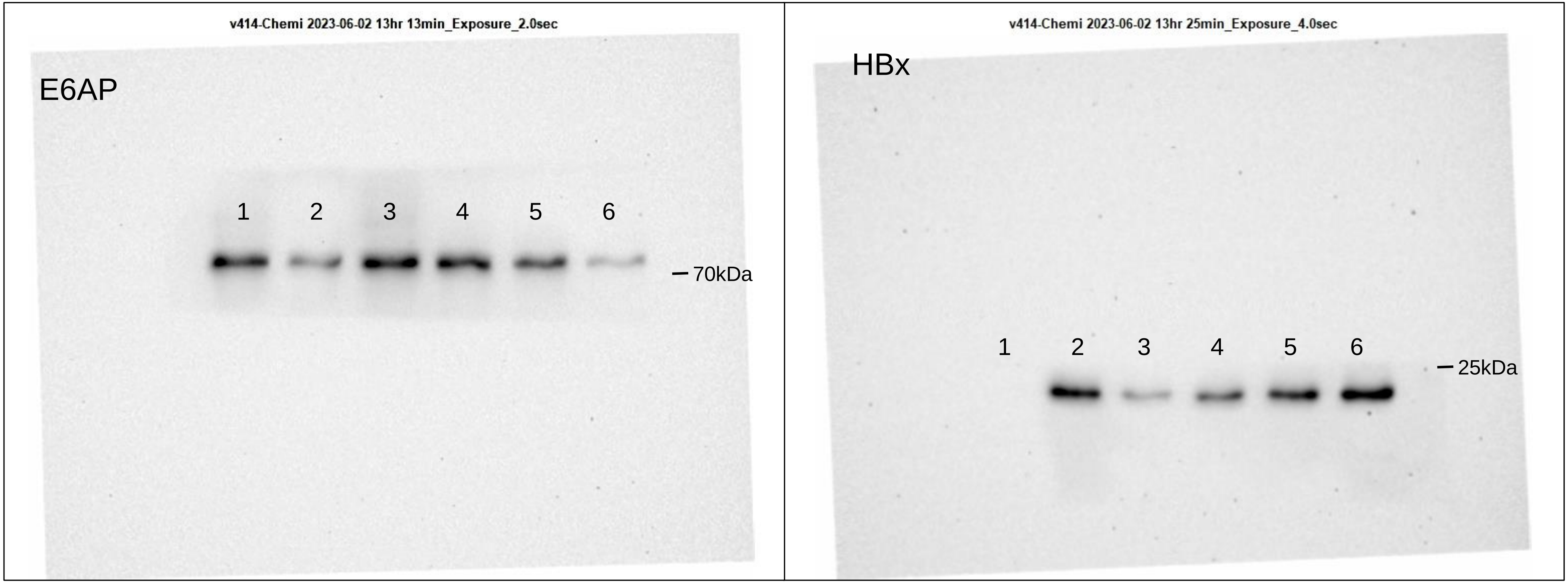

Fig. 2g

Lanes	1	2	3	4	5	6
HBx (μg)	0	0.5	0.5	0.5	0.5	0.5
ATRA (μM)	0	0	5	5	5	5
SC shRNA (μg)	0.5	0.5	0.5	0.48	0.4	0
p53 shRNA (μg)	0	0	0	0.02	0.1	0.5

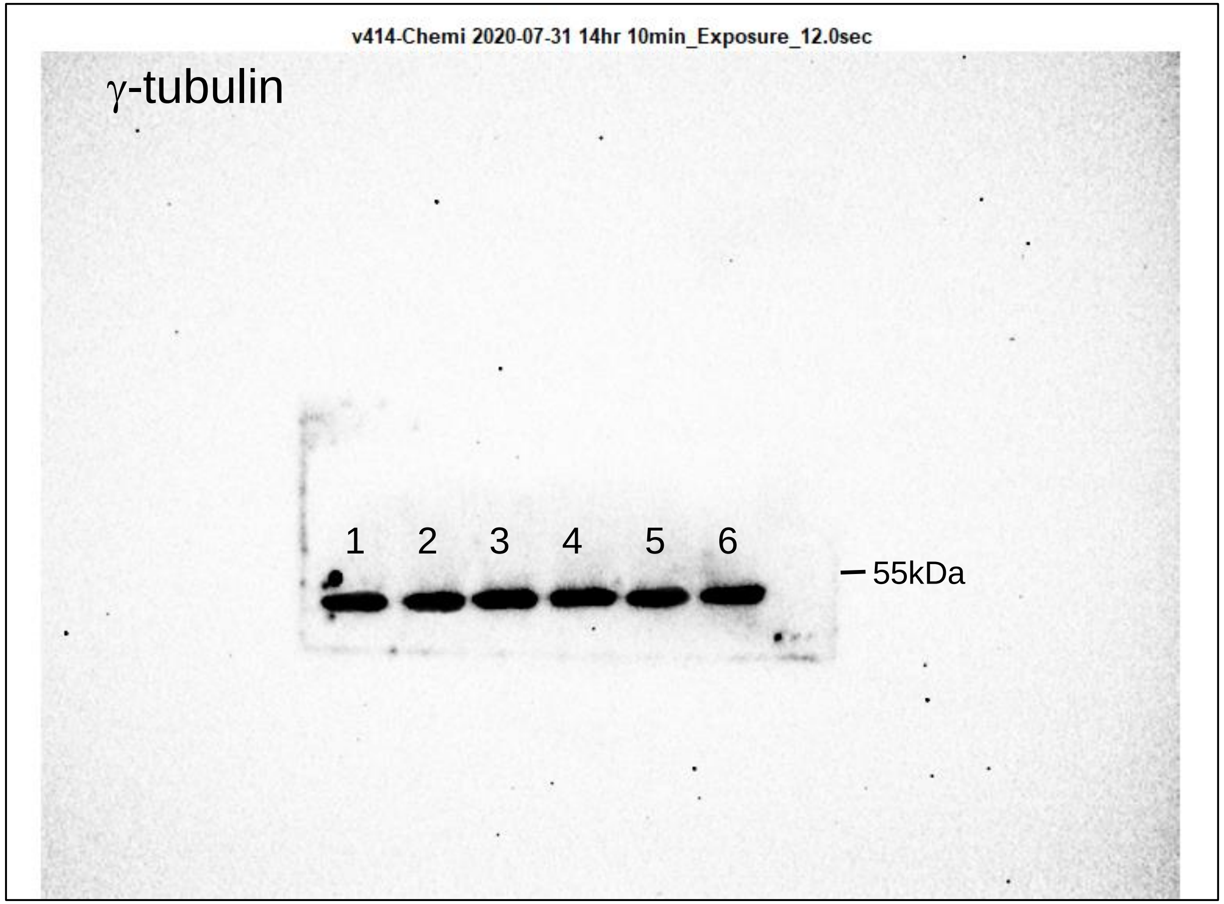

Fig. 2h

Lanes	1	2	3	4	5	6
HBx (μg)	0	0.5	0.5	0.5	0.5	0.5
p53 (μg)	0.1	0.1	0.1	0.1	0.1	0.1
ATRA (μM)	0	0	0.1	1.0	2.0	5.0

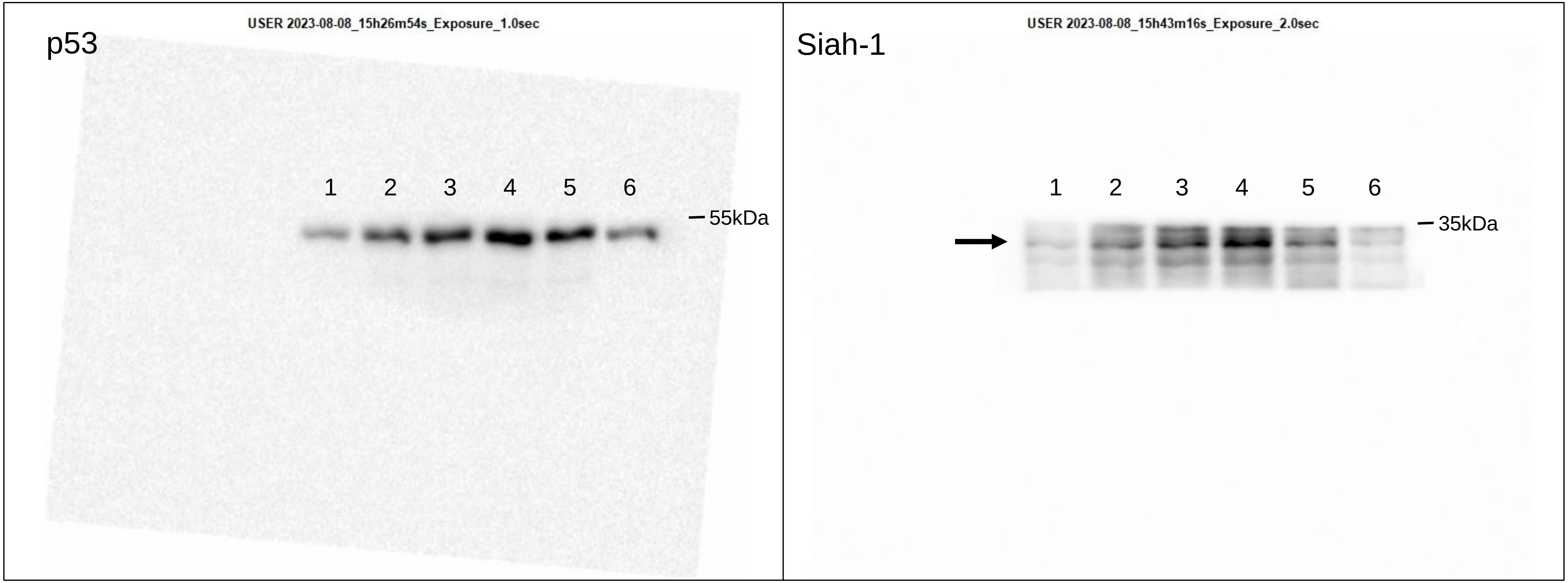

Fig. 2h

Lanes	1	2	3	4	5	6
HBx (μg)	0	0.5	0.5	0.5	0.5	0.5
p53 (μg)	0.1	0.1	0.1	0.1	0.1	0.1
ATRA (μM)	0	0	0.1	1.0	2.0	5.0

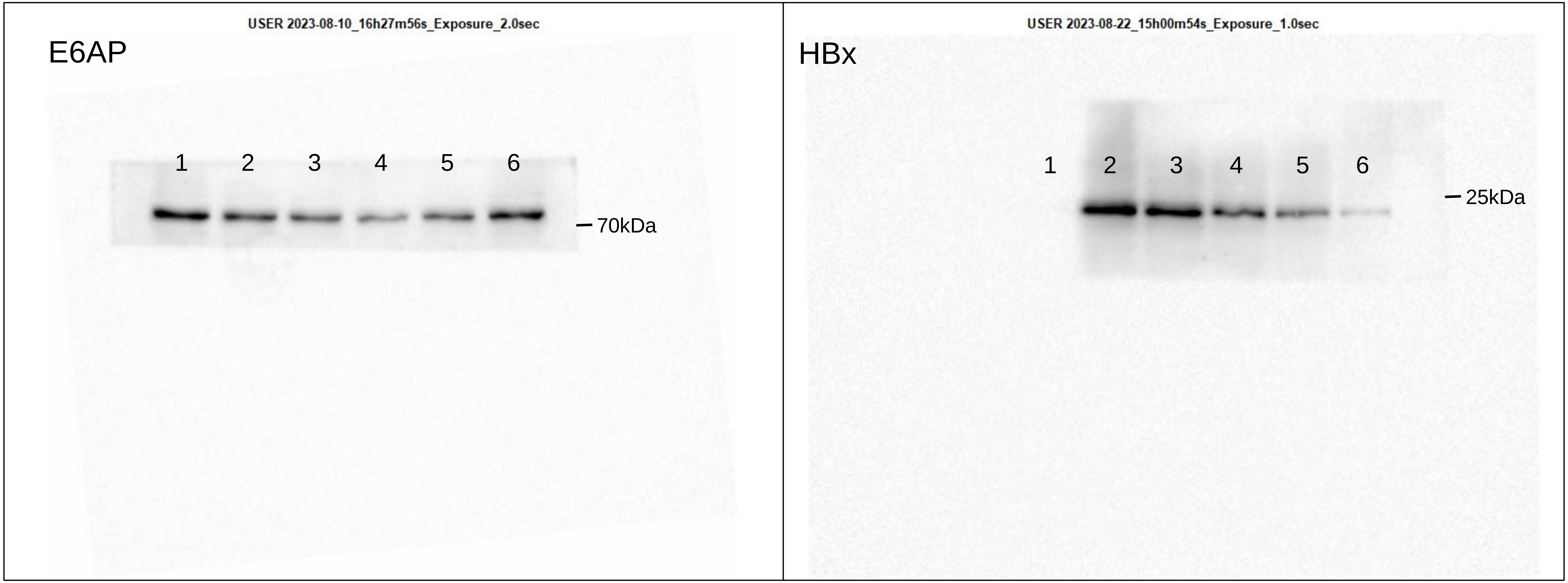

Fig. 2h

Lanes	1	2	3	4	5	6
HBx (μg)	0	0.5	0.5	0.5	0.5	0.5
p53 (μg)	0.1	0.1	0.1	0.1	0.1	0.1
ATRA (μM)	0	0	0.1	1.0	2.0	5.0

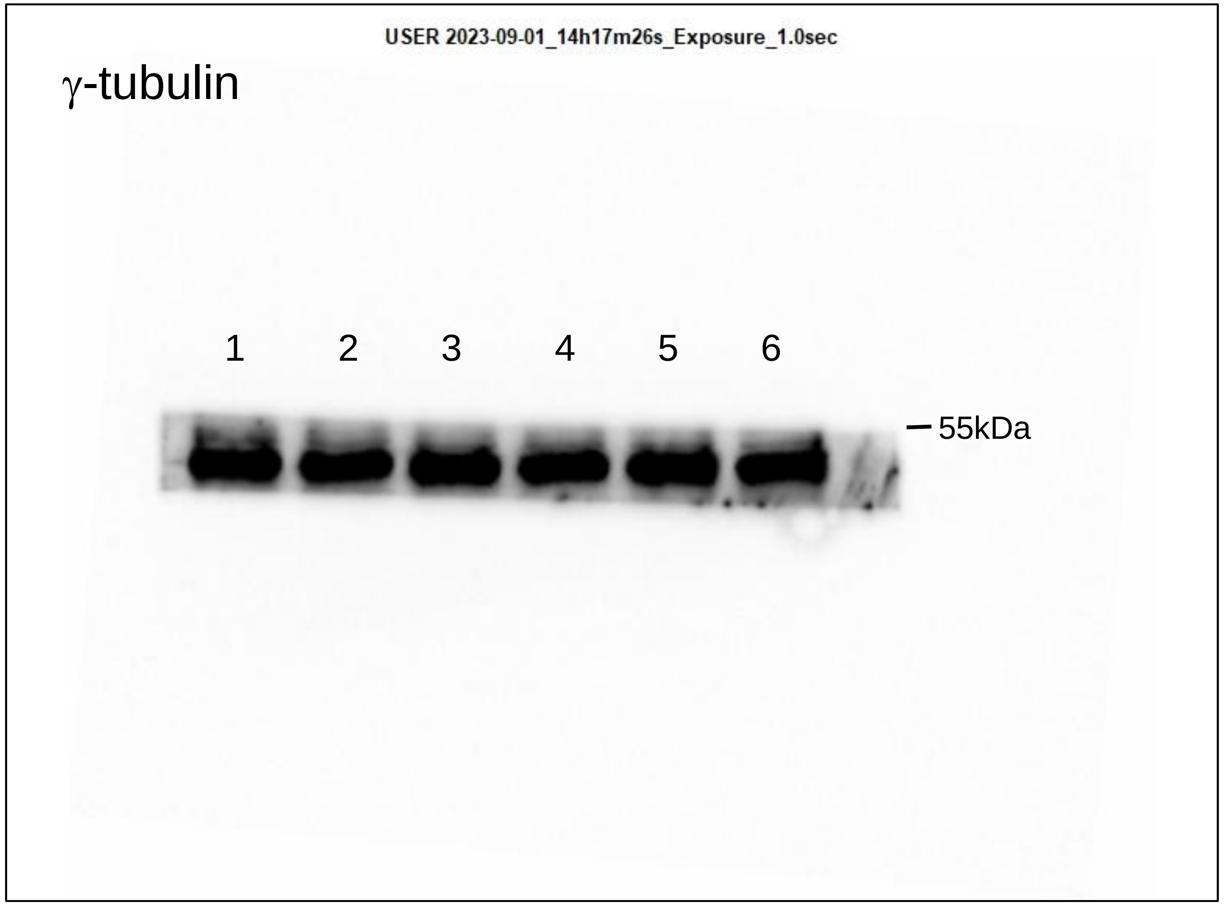

Fig. 3b

Lanes	1	2	3	4	5
HBx (μg)	0	0.5	0.5	0.5	0.5
ATRA (μM)	0	0	5	5	5
SC shRNA (μg)	1.0	1.0	1.0	0.9	0
E6AP shRNA (μg)	0	0	0	0.1	1.0

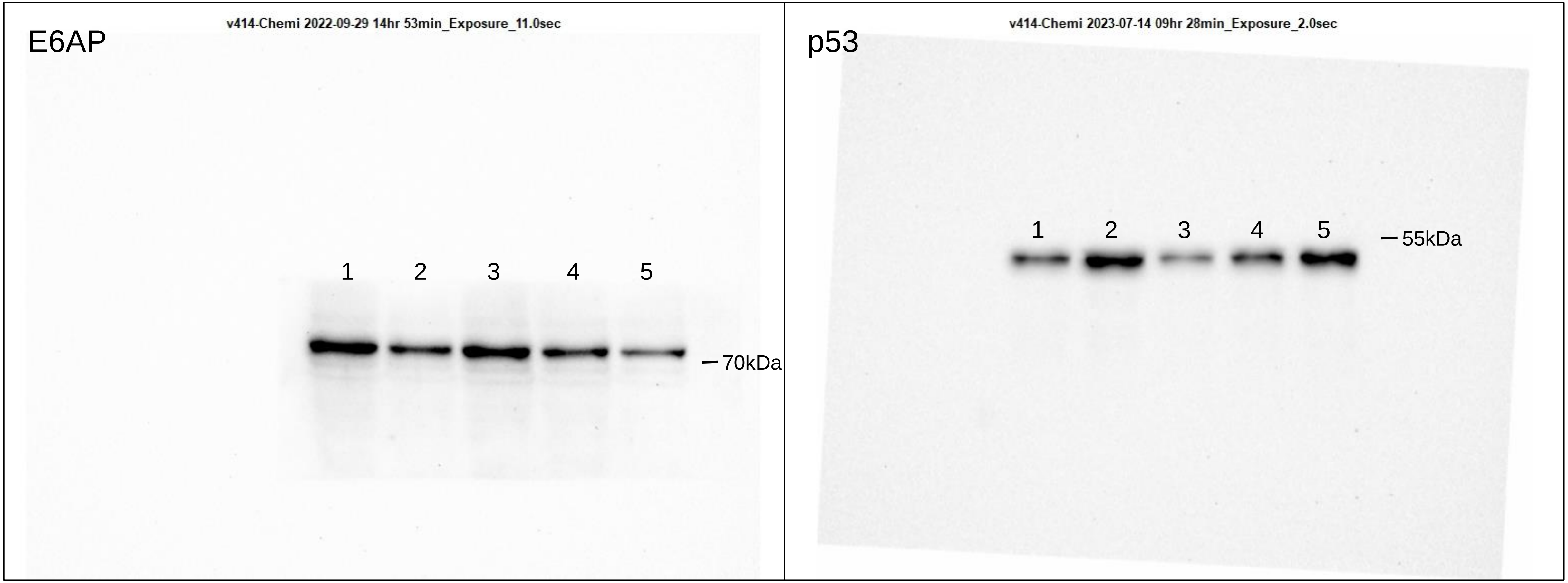

Fig. 3b

Lanes	1	2	3	4	5
HBx (μg)	0	0.5	0.5	0.5	0.5
ATRA (μM)	0	0	5	5	5
SC shRNA (μg)	1.0	1.0	1.0	0.9	0
E6AP shRNA (μg)	0	0	0	0.1	1.0

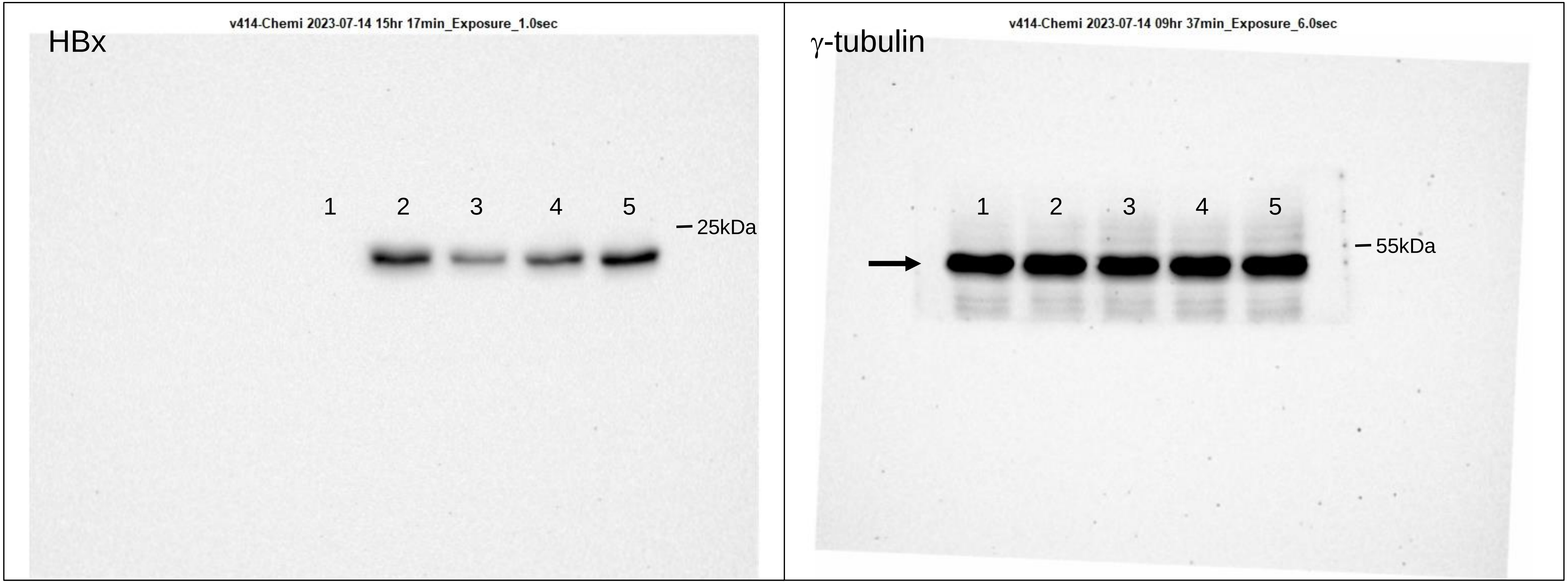

Fig. 3c

Lanes	1	2	3	4	5
HBx (μg)	0	0.5	0.5	0.5	0.5
ATRA (μM)	0	0	5	5	5
p53 (μg)	0	0.1	0.1	0.1	0.1
SC shRNA (μg)	1.0	1.0	1.0	0.9	0
E6AP shRNA (μg)	0	0	0	0.1	1.0

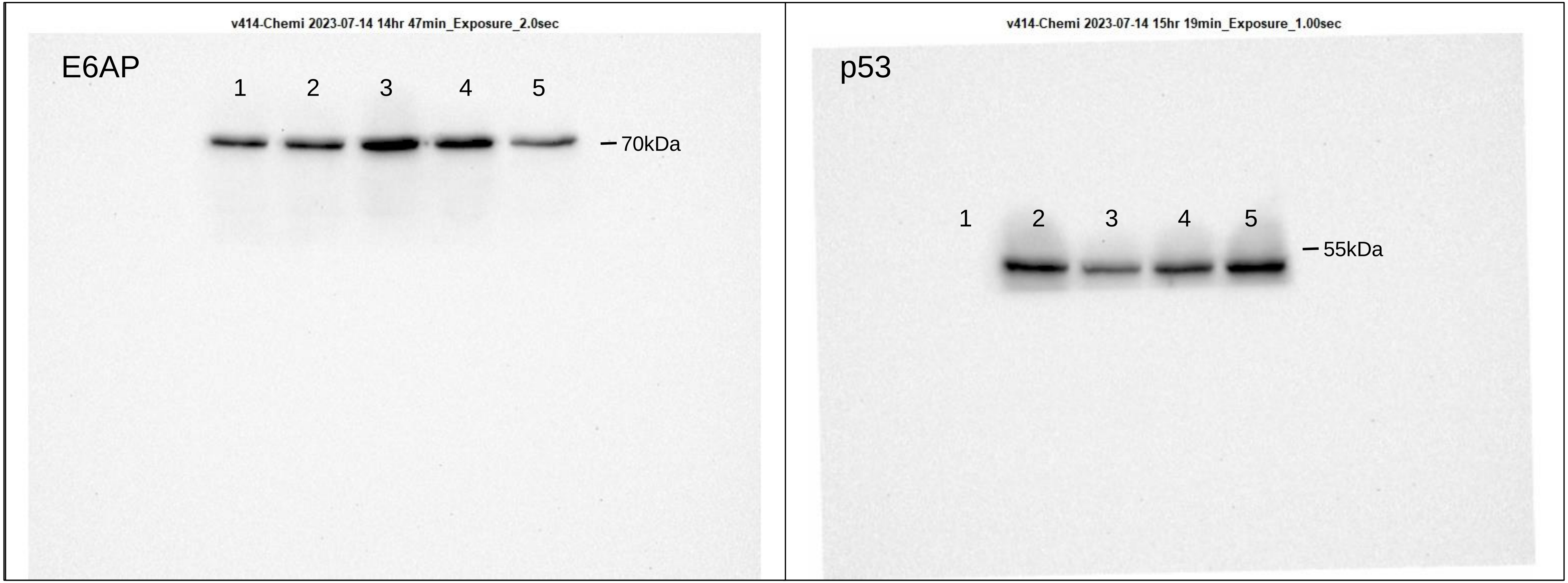

Fig. 3c

Lanes	1	2	3	4	5
HBx (μg)	0	0.5	0.5	0.5	0.5
ATRA (μM)	0	0	5	5	5
p53 (μg)	0	0.1	0.1	0.1	0.1
SC shRNA (μg)	1.0	1.0	1.0	0.9	0
E6AP shRNA (μg)	0	0	0	0.1	1.0

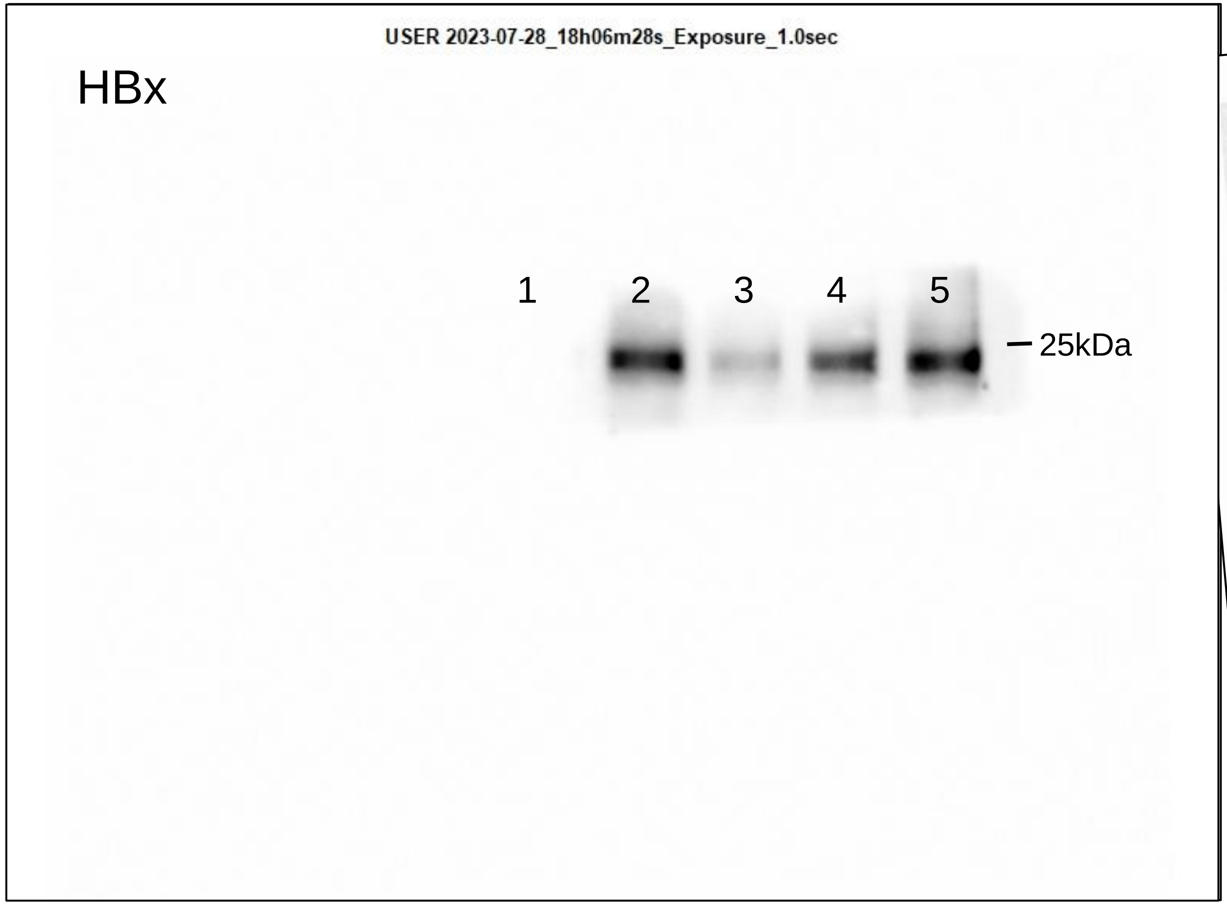

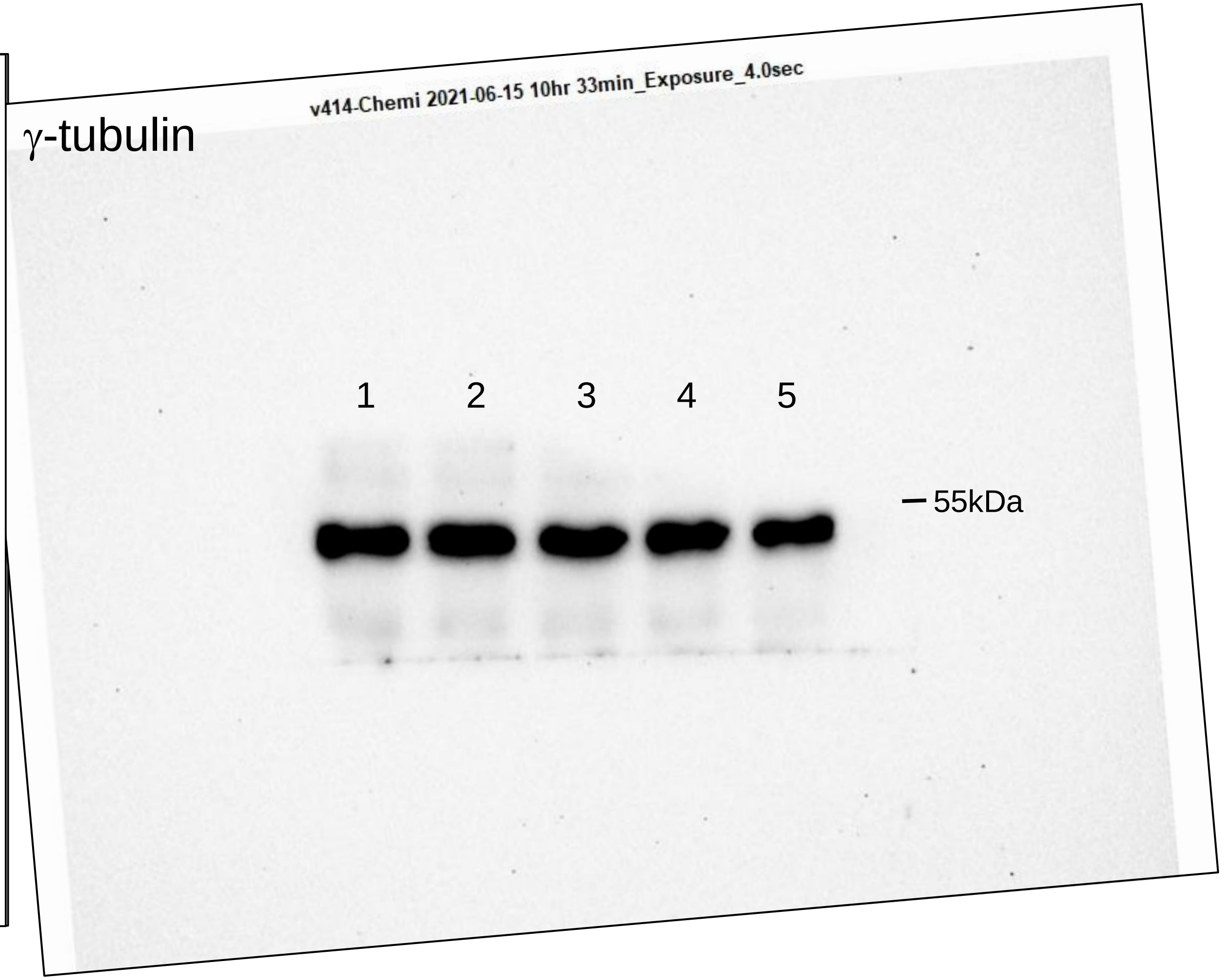

Fig. 3d

Lanes	1	2	3	4	5
HBx (μg)	0	0.5	0.5	0.5	0.5
ATRA (μM)	0	0	5	5	5
Heclin (μM)	0	0	0	10	20

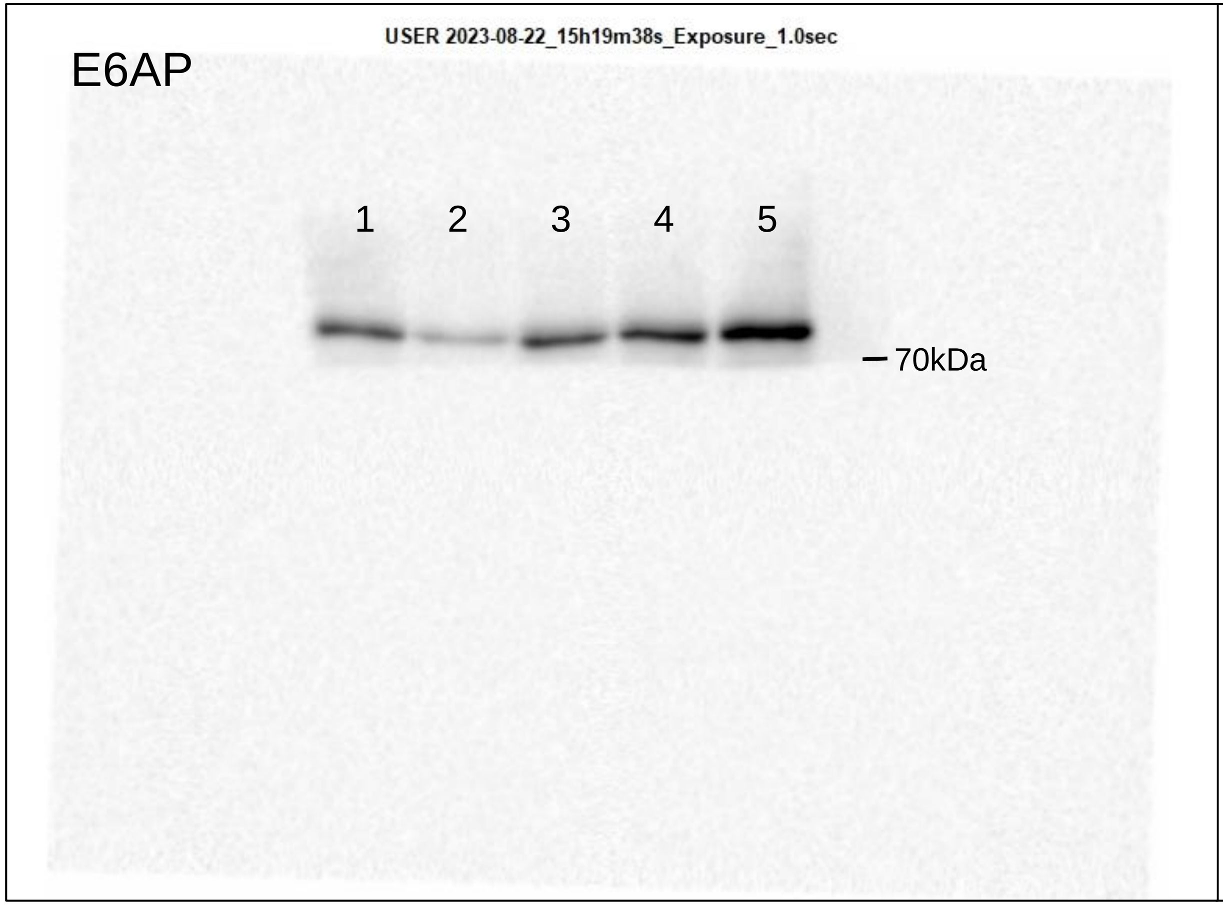

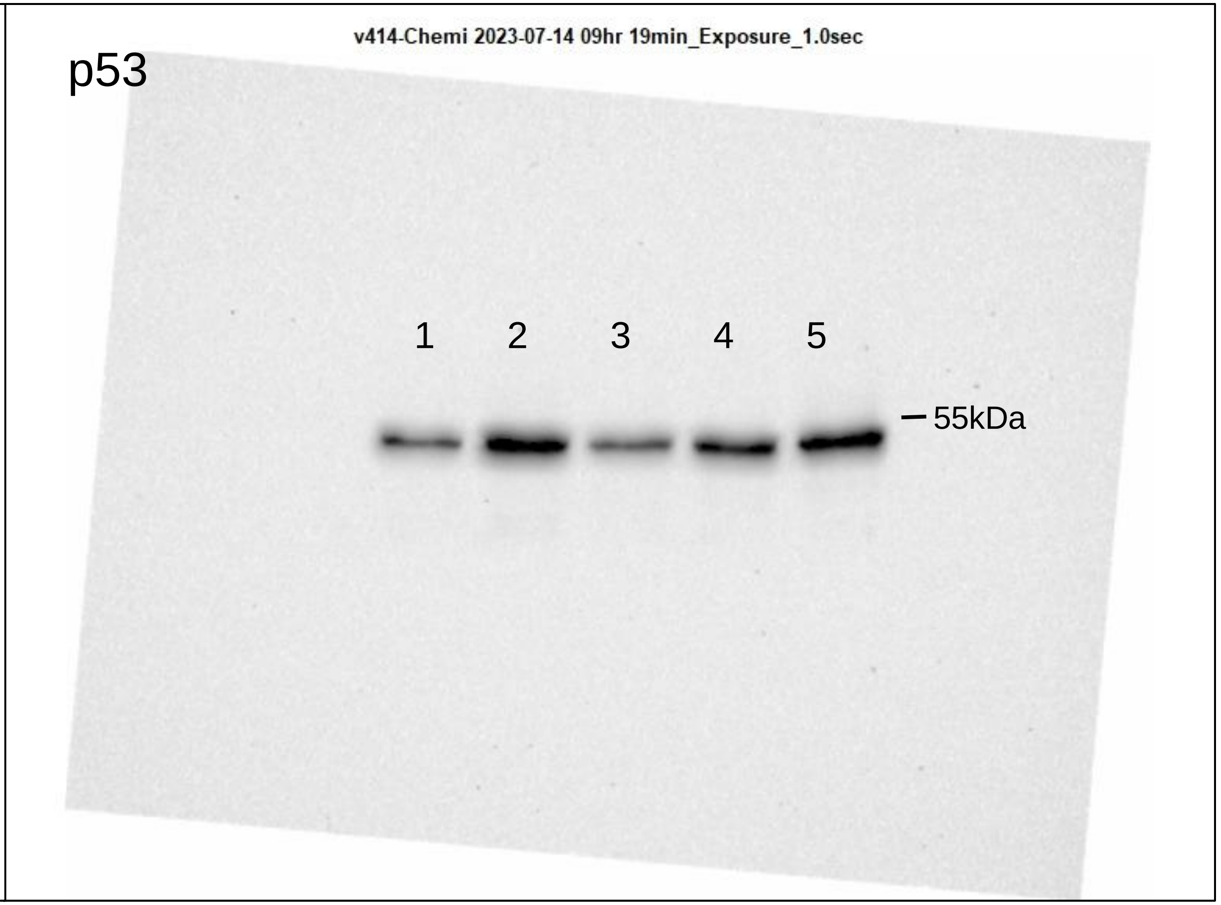

Fig. 3d

Lanes	1	2	3	4	5
HBx (μg)	0	0.5	0.5	0.5	0.5
ATRA (μM)	0	0	5	5	5
Heclin (μM)	0	0	0	10	20

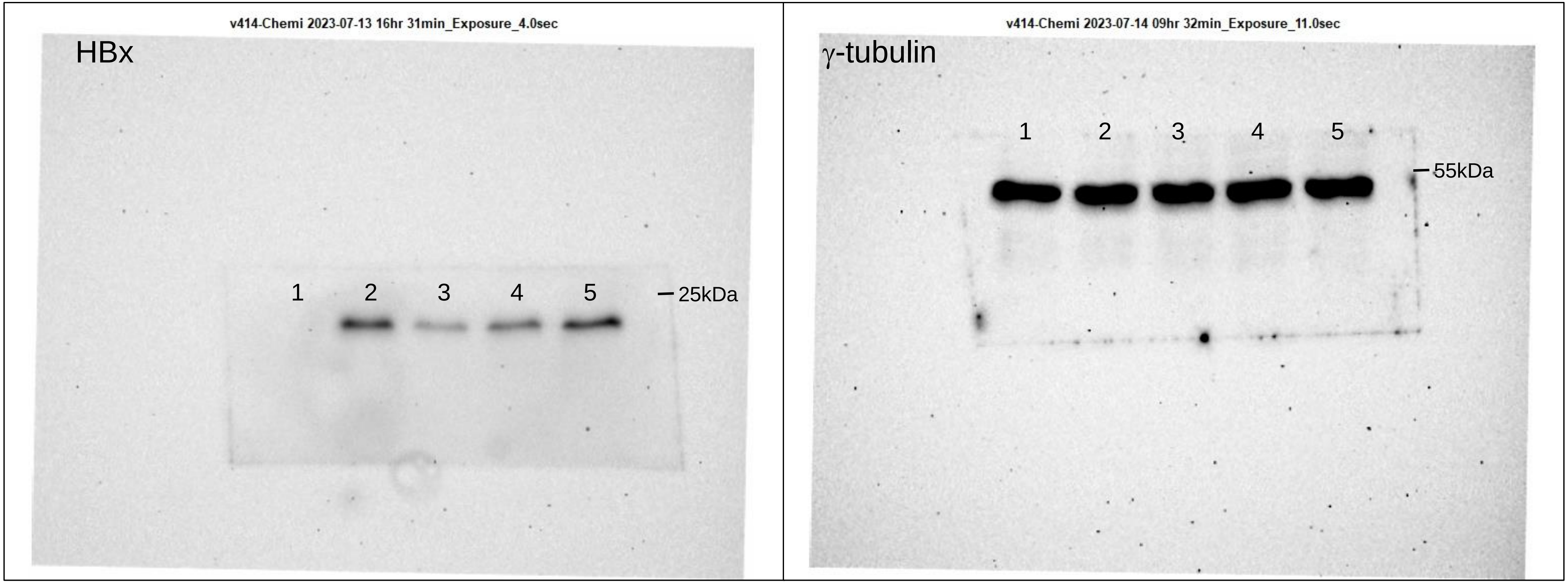

Fig. 3e

Lanes	1	2	3	4	5
HBx (μg)	0	0.5	0.5	0.5	0.5
ATRA (μM)	0	0	5	5	5
E6AP C833A (μM)	0	0	0	0.1	1.0

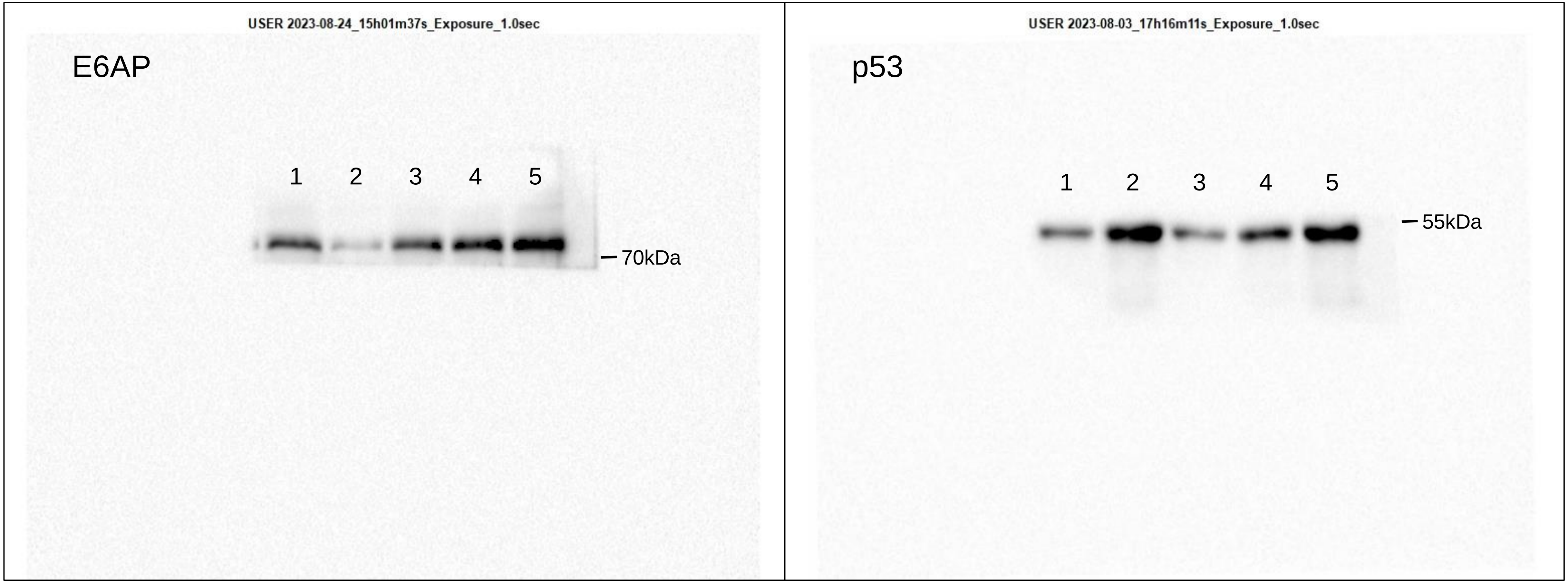

Fig. 3e

Lanes	1	2	3	4	5
HBx (μg)	0	0.5	0.5	0.5	0.5
ATRA (μM)	0	0	5	5	5
E6AP C833A (μM)	0	0	0	0.1	1.0

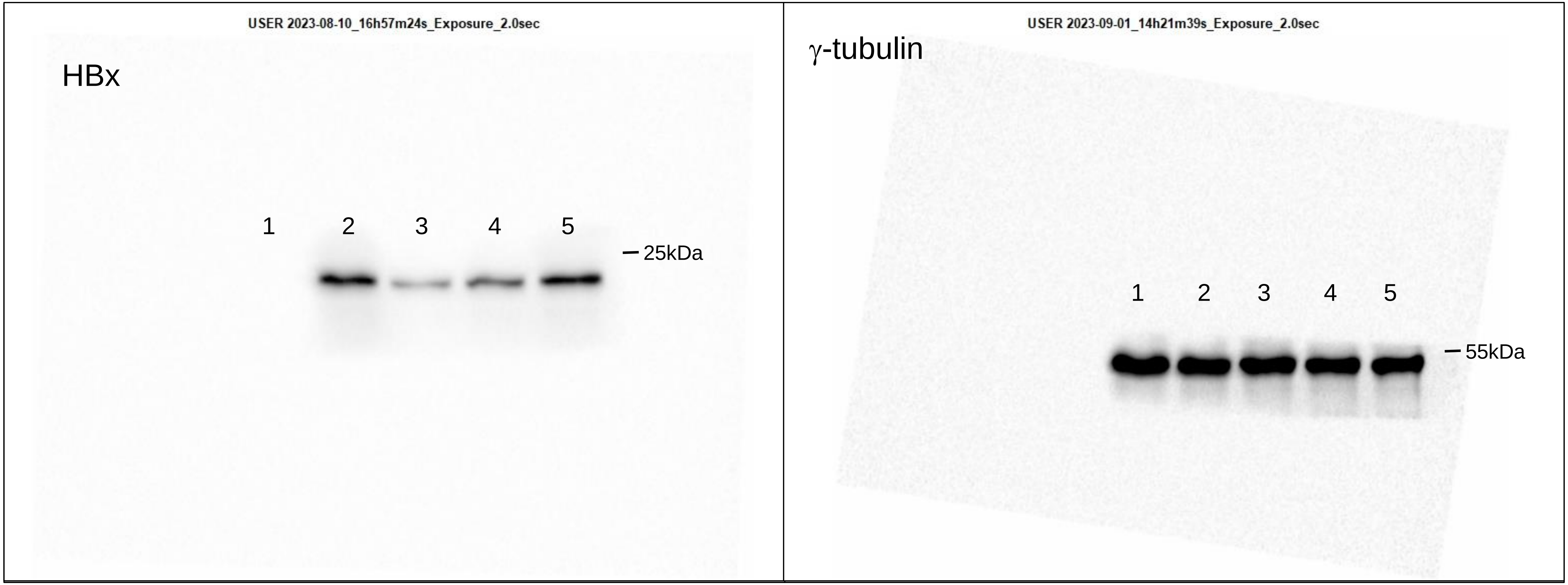

Fig. 4a

	HepG2				Hep3B			
Lane	1	2	3	4	5	6	7	8
HBx (μg)	0	0.5	0	0.5	0	0.5	0	0.5
ATRA (μM)	0	0	5	5	0	0	5	5

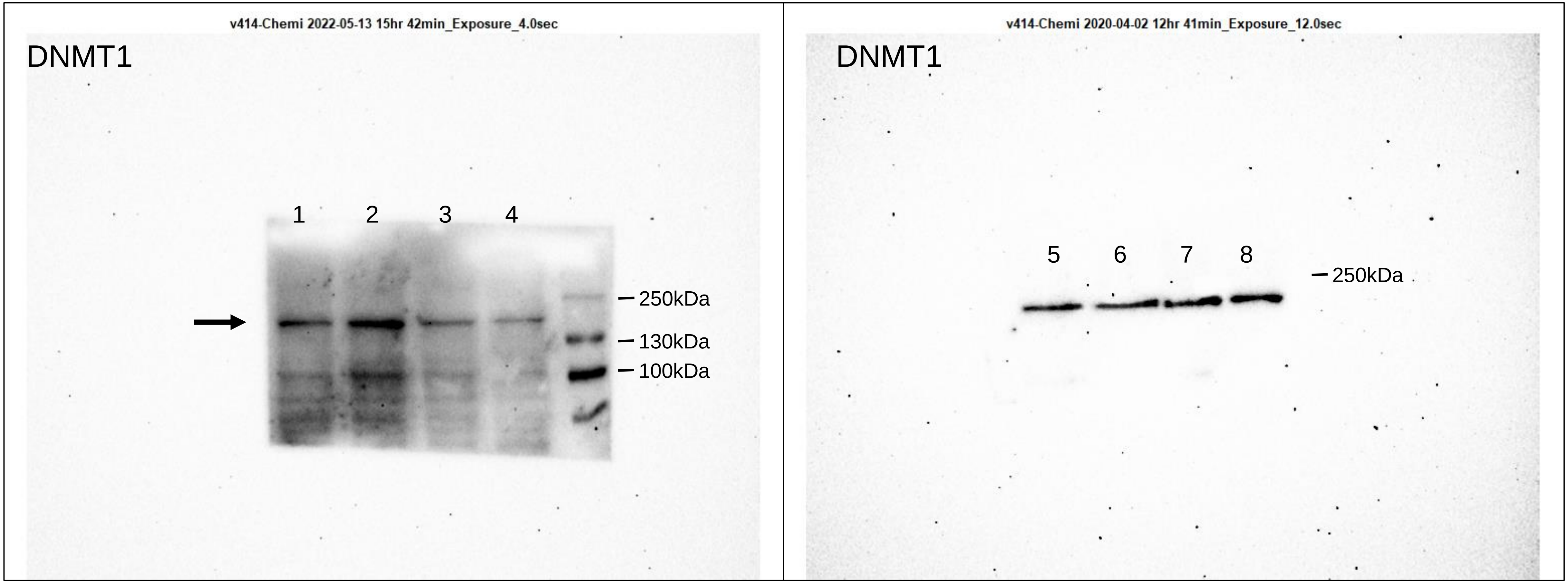

Fig. 4a

	HepG2				Hep3B			
Lane	1	2	3	4	5	6	7	8
HBx (μg)	0	0.5	0	0.5	0	0.5	0	0.5
ATRA (μM)	0	0	5	5	0	0	5	5

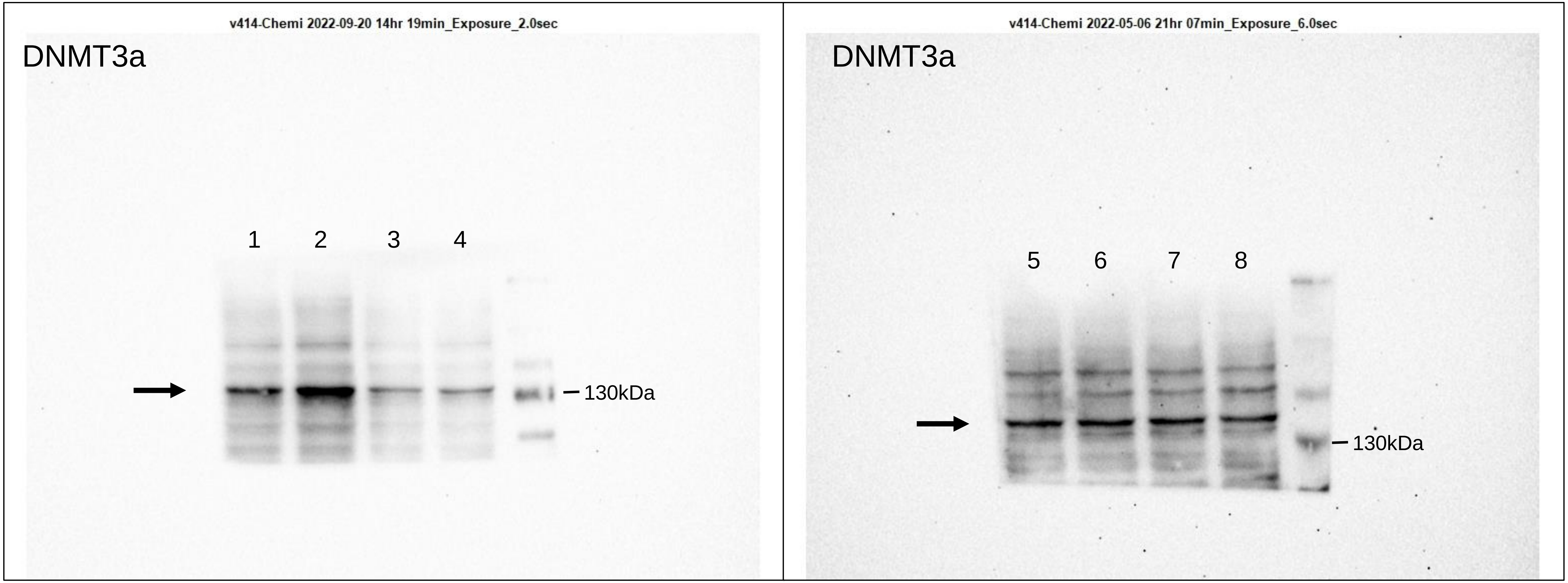

Fig. 4a

	HepG2				Hep3B			
Lane	1	2	3	4	5	6	7	8
HBx (μg)	0	0.5	0	0.5	0	0.5	0	0.5
ATRA (μM)	0	0	5	5	0	0	5	5

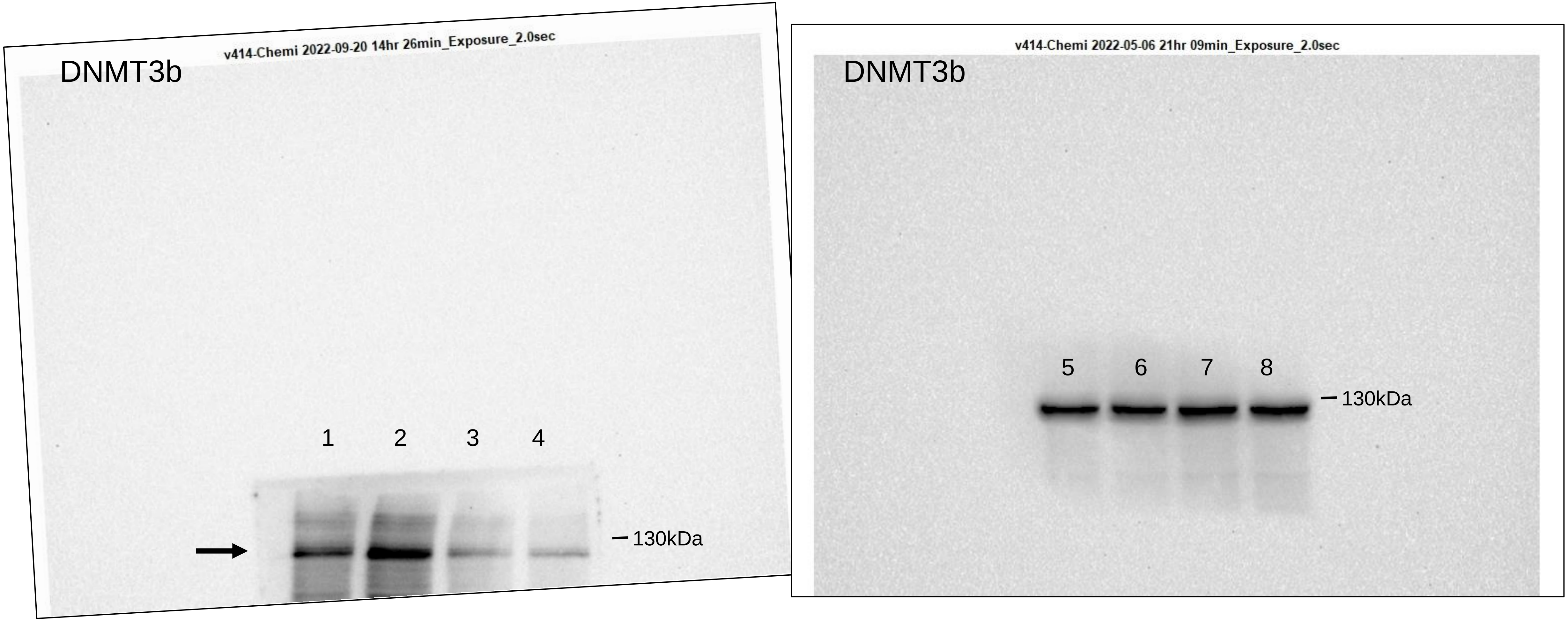

Fig. 4a

	HepG2				Hep3B			
Lane	1	2	3	4	5	6	7	8
HBx (μg)	0	0.5	0	0.5	0	0.5	0	0.5
ATRA (μM)	0	0	5	5	0	0	5	5

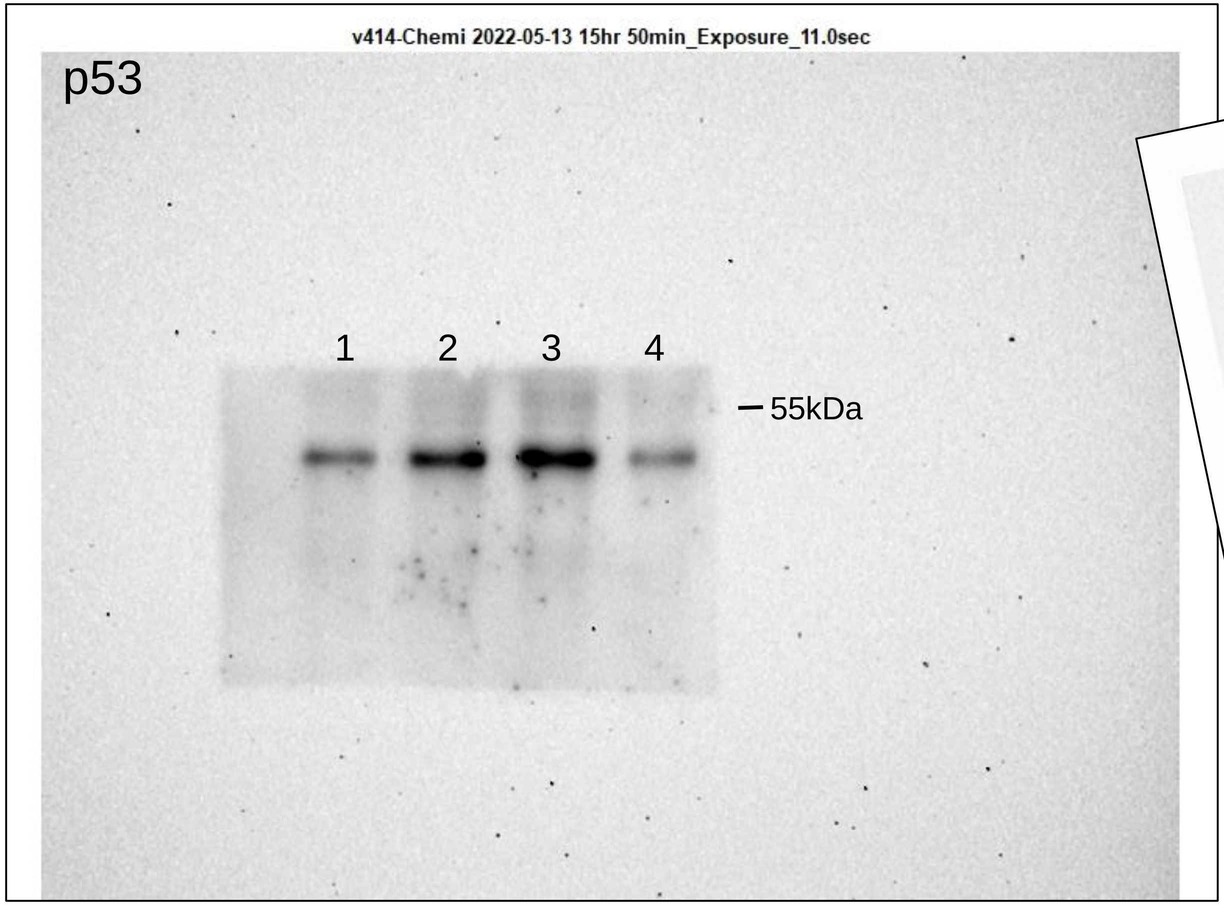

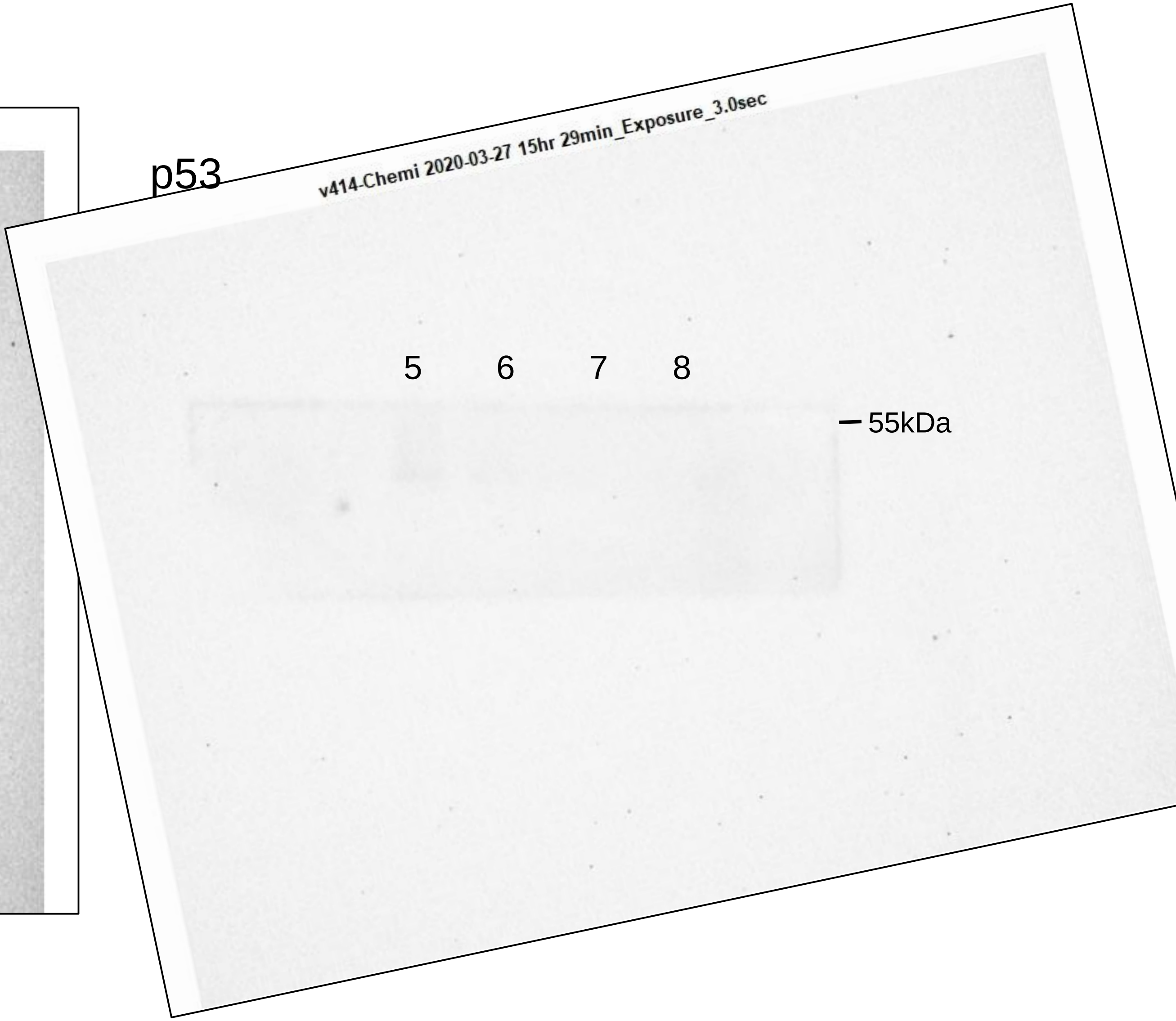

Fig. 4b

	HepG2				Hep3B			
Lane	1	2	3	4	5	6	7	8
HBx (μg)	0	0.5	0	0.5	0	0.5	0	0.5
ATRA (μM)	0	0	5	5	0	0	5	5

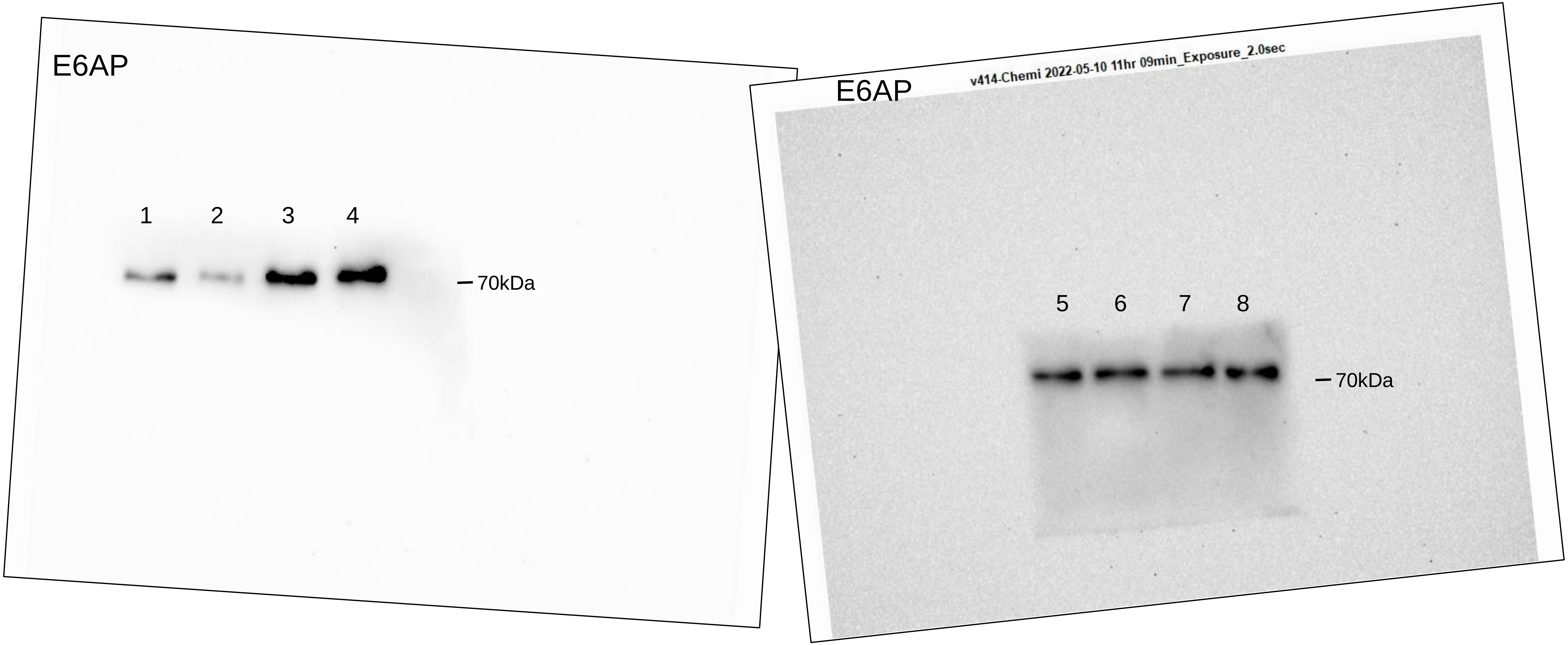

Fig. 4a

	HepG2				Hep3B			
Lane	1	2	3	4	5	6	7	8
HBx (μg)	0	0.5	0	0.5	0	0.5	0	0.5
ATRA (μM)	0	0	5	5	0	0	5	5

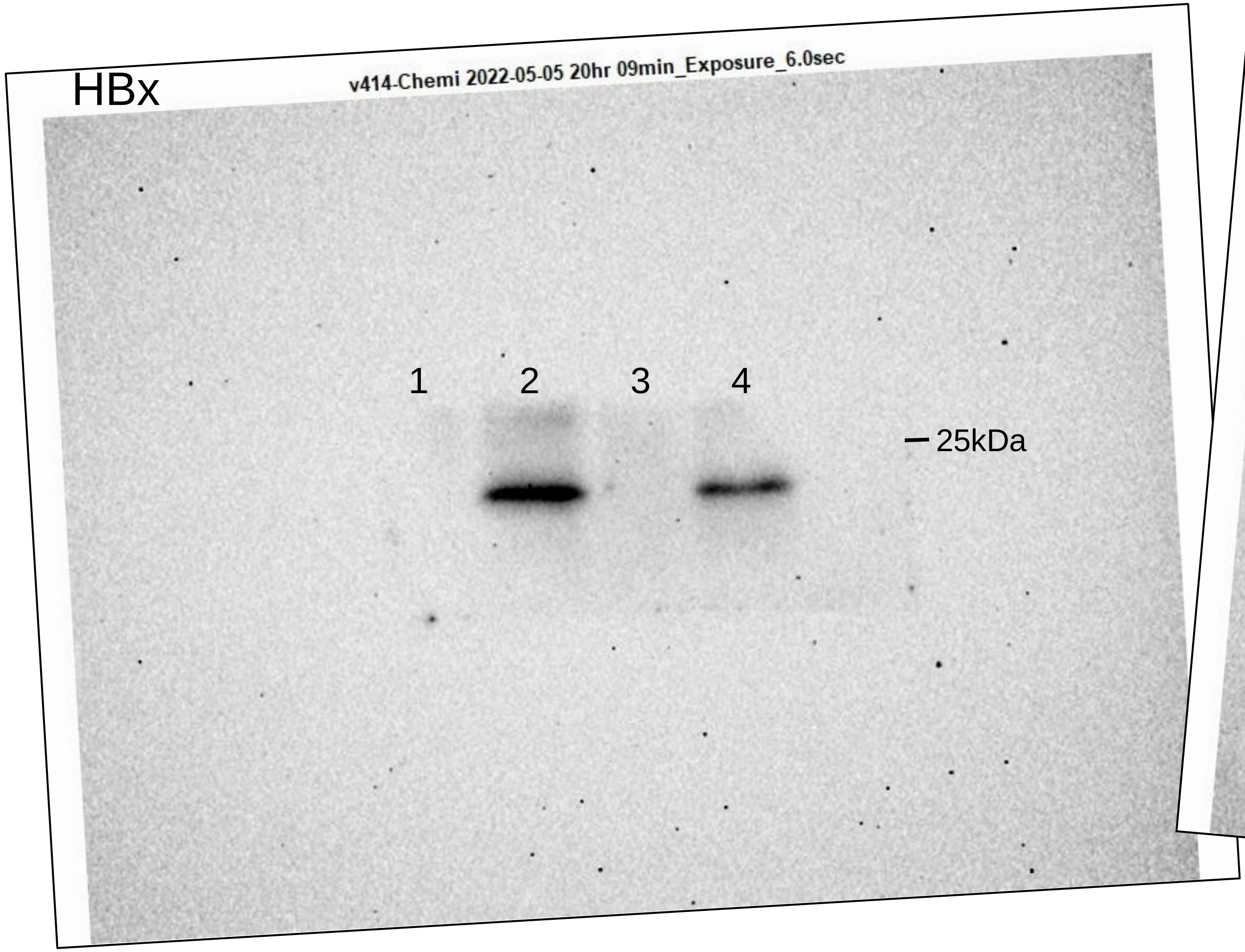

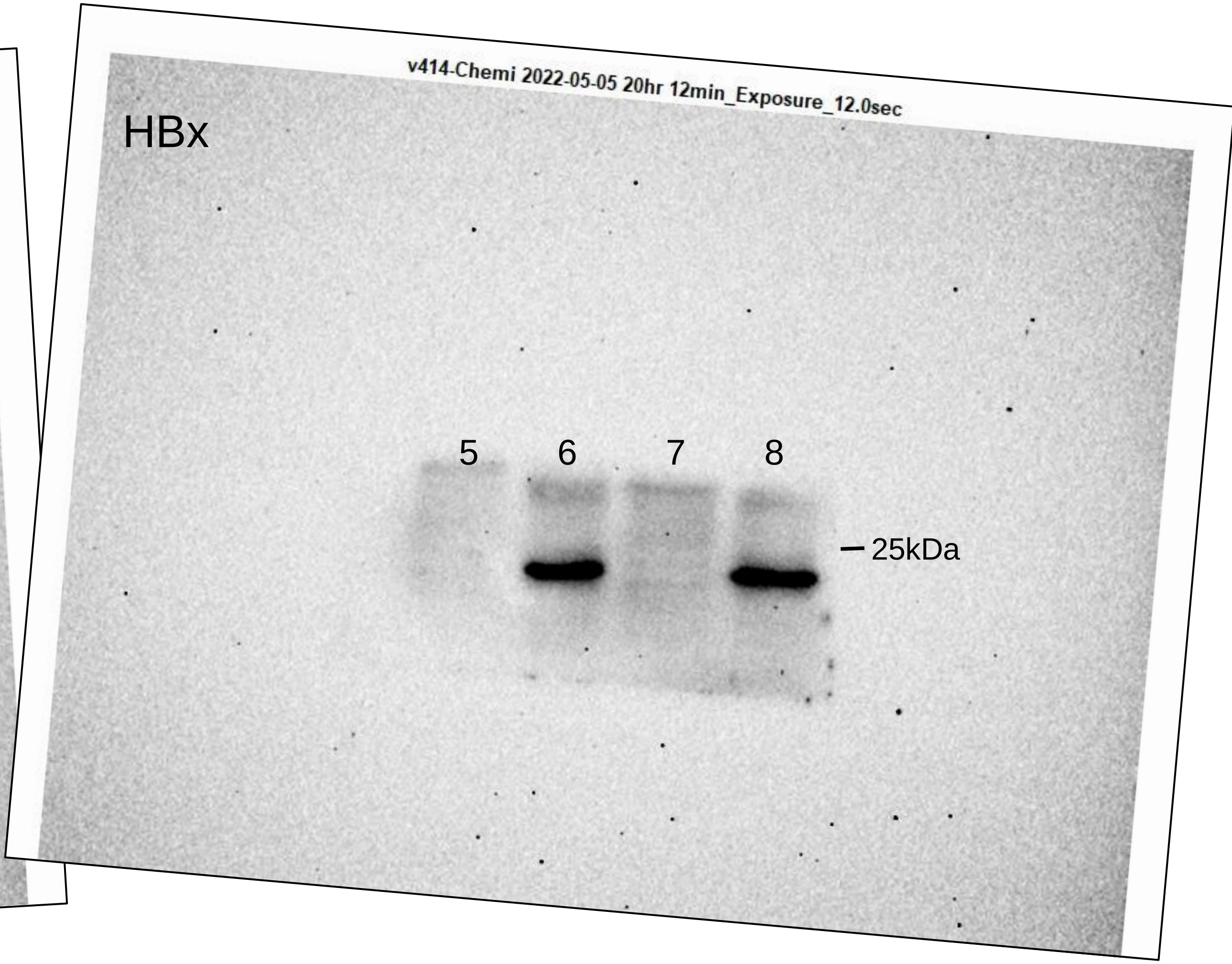

Fig. 4a

	HepG2				Hep3B			
Lane	1	2	3	4	5	6	7	8
HBx (μg)	0	0.5	0	0.5	0	0.5	0	0.5
ATRA (μM)	0	0	5	5	0	0	5	5

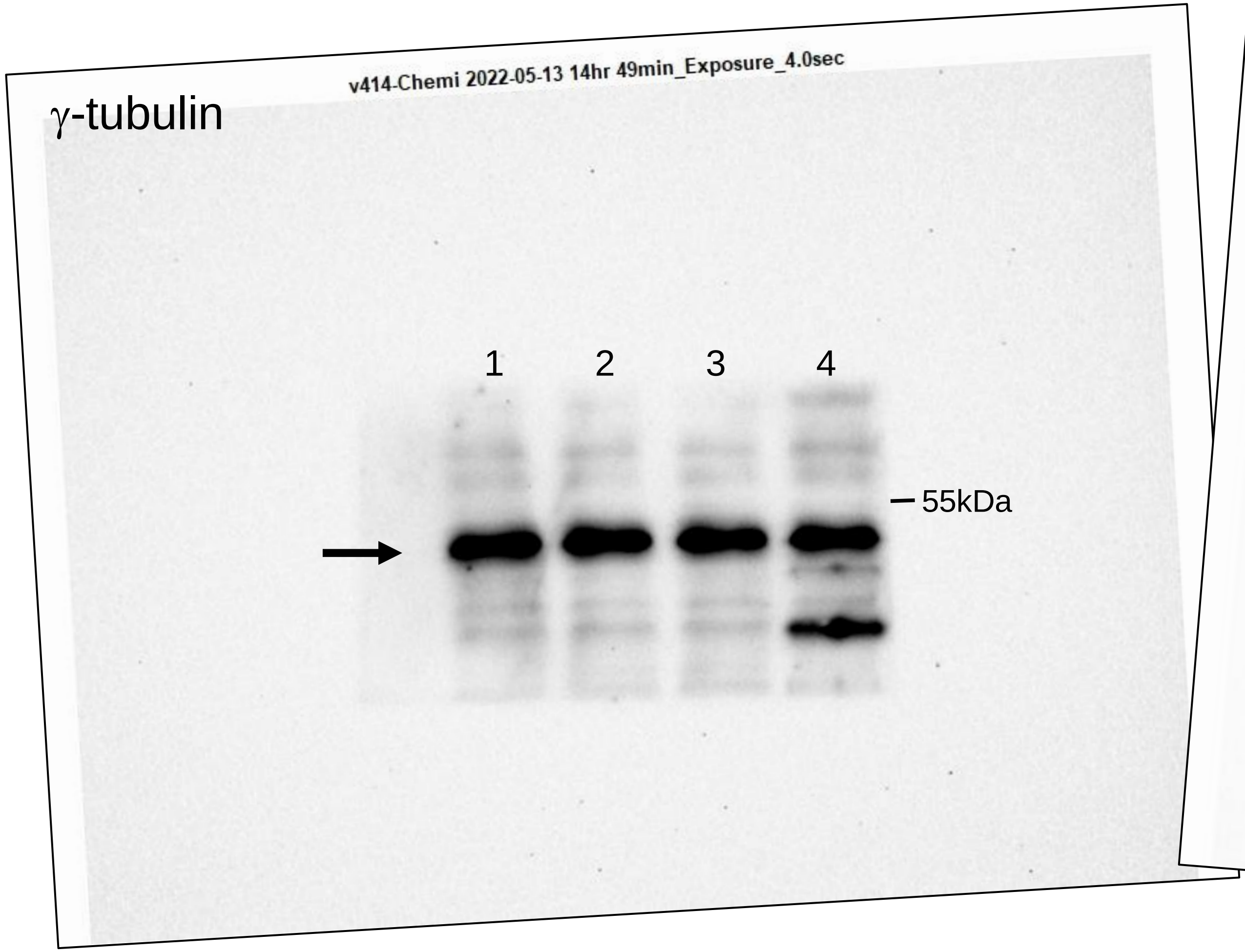

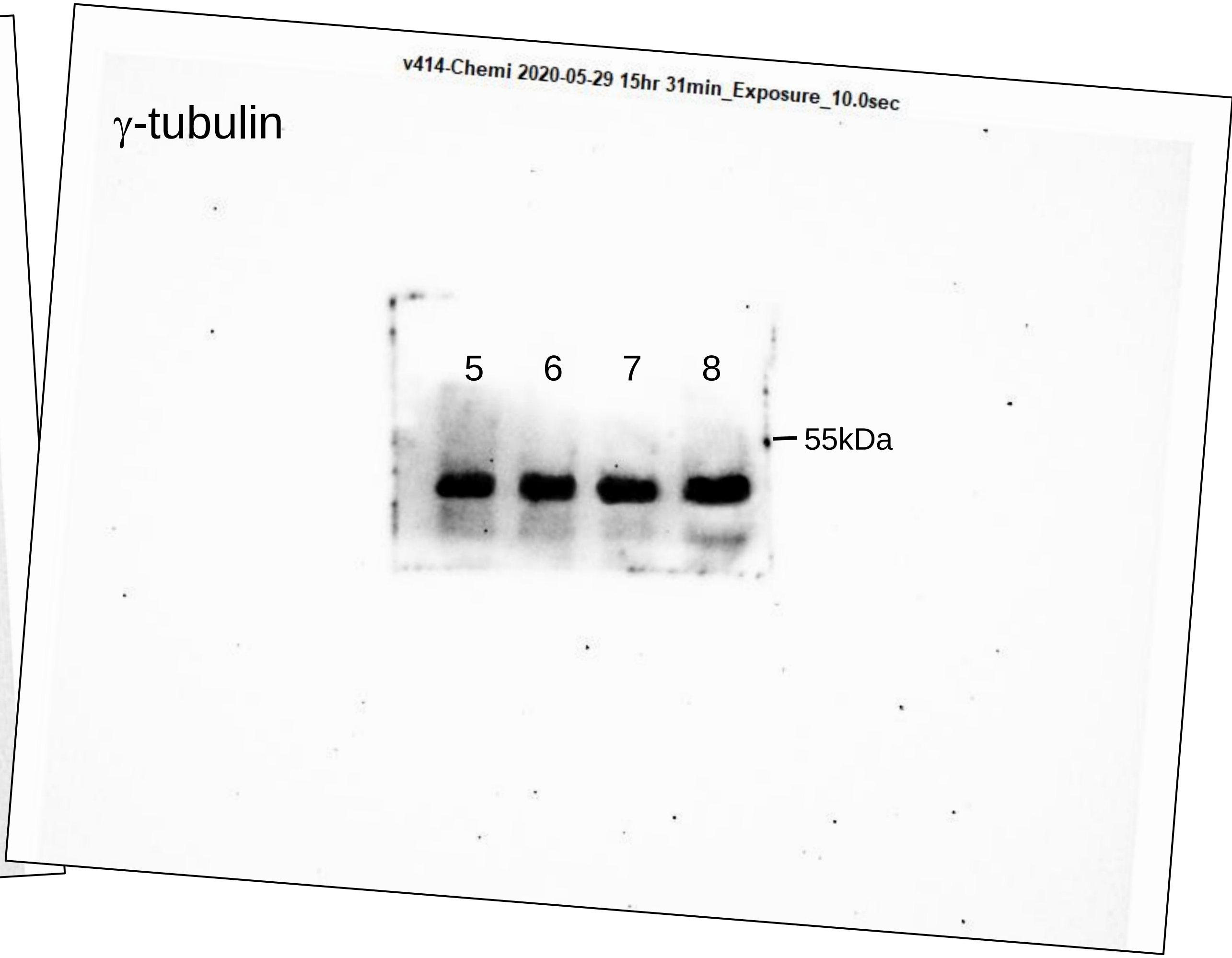

Fig. 5a

Lanes	1	2	3	4	5
CHX (min)	0	15	30	60	120

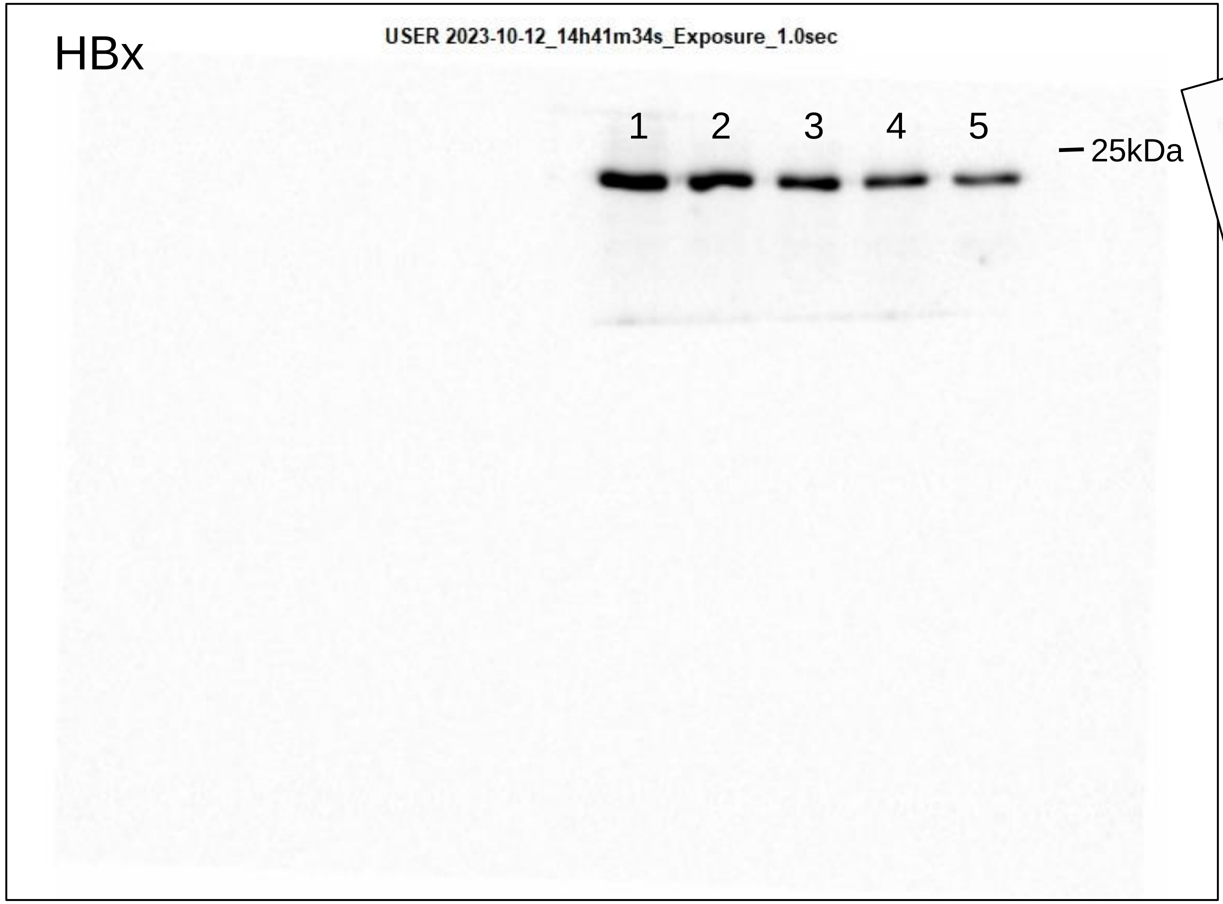

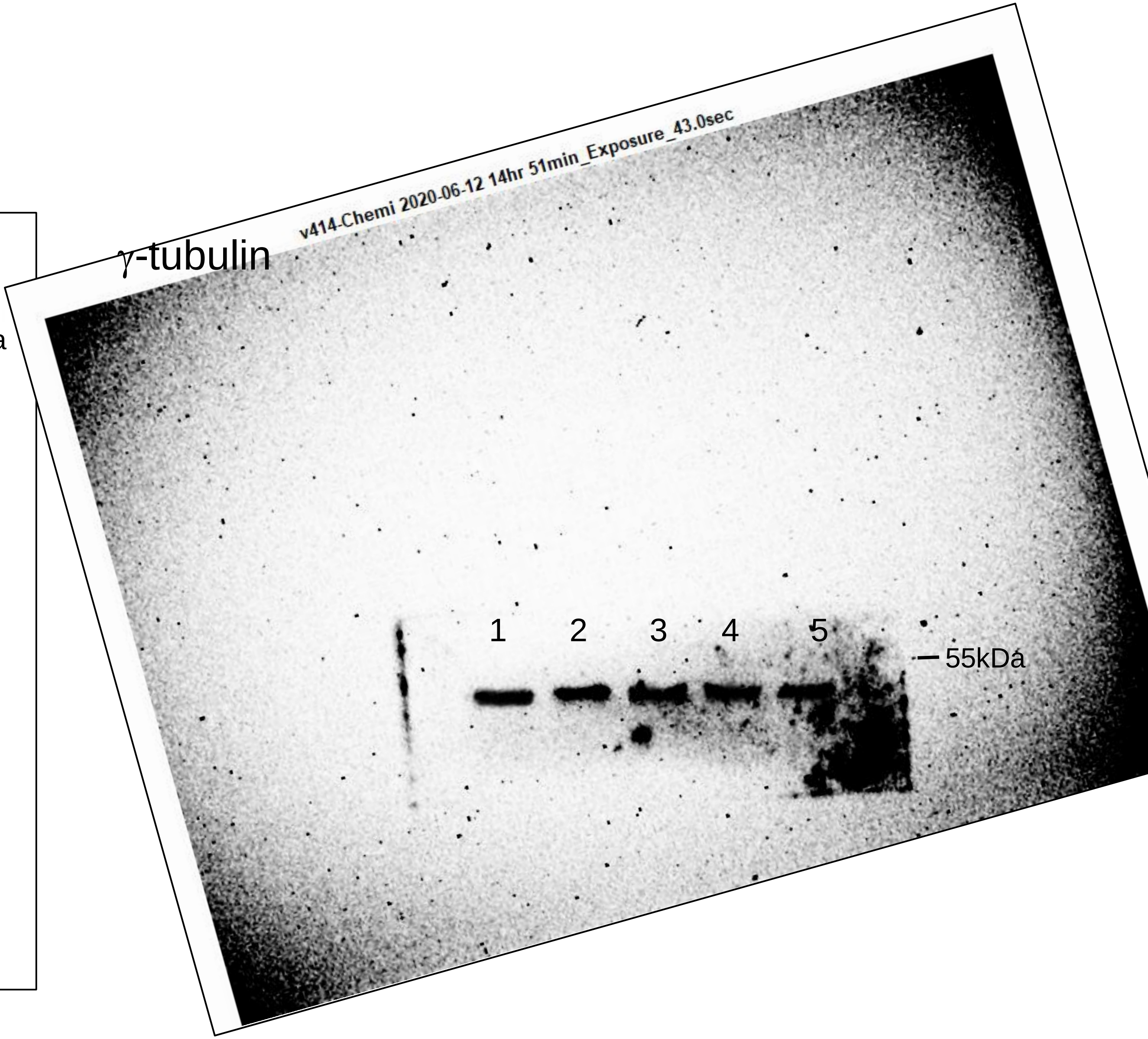

Fig. 5a

Lanes	1	2	3	4	5
CHX (min)	0	15	30	60	120

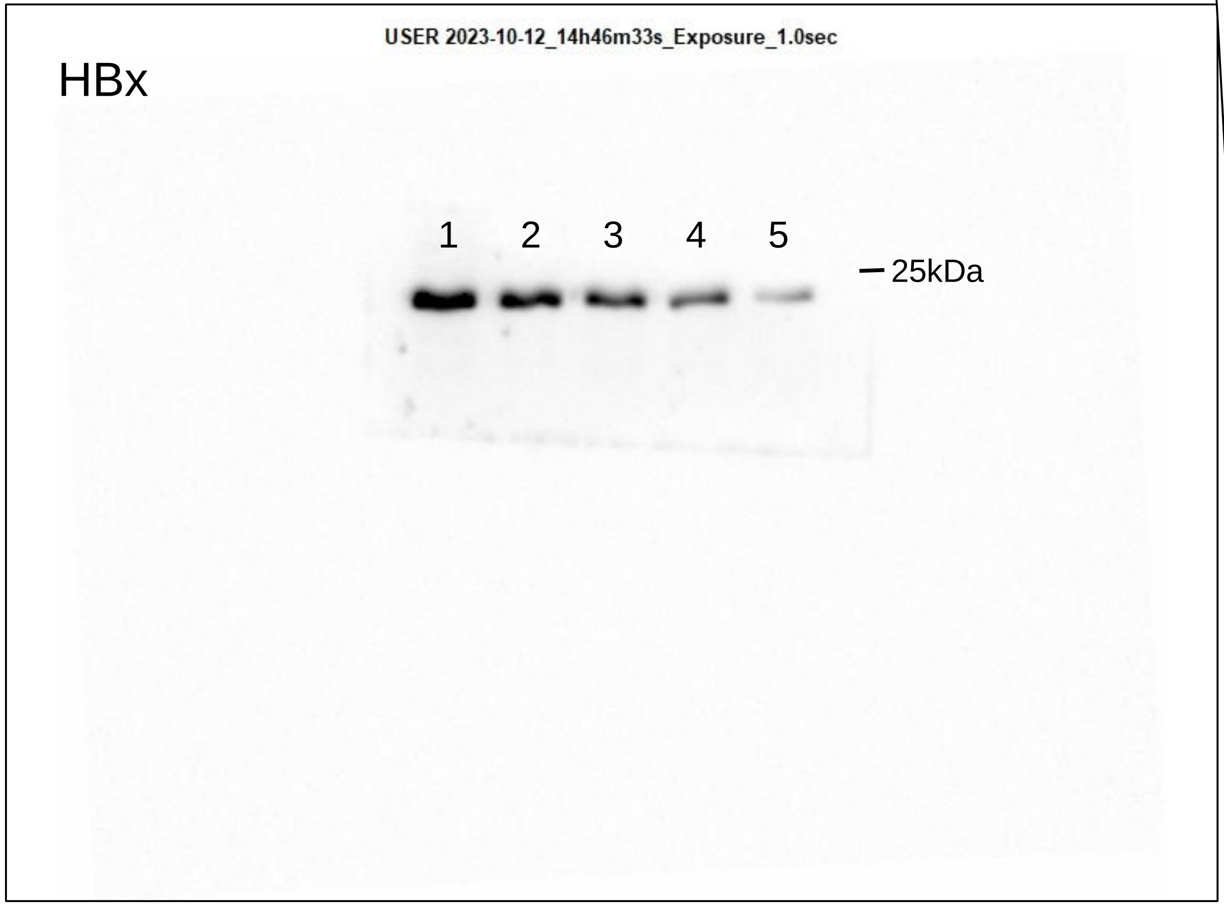

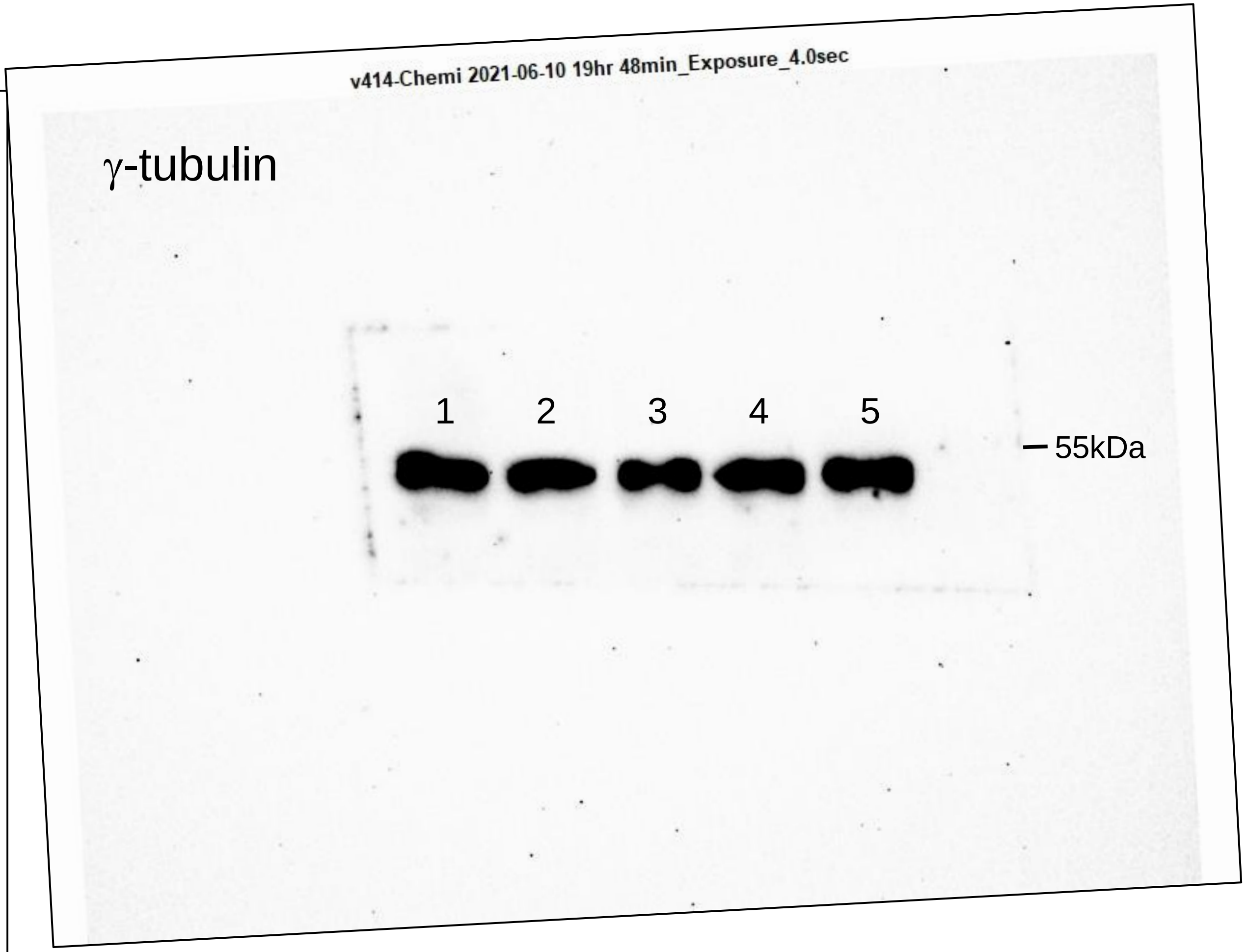

Fig. 5a

Lanes	1	2	3	4	5
CHX (min)	0	15	30	60	120

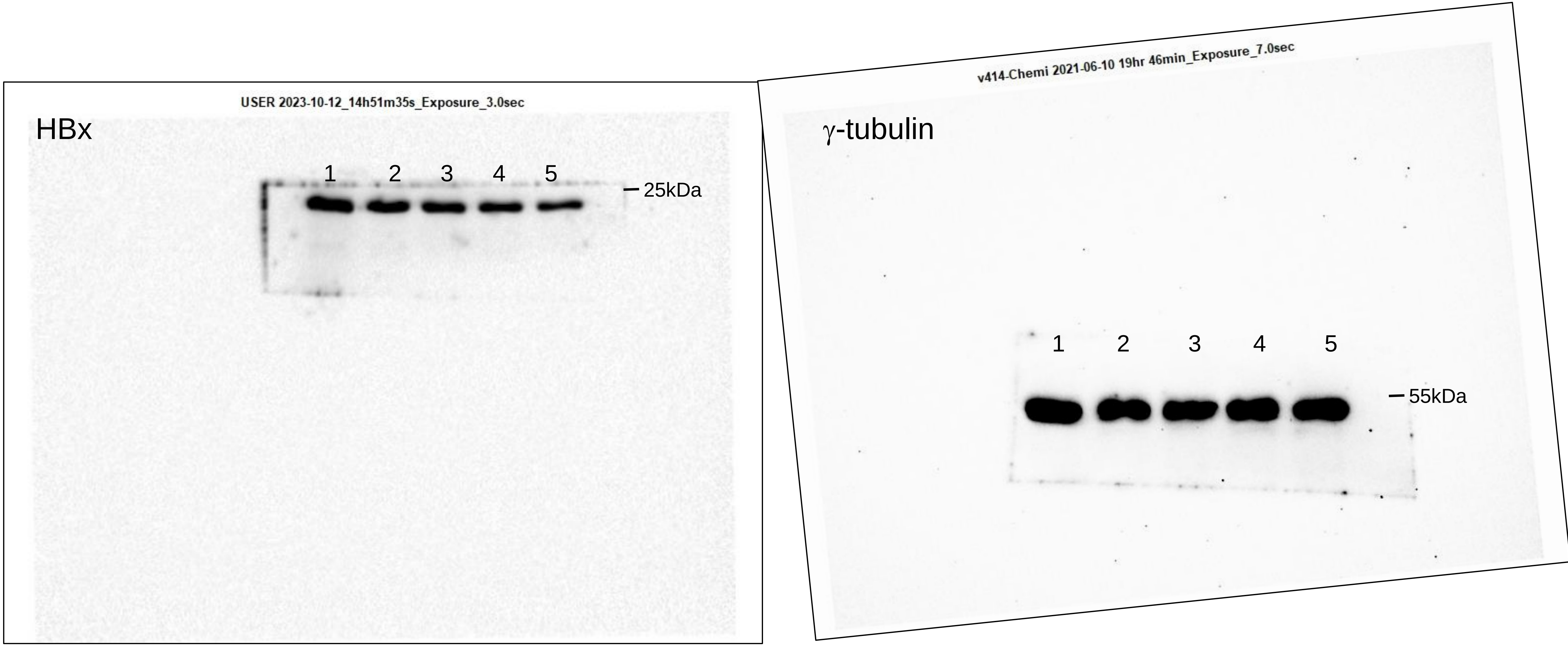

Fig. 5a

Lanes	1	2	3	4	5
CHX (min)	0	15	30	60	120

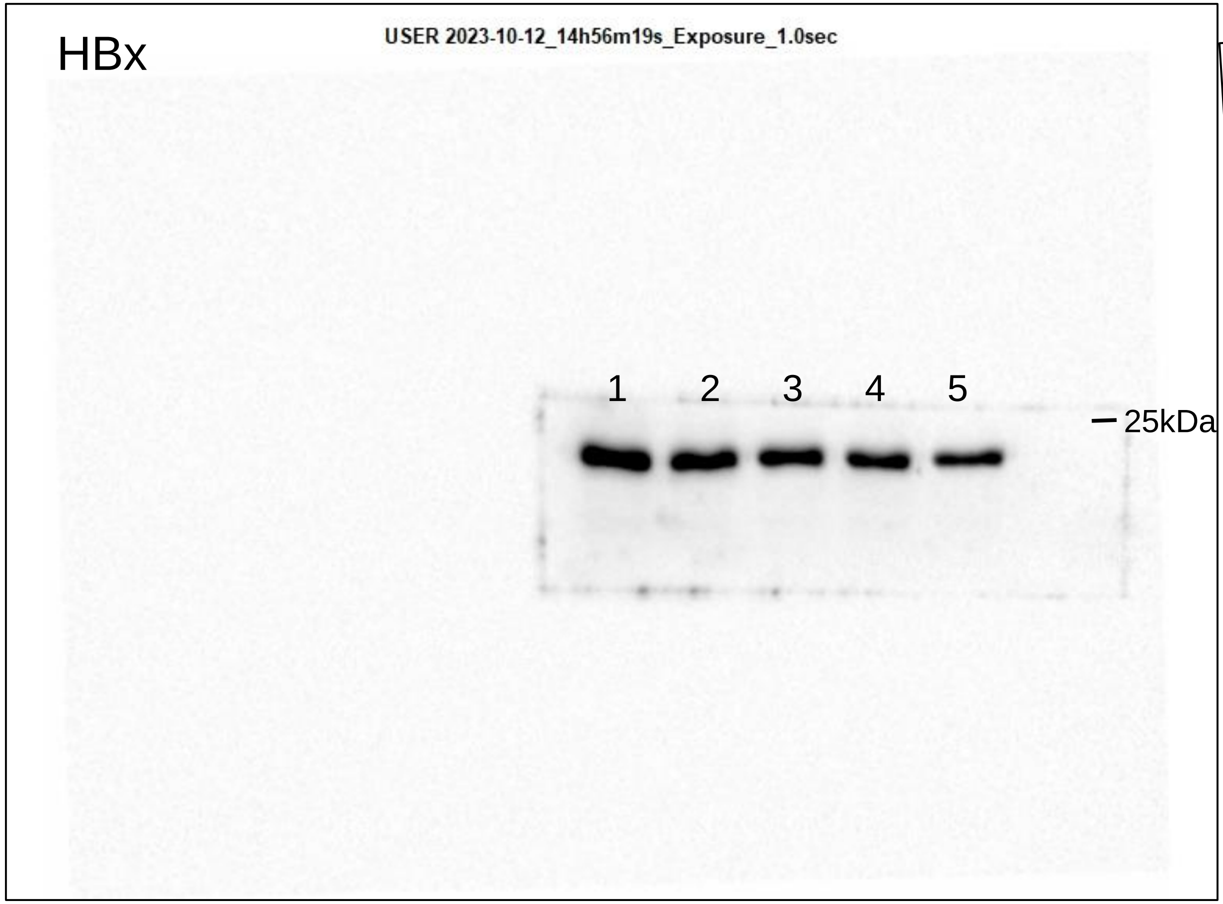

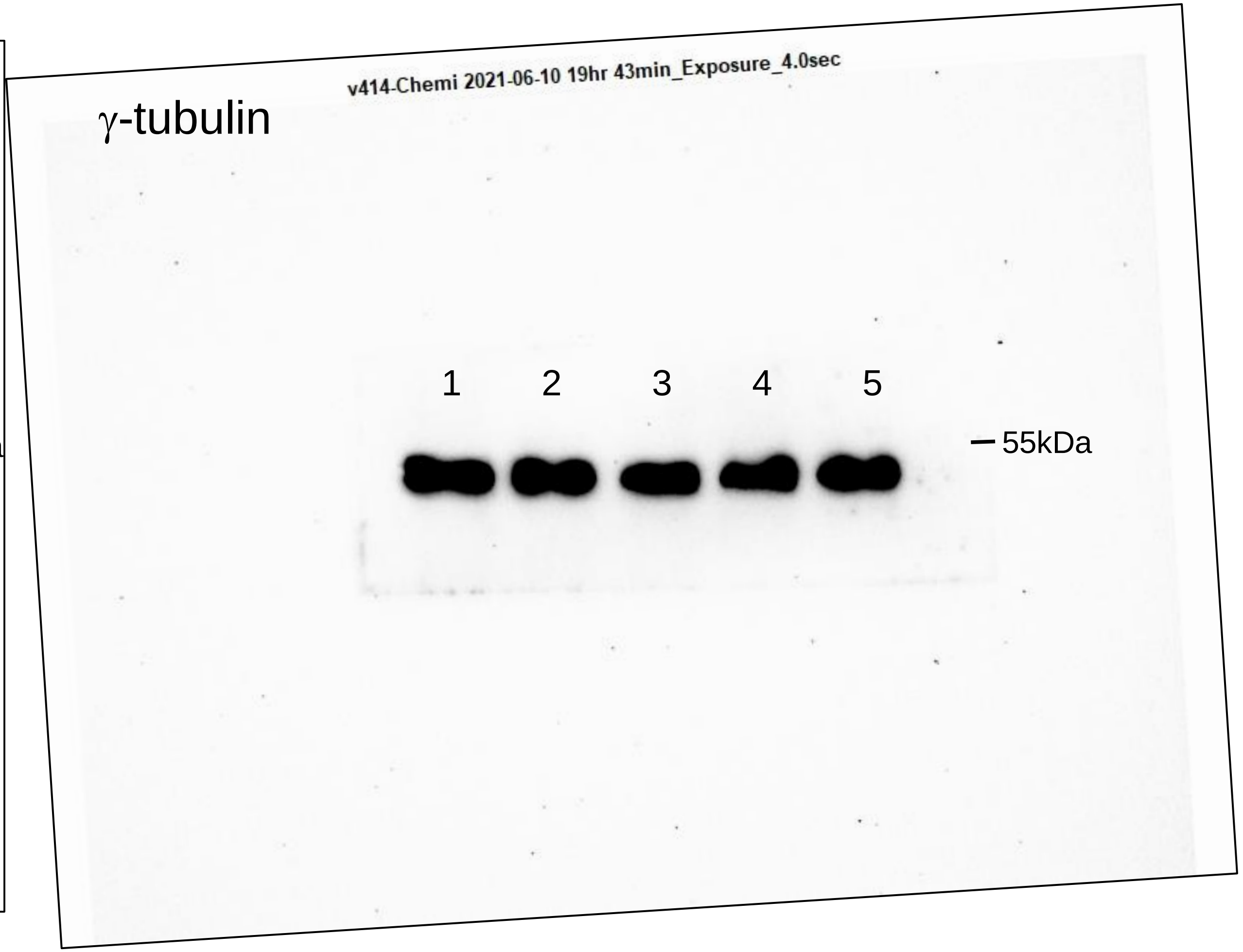

Fig. 5b IP : HBx

Lanes	1	2	3	4
HBx (μg)	0	0.5	0.5	0.5
ATRA (μM)	0	0	5	5
E6AP shRNA (μg)	0	0	0	0.5
HA-Ub (μg)	1.0	1.0	1.0	1.0

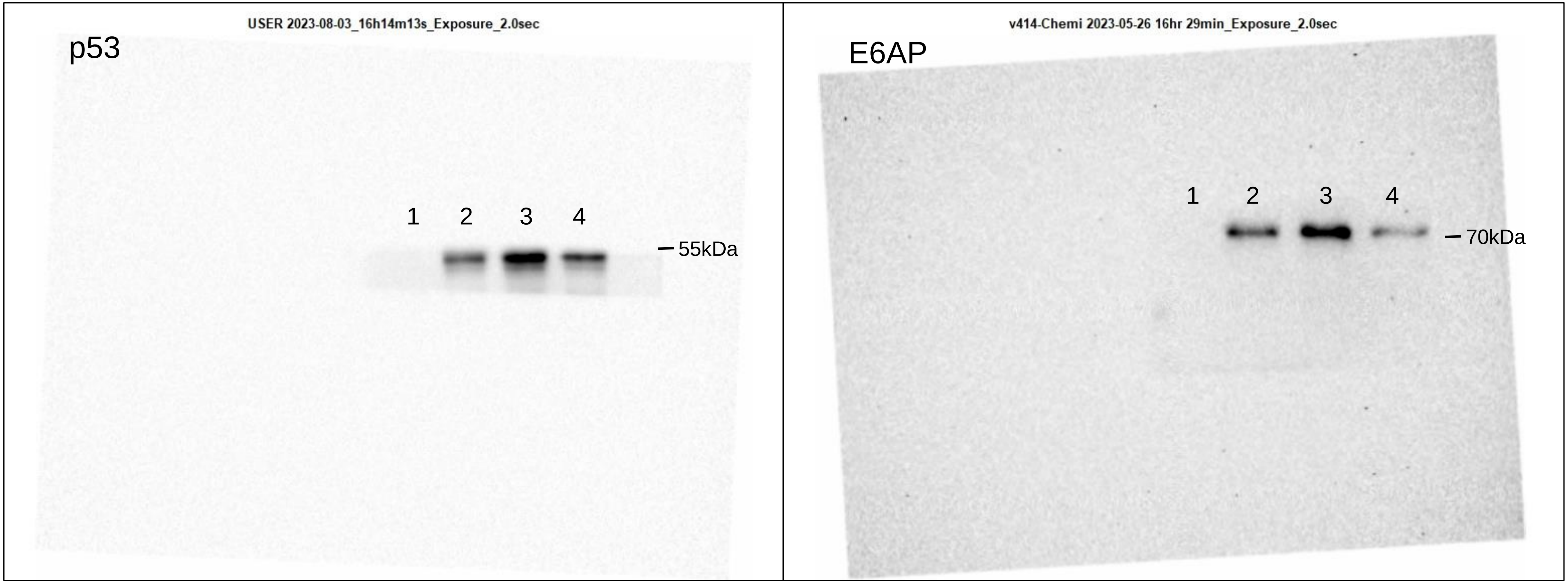

Fig. 5b IP : HBx

Lanes	1	2	3	4
HBx (μg)	0	0.5	0.5	0.5
ATRA (μM)	0	0	5	5
E6AP shRNA (μg)	0	0	0	0.5
HA-Ub (μg)	1.0	1.0	1.0	1.0

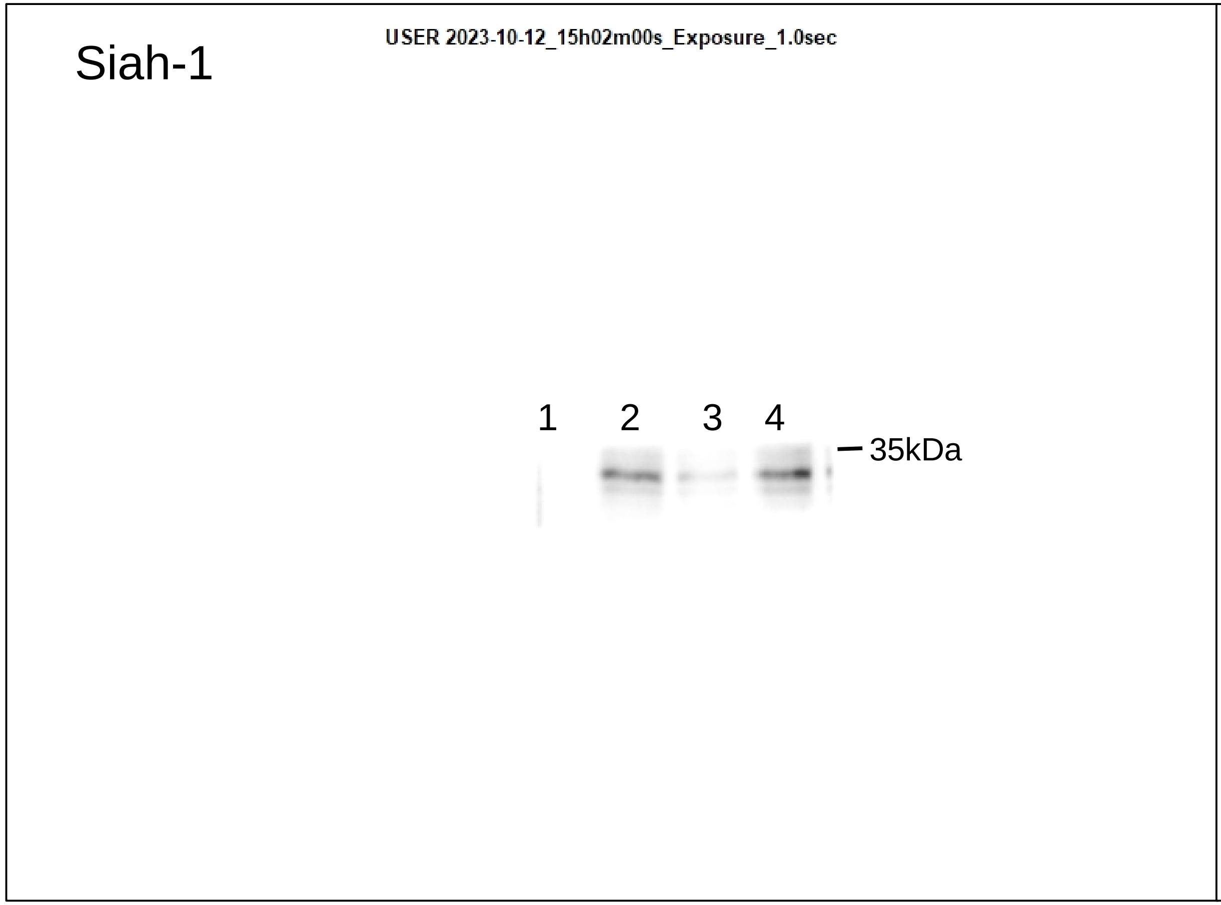

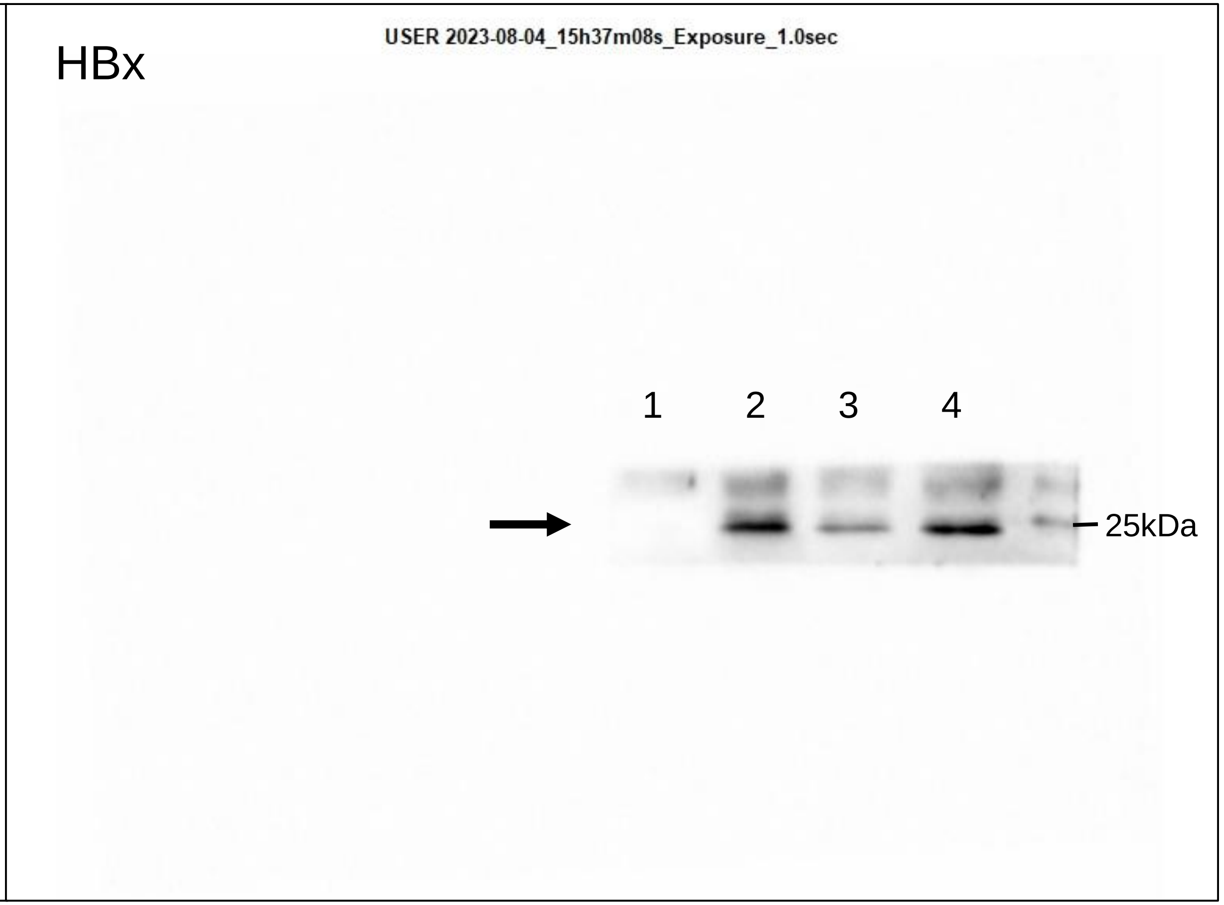

Fig. 5b IP : HBx

Lanes	1	2	3	4
HBx (μg)	0	0.5	0.5	0.5
ATRA (μM)	0	0	5	5
E6AP shRNA (μg)	0	0	0	0.5
HA-Ub (μg)	1.0	1.0	1.0	1.0

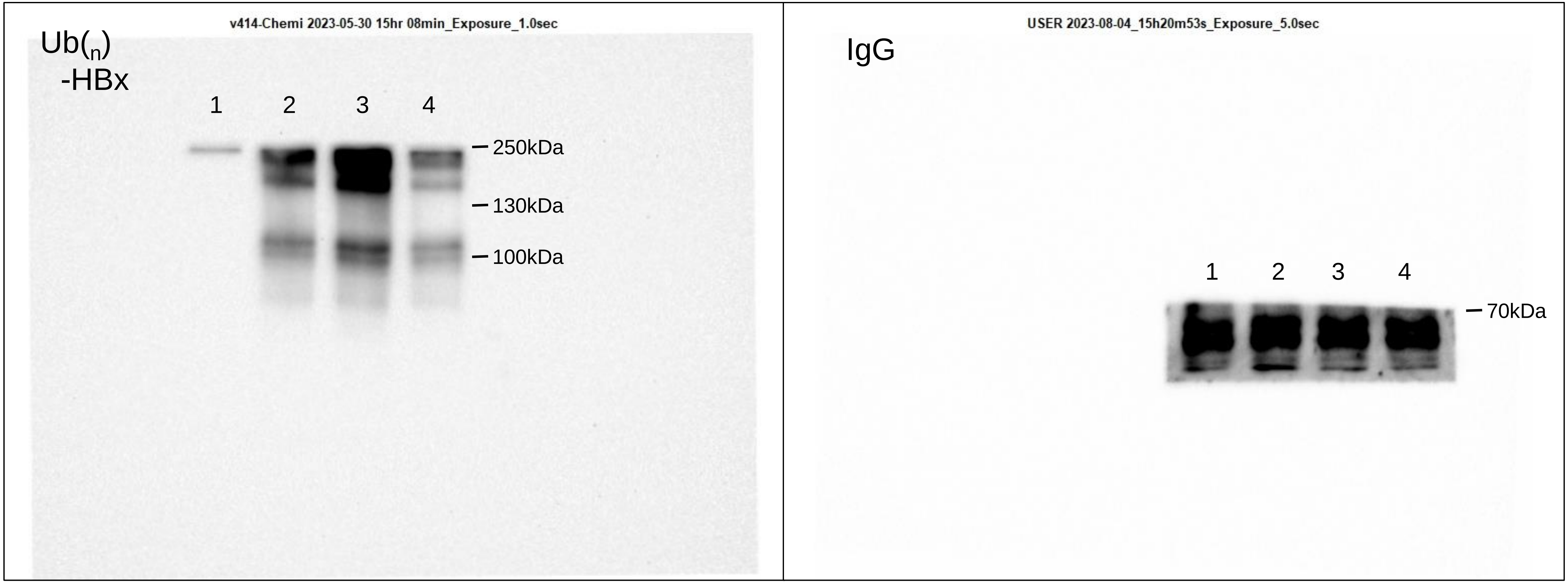

Fig. 5b input

Lanes	1	2	3	4
HBx (μg)	0	0.5	0.5	0.5
ATRA (μM)	0	0	5	5
E6AP shRNA (μg)	0	0	0	0.5
HA-Ub (μg)	1.0	1.0	1.0	1.0

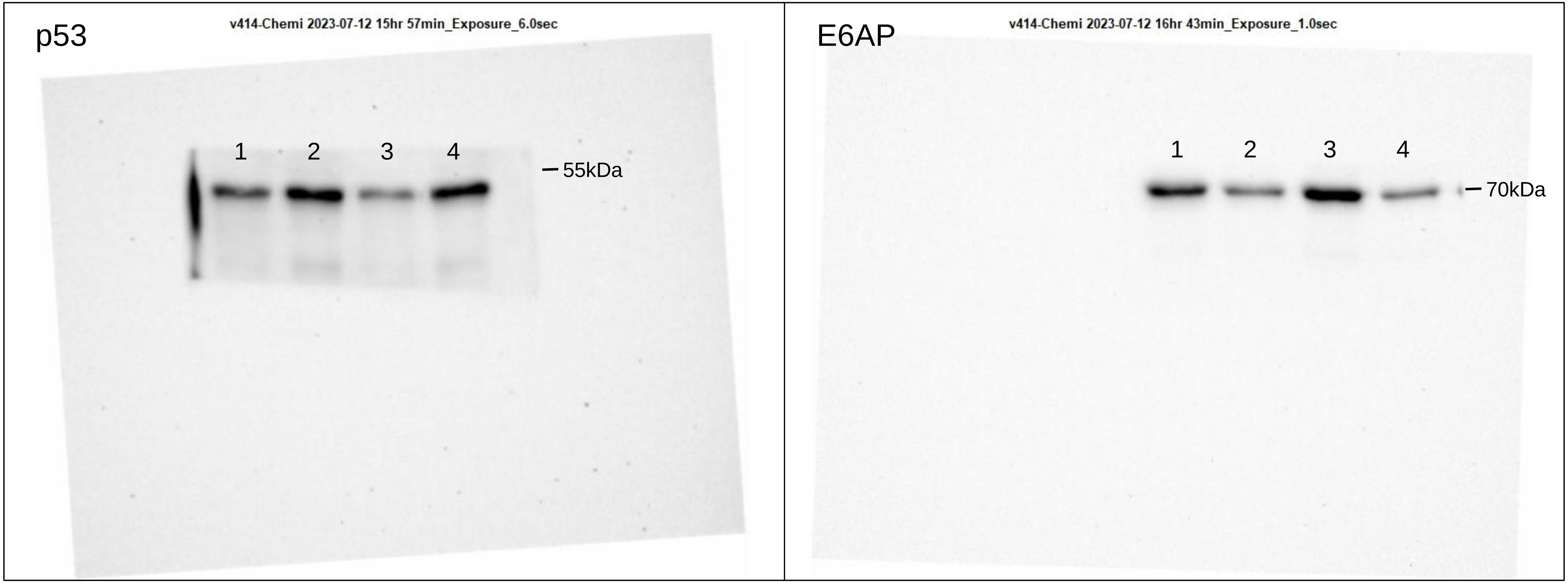

Fig. 5b input

Lanes	1	2	3	4
HBx (μg)	0	0.5	0.5	0.5
ATRA (μM)	0	0	5	5
E6AP shRNA (μg)	0	0	0	0.5
HA-Ub (μg)	1.0	1.0	1.0	1.0

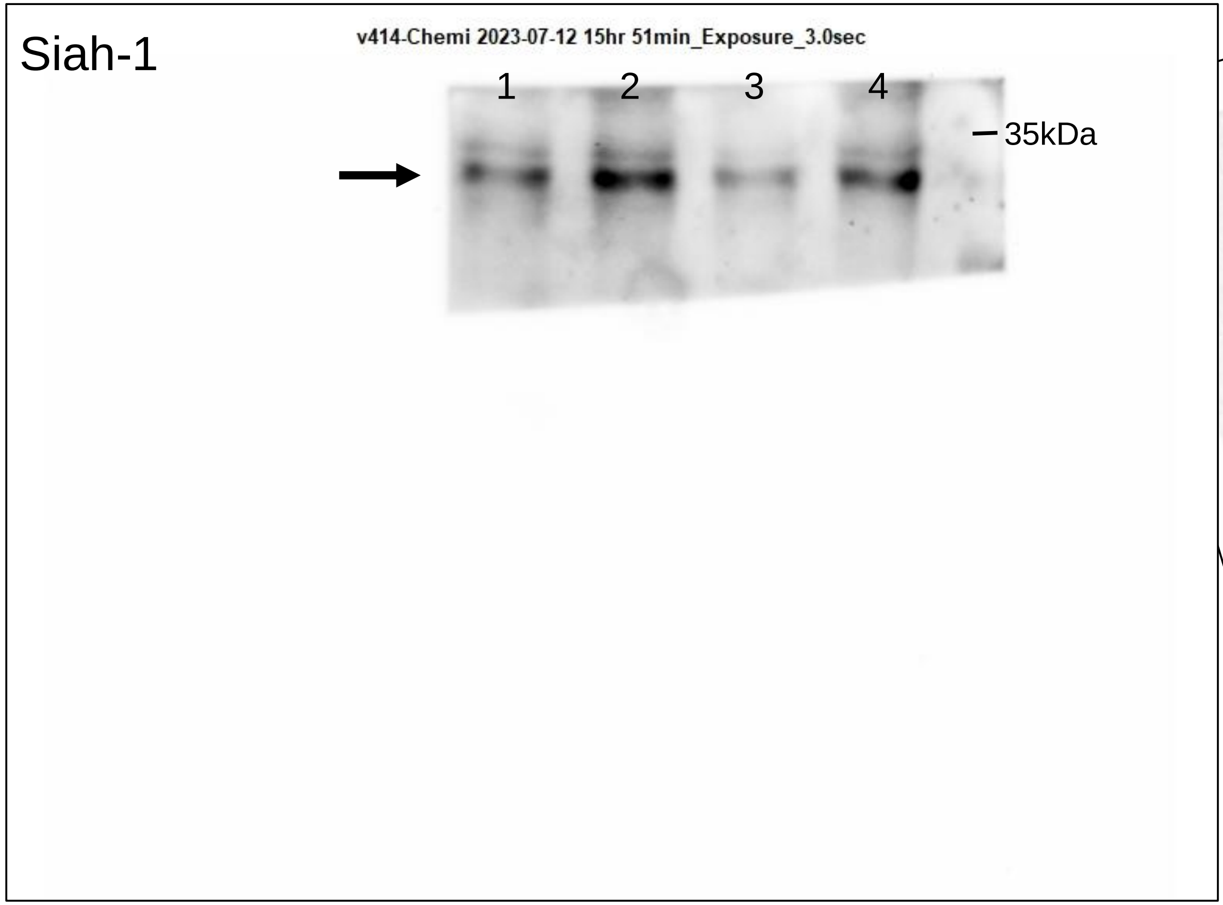

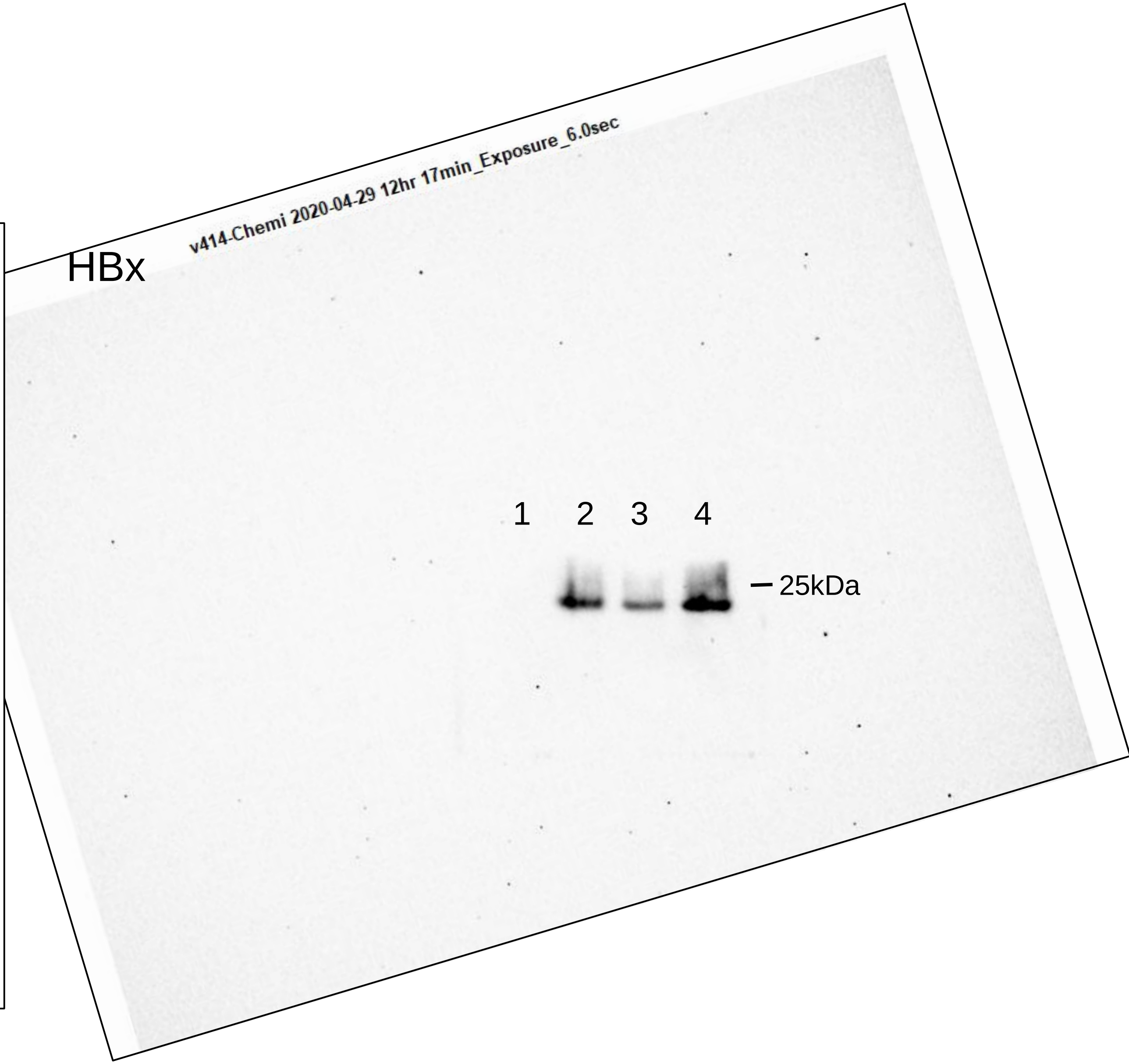

Fig. 5b input

Lanes	1	2	3	4
HBx (μg)	0	0.5	0.5	0.5
ATRA (μM)	0	0	5	5
E6AP shRNA (μg)	0	0	0	0.5
HA-Ub (μg)	1.0	1.0	1.0	1.0

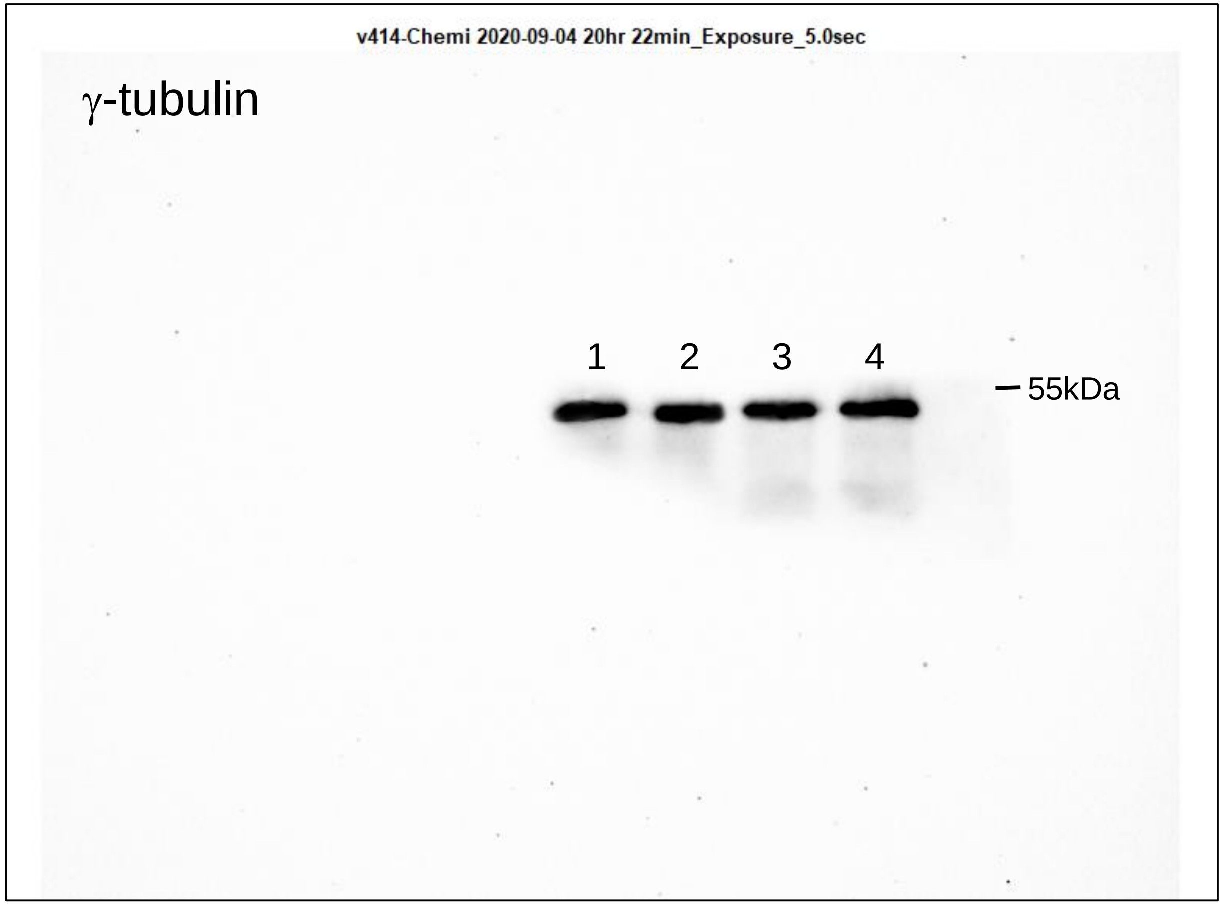

Fig. 5c

Lanes	1	2	3	4	5	6
HBx (μg)	0	0.5	0.5	0	0.5	0.5
ATRA (μM)	0	0	5	0	0	5
MG132 (μM)	0	0	0	10	10	10

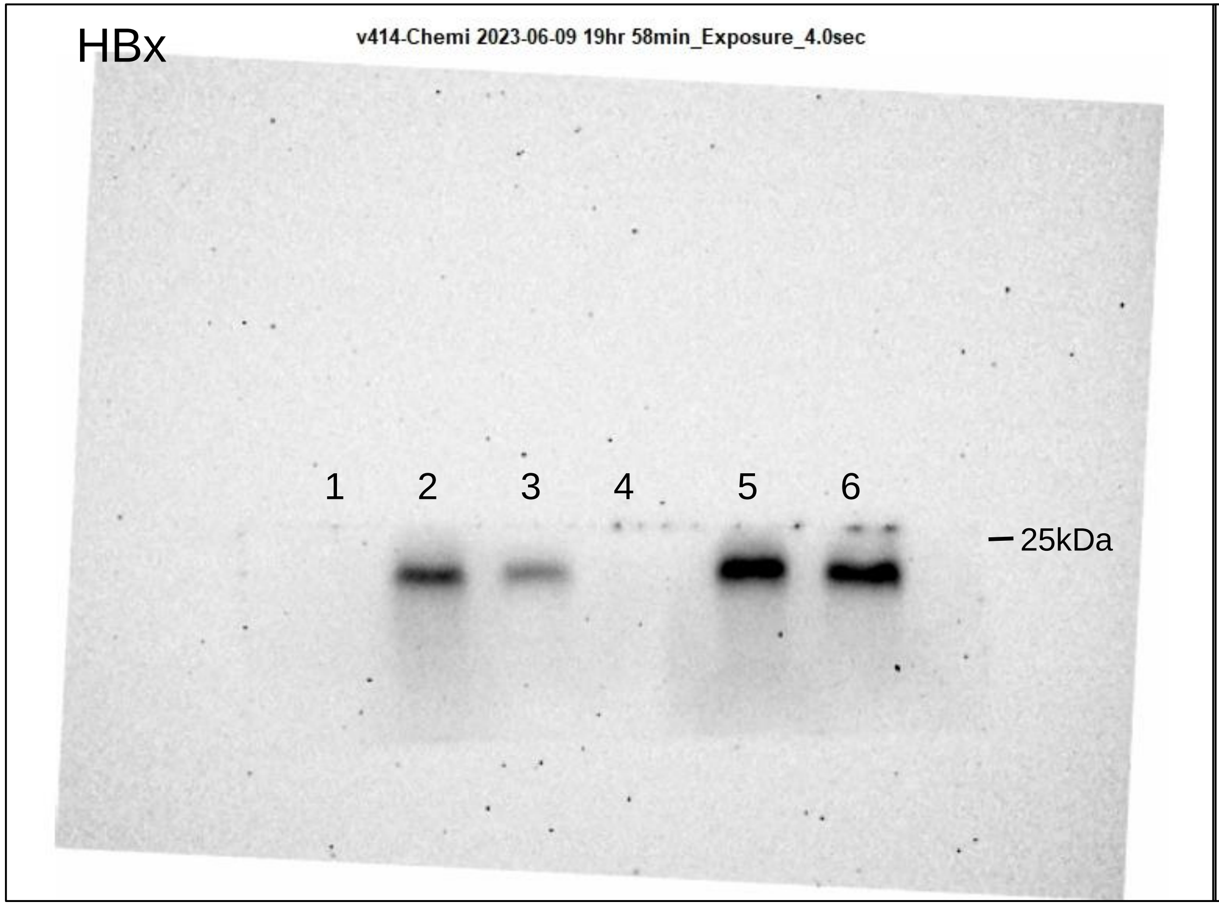

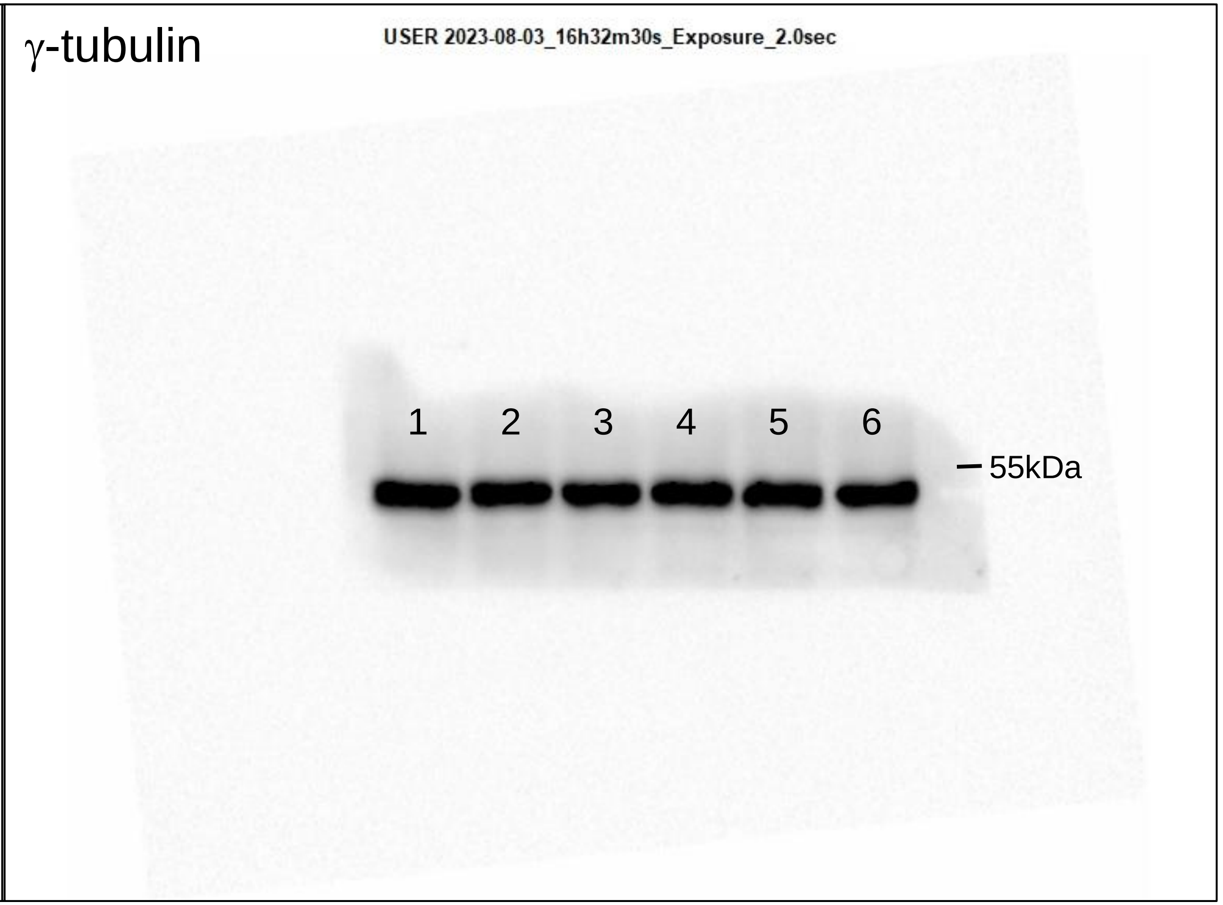

Fig. 6a

Lanes	1	2	3	4	5
CHX (min)	0	15	30	60	120

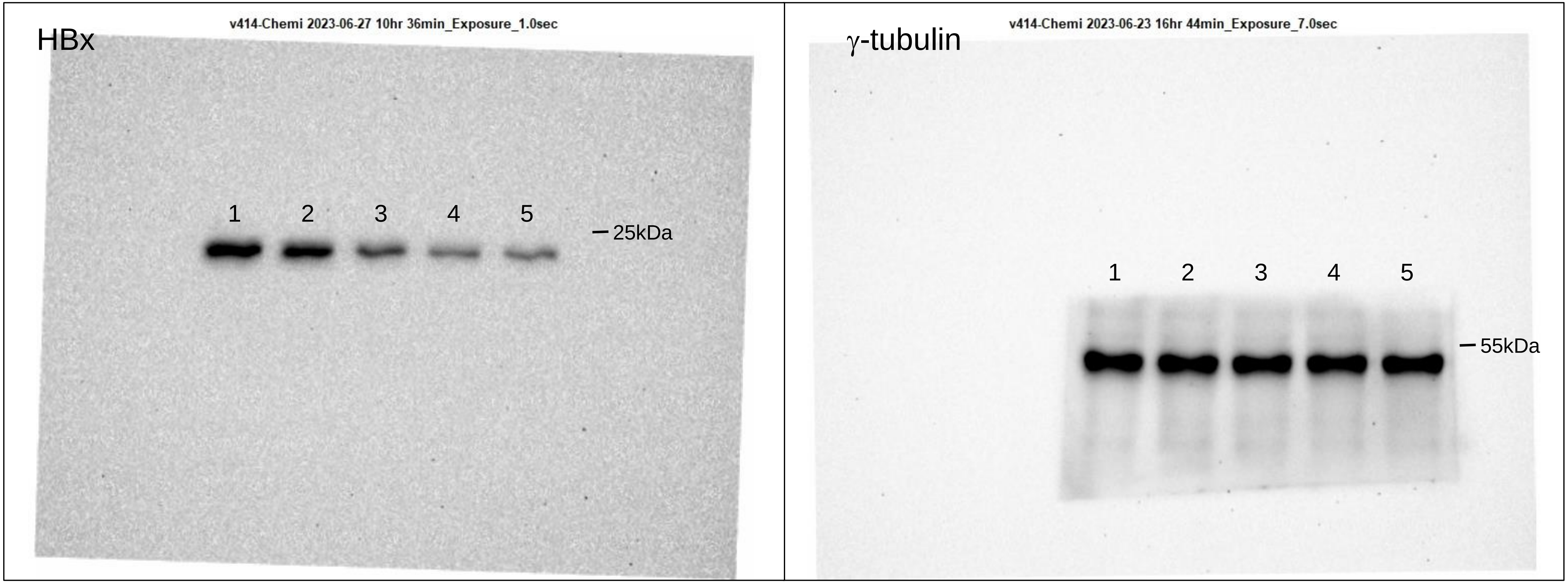

Fig. 6a

Lanes	1	2	3	4	5
CHX (min)	0	15	30	60	120

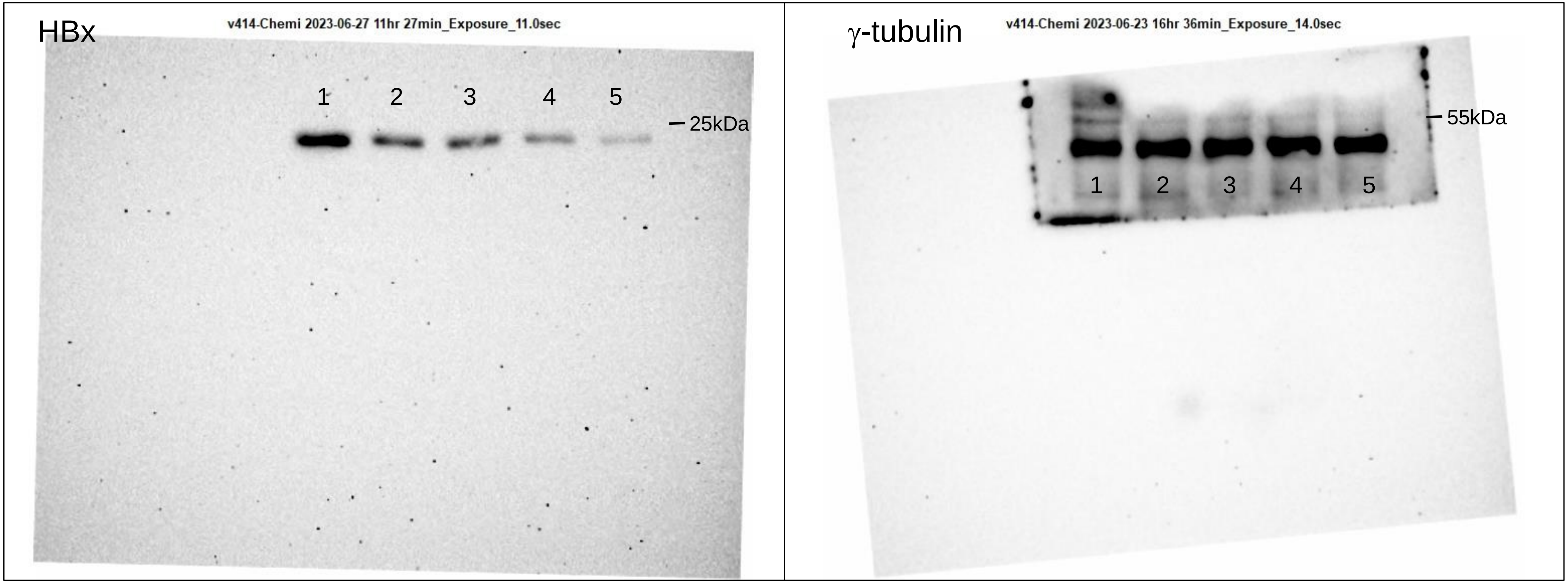

Fig. 6a

Lanes	1	2	3	4	5
CHX (min)	0	15	30	60	120

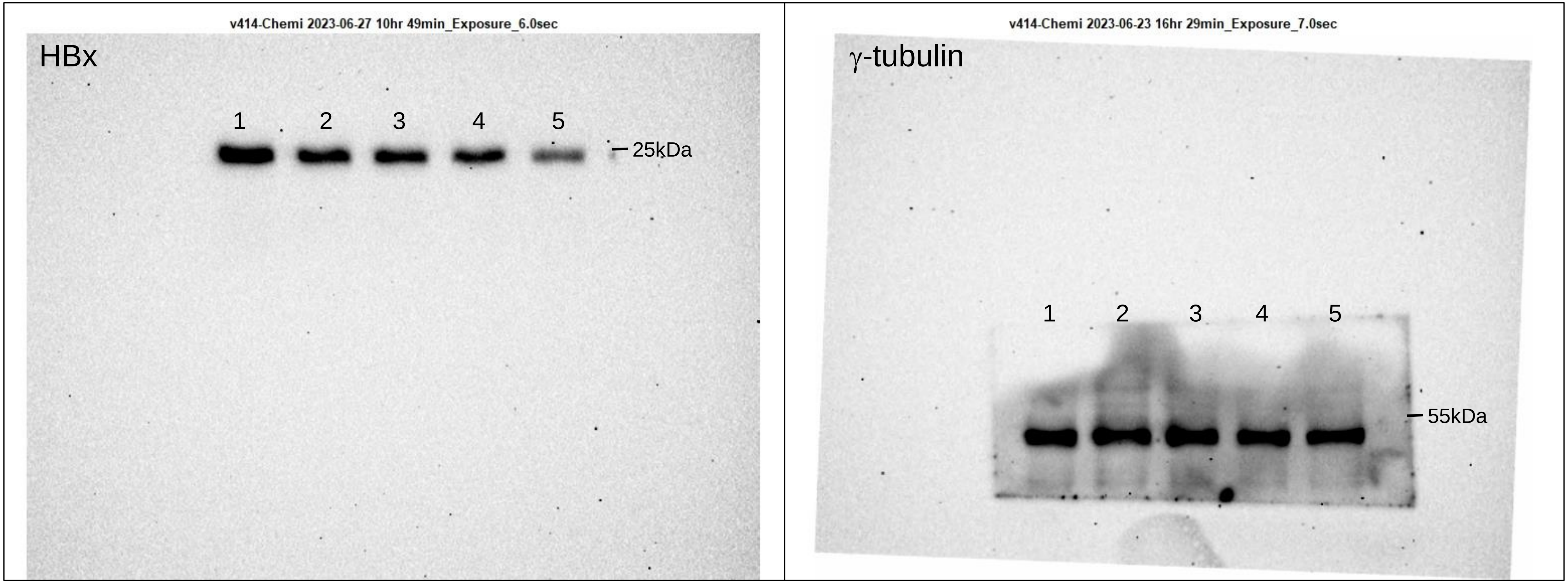

Fig. 6a

Lanes	1	2	3	4	5
CHX (min)	0	15	30	60	120

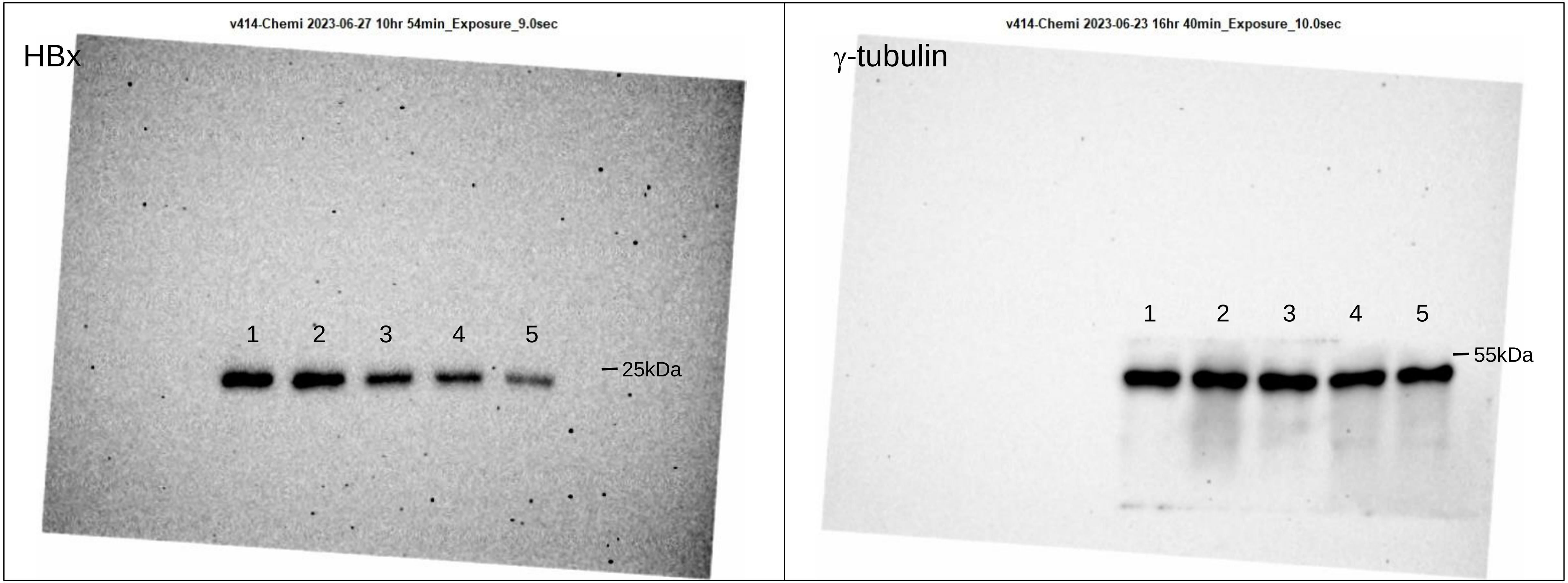

Fig. 6b IP:HBx

Lanes	1	2	3	4
HBV (MOI)	0	50	50	50
ATRA (μM)	0	0	5	5
E6AP shRNA (μg)	0	0	0	0.5
HA-Ub (μg)	1.0	1.0	1.0	1.0

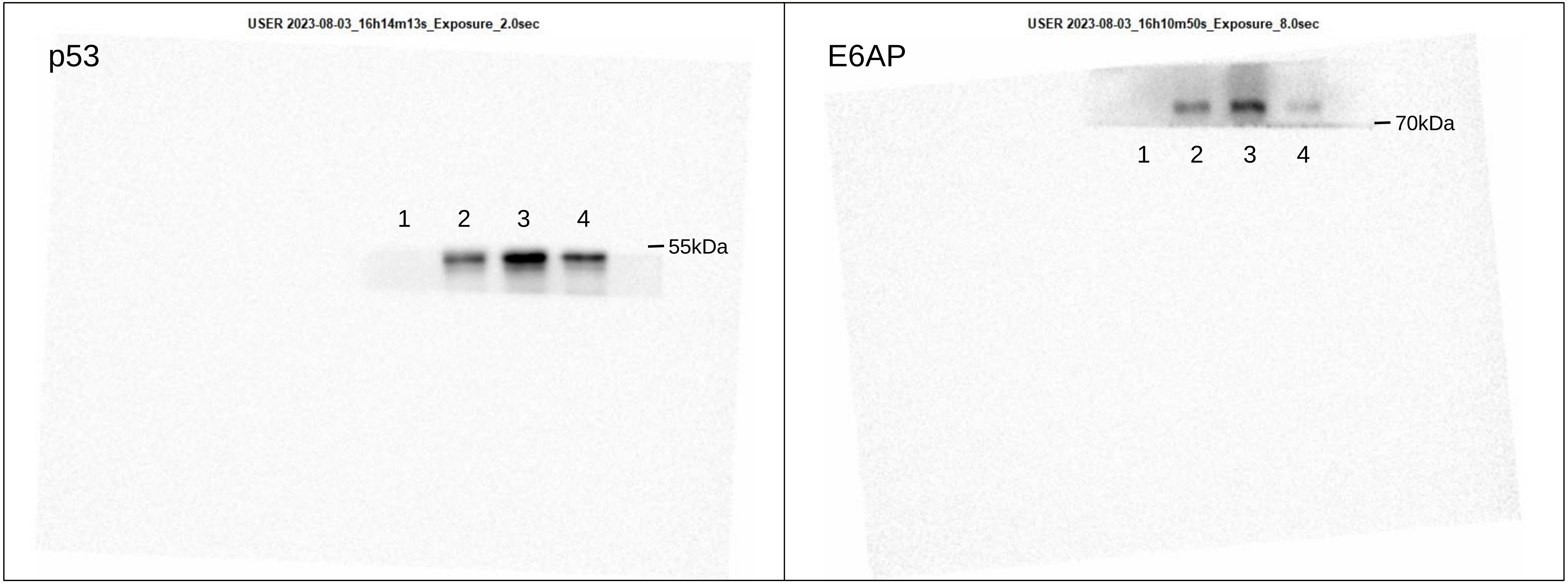

Fig. 6b IP:HBx

Lanes	1	2	3	4
HBV (MOI)	0	50	50	50
ATRA (μM)	0	0	5	5
E6AP shRNA (μg)	0	0	0	0.5
HA-Ub (μg)	1.0	1.0	1.0	1.0

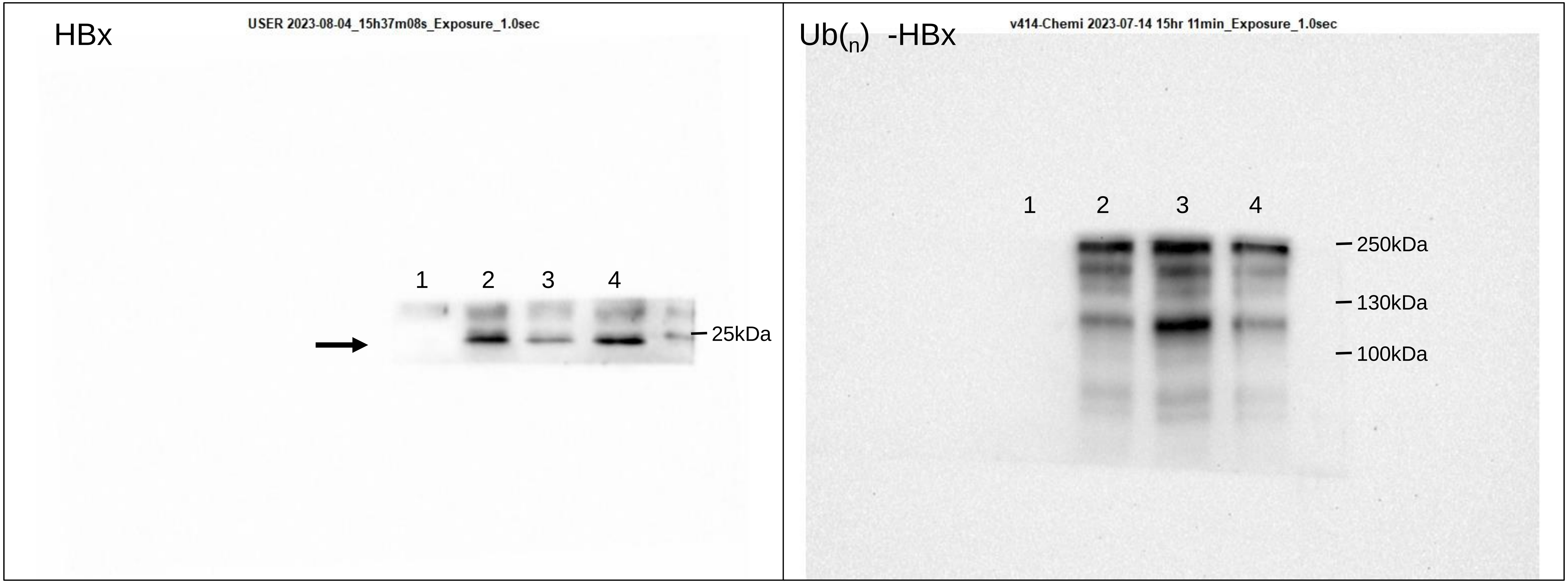

Fig. 6b IP:HBx

Lanes	1	2	3	4
HBV (MOI)	0	50	50	50
ATRA (μM)	0	0	5	5
E6AP shRNA (μg)	0	0	0	0.5
HA-Ub (μg)	1.0	1.0	1.0	1.0

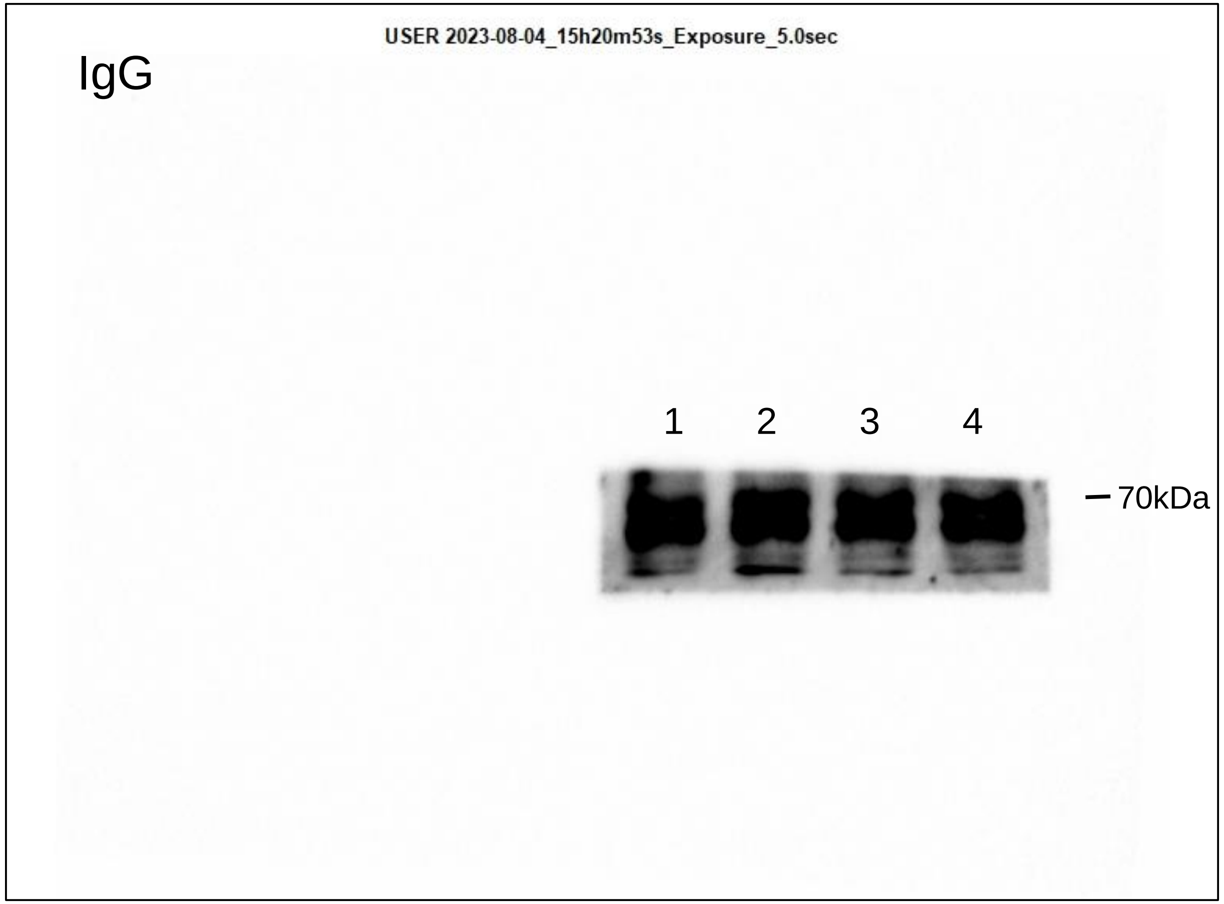

Fig. 6b input

Lanes	1	2	3	4
HBV (MOI)	0	50	50	50
ATRA (μM)	0	0	5	5
E6AP shRNA (μg)	0	0	0	0.5
HA-Ub (μg)	1.0	1.0	1.0	1.0

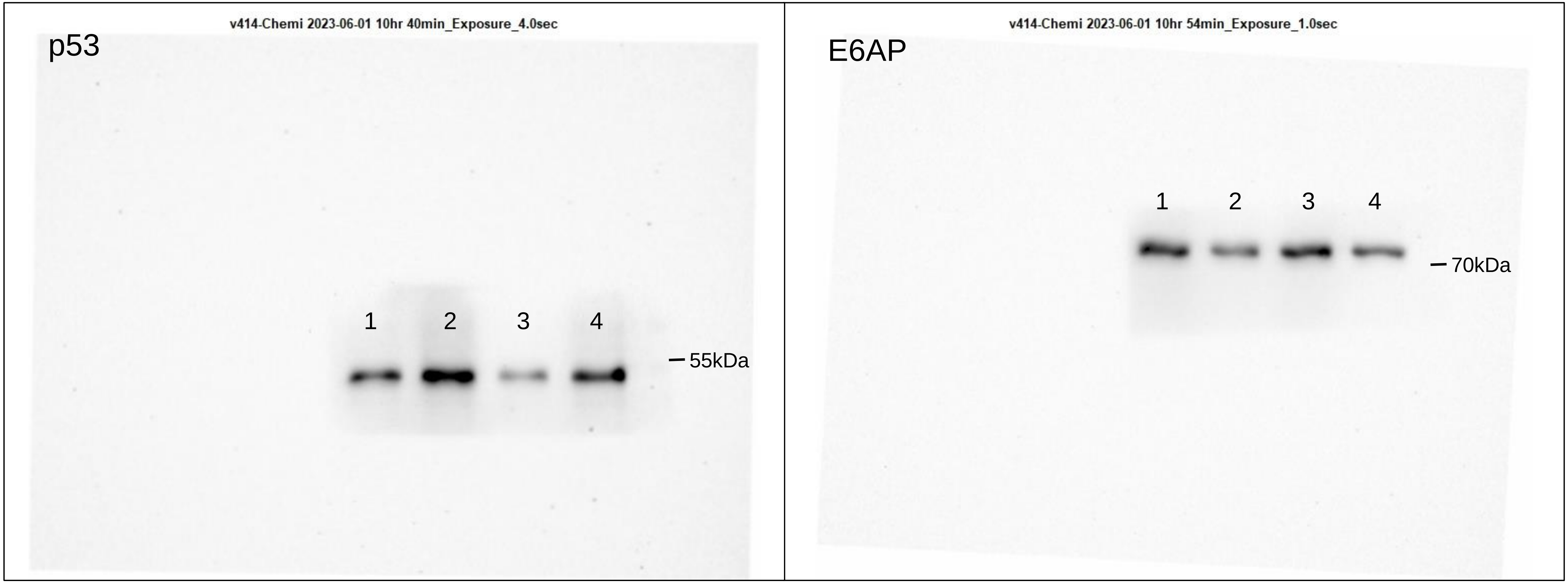

Fig. 6b input

Lanes	1	2	3	4
HBV (MOI)	0	50	50	50
ATRA (μM)	0	0	5	5
E6AP shRNA (μg)	0	0	0	0.5
HA-Ub (μg)	1.0	1.0	1.0	1.0

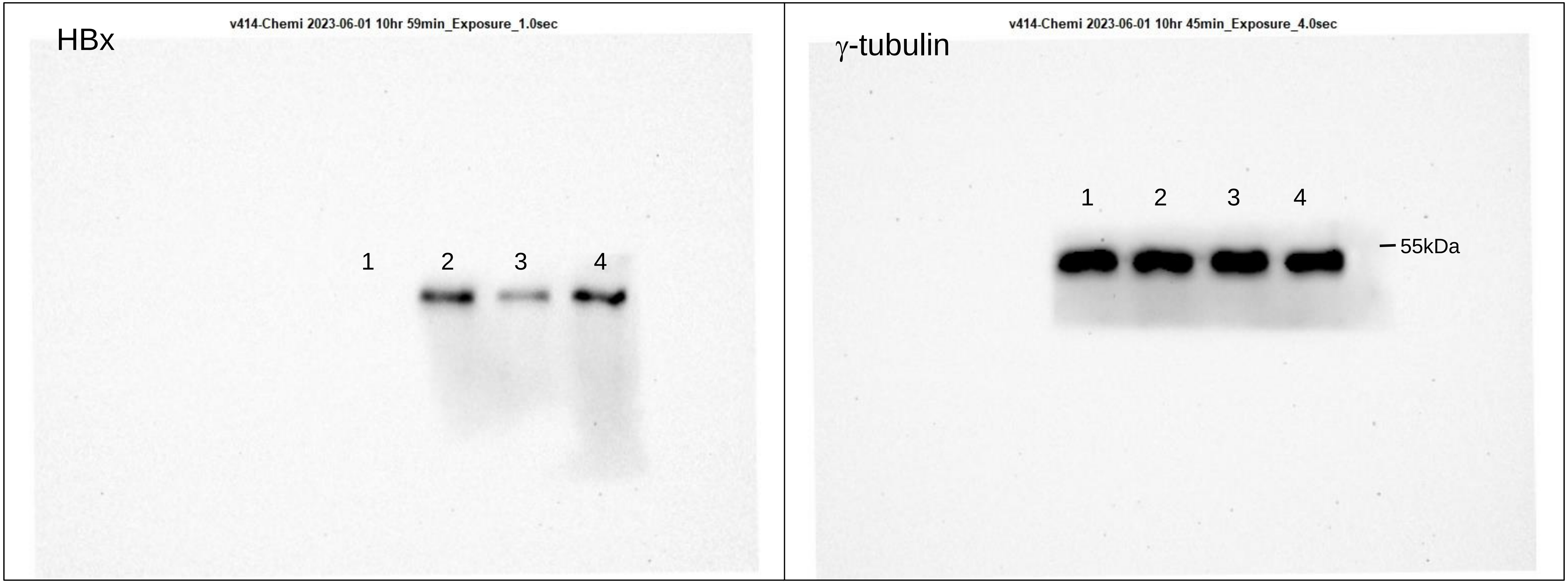

Fig. 6c

HBV (MOI)	50	50	50	50
ATRA (μM)	0	5	5	5
SC shRNA (μg)	1	1	0.9	0
E6AP shRNA (μg)	0	0	0.1	1.0

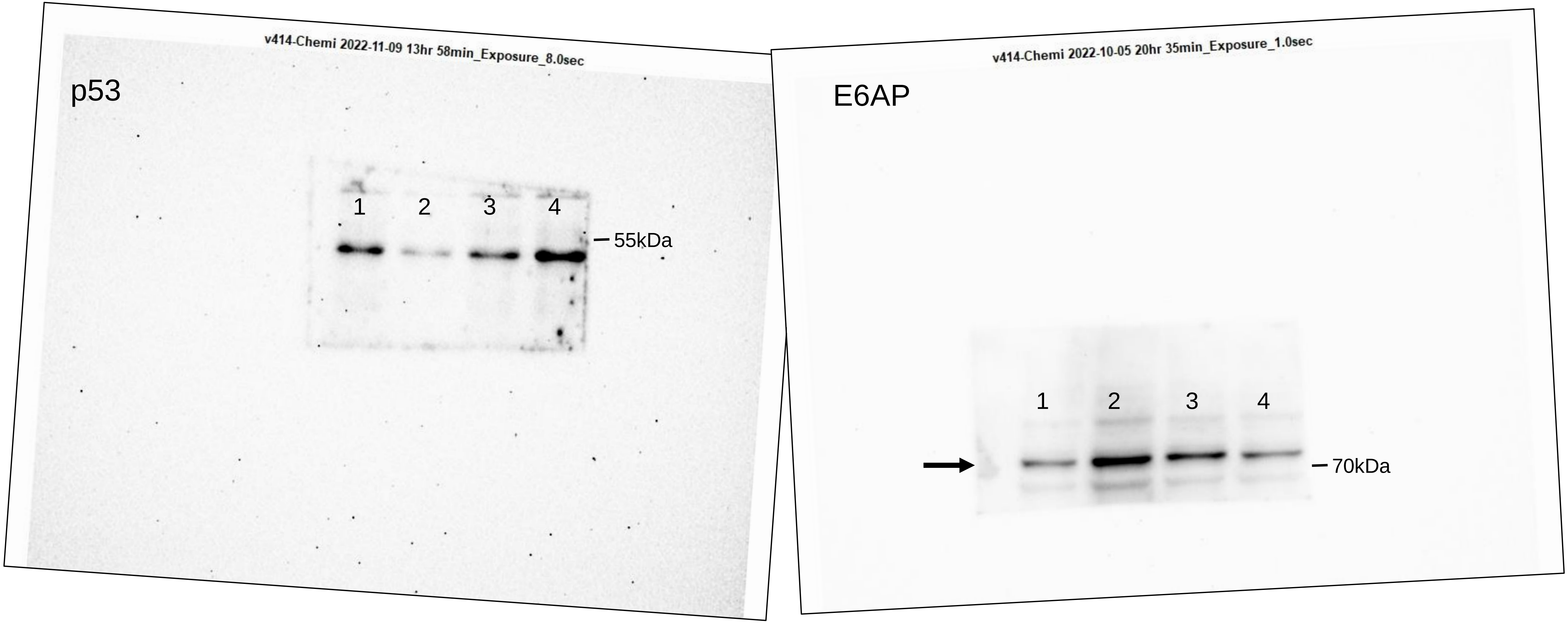

Fig. 6c

HBV (MOI)	50	50	50	50
ATRA (μM)	0	5	5	5
SC shRNA (μg)	1	1	0.9	0
E6AP shRNA (μg)	0	0	0.1	1.0

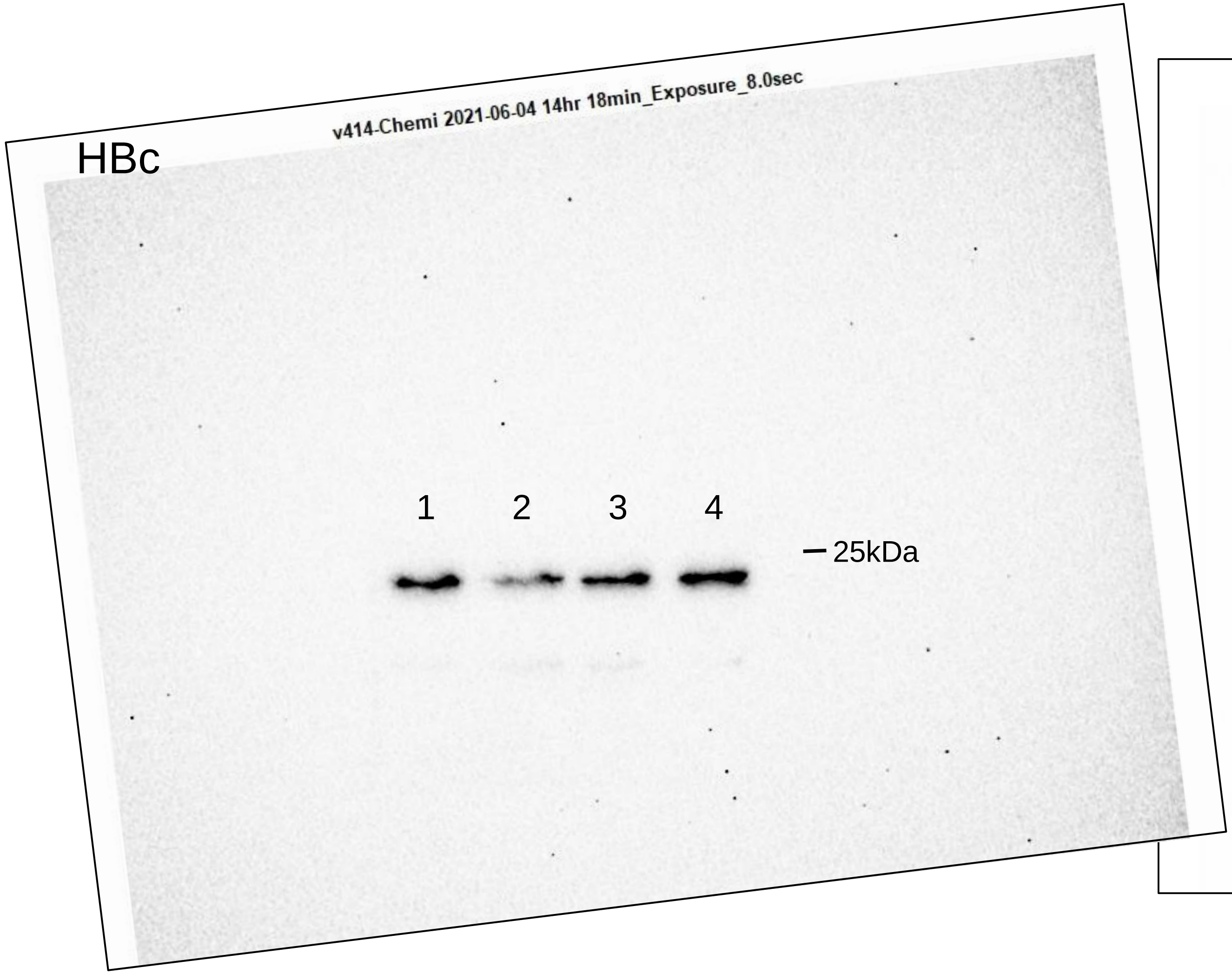

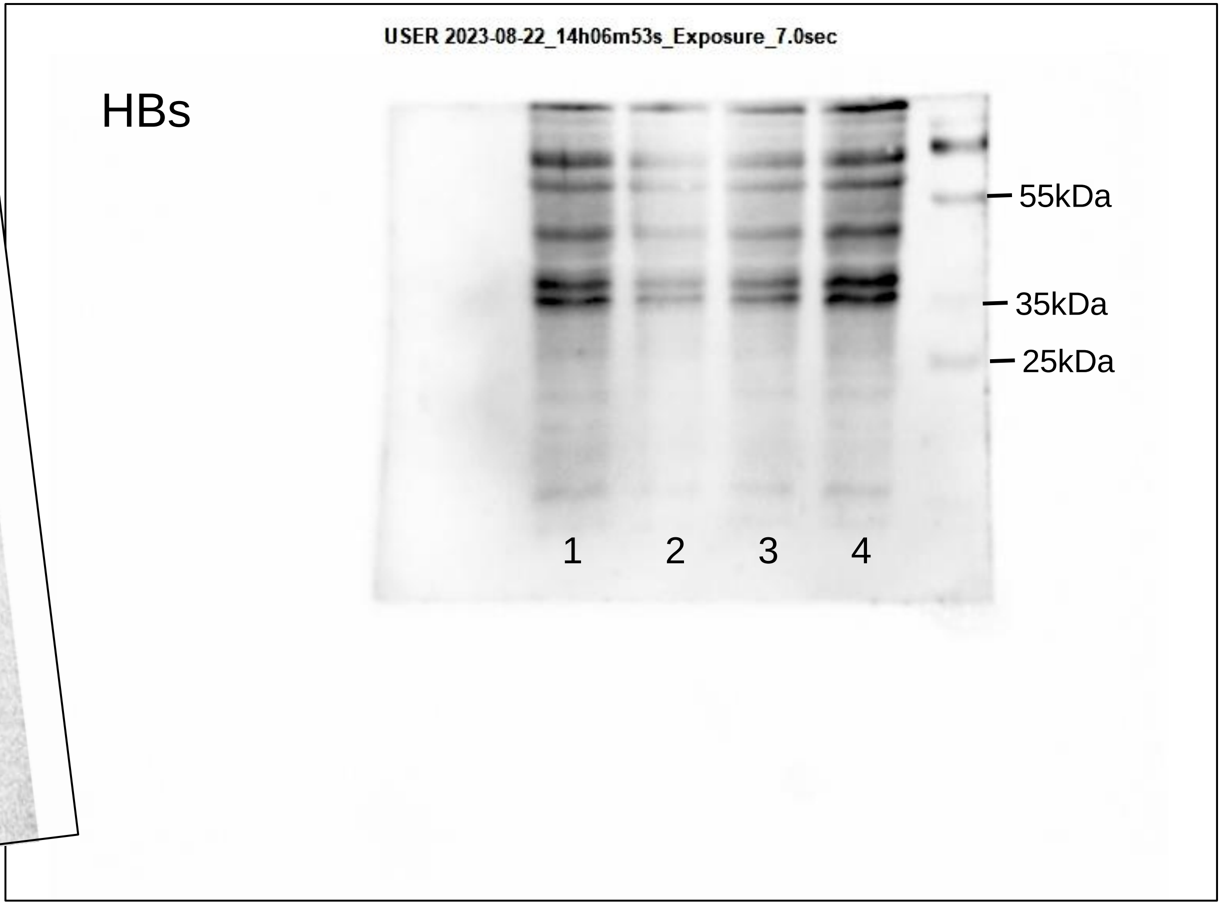

Fig. 6c

HBV (MOI)	50	50	50	50
ATRA (μM)	0	5	5	5
SC shRNA (μg)	1	1	0.9	0
E6AP shRNA (μg)	0	0	0.1	1.0

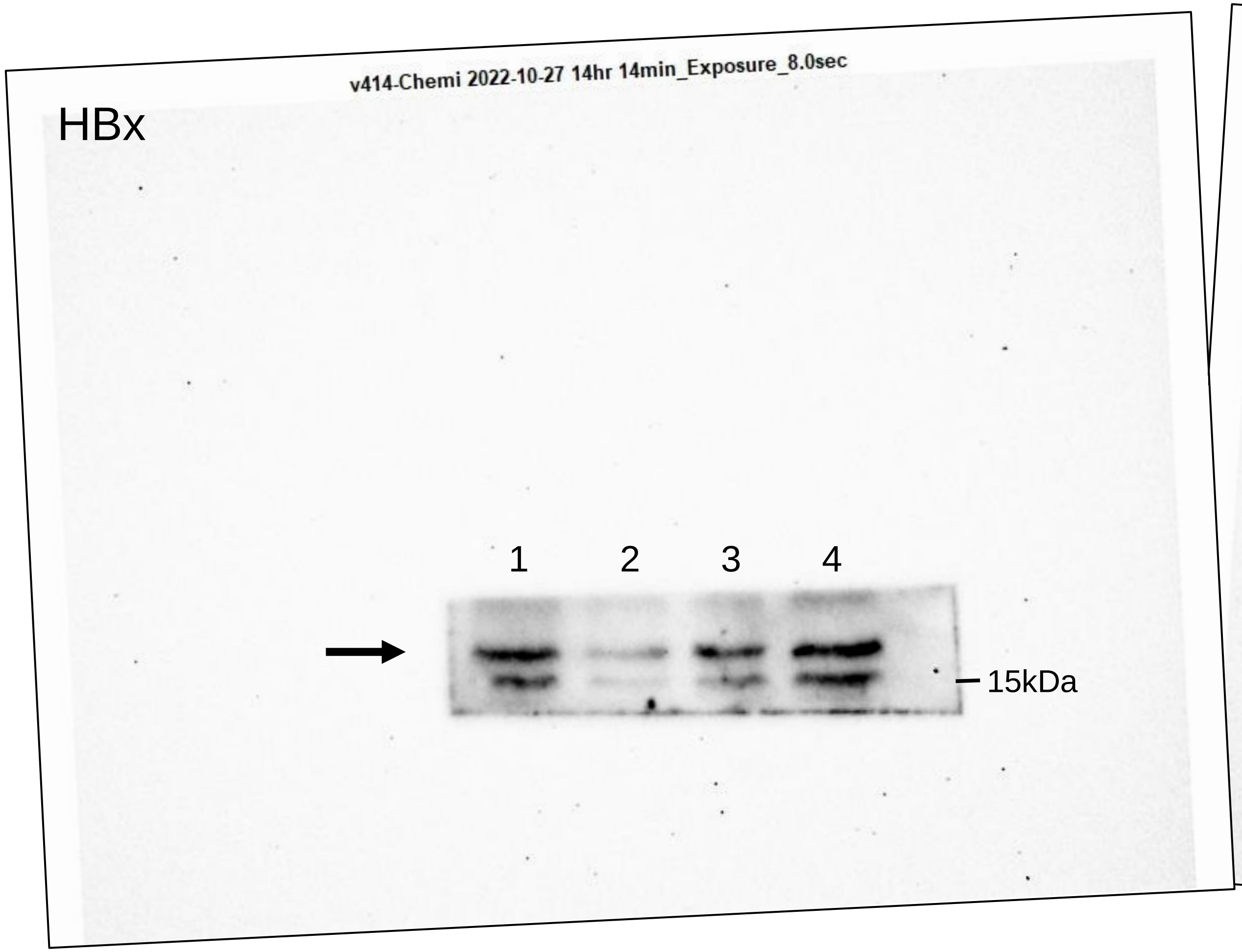

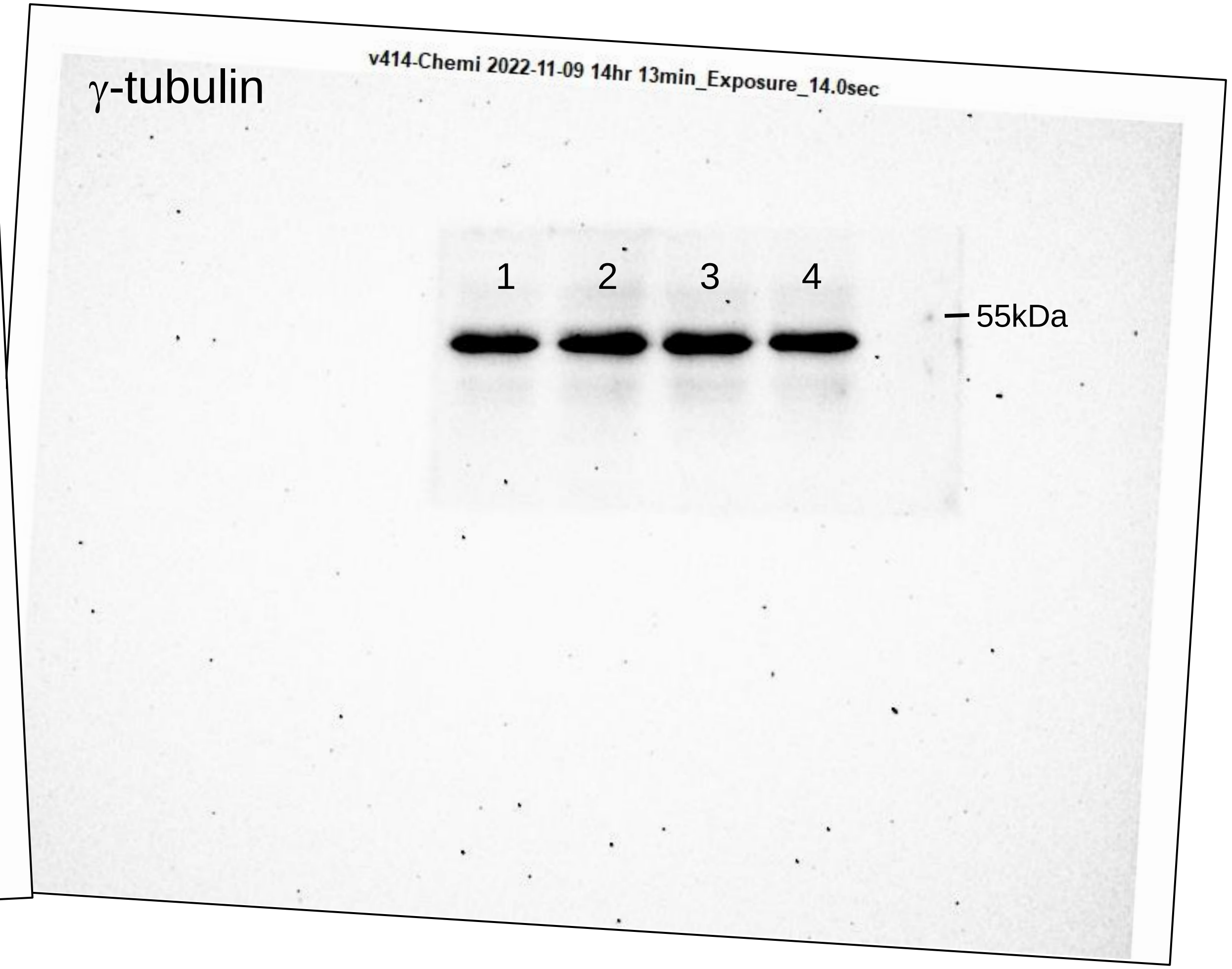

Fig. 6e

Lanes	1	2	3	4	5	6
HBV (MOI)	0	50	50	50	50	50
ATRA (μM)	0	0	1	1	1	1
SC shRNA (μg)	1.0	1.0	1.0	0	0	0
E6AP shRNA (μg)	0	0	0	1.0	0	0
E6AP (μg)	0	0	0	0	0	1.0

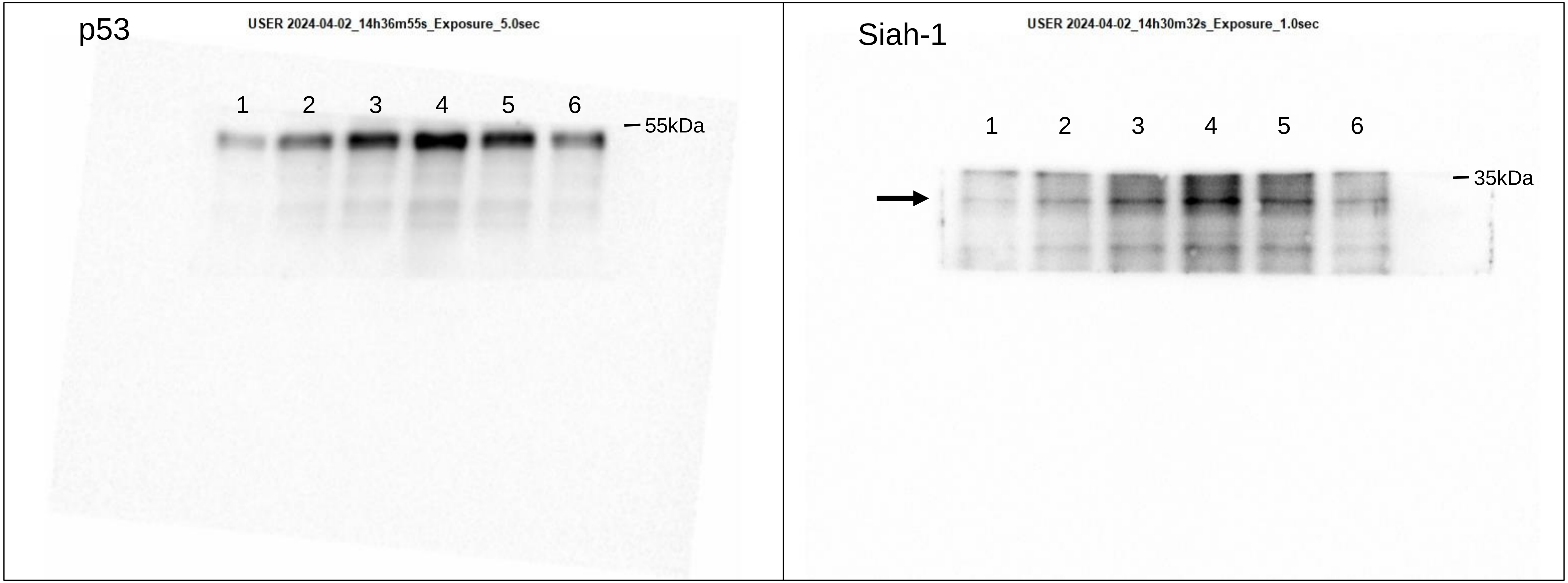

Fig. 6e

Lanes	1	2	3	4	5	6
HBV (MOI)	0	50	50	50	50	50
ATRA (μM)	0	0	1	1	1	1
SC shRNA (μg)	1.0	1.0	1.0	0	0	0
E6AP shRNA (μg)	0	0	0	1.0	0	0
E6AP (μg)	0	0	0	0	0	1.0

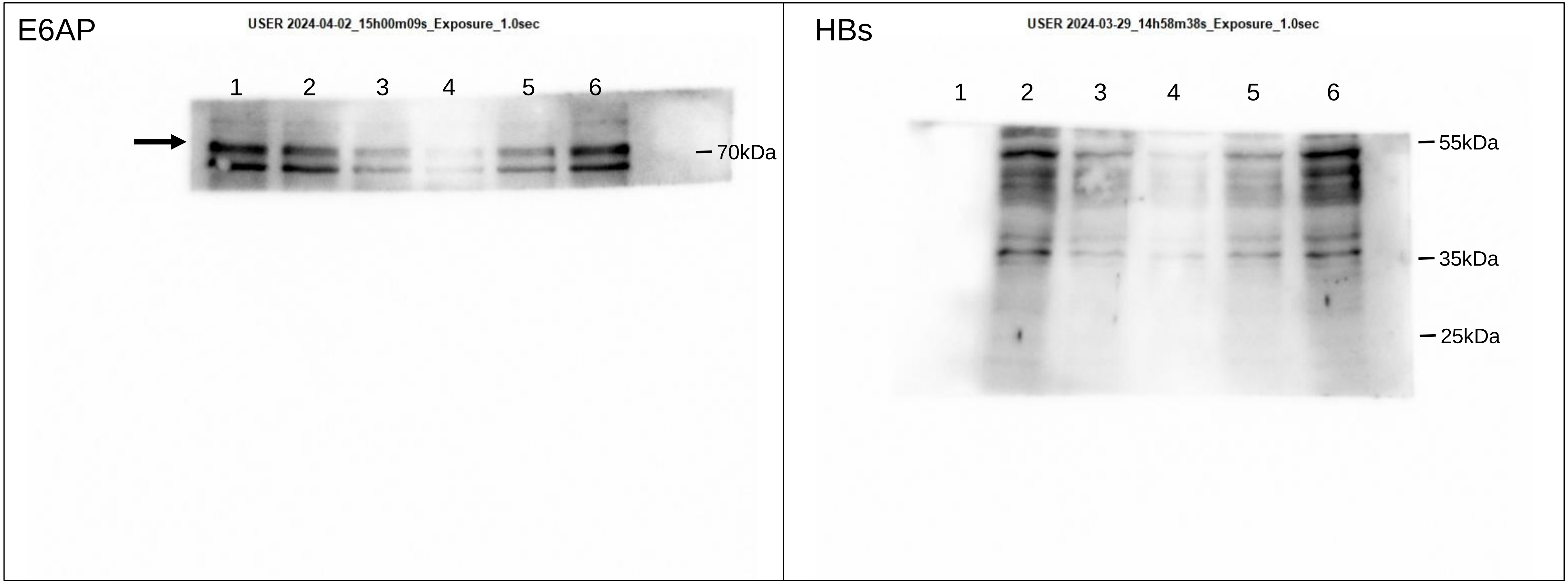

Fig. 6e

Lanes	1	2	3	4	5	6
HBV (MOI)	0	50	50	50	50	50
ATRA (μM)	0	0	1	1	1	1
SC shRNA (μg)	1.0	1.0	1.0	0	0	0
E6AP shRNA (μg)	0	0	0	1.0	0	0
E6AP (μg)	0	0	0	0	0	1.0

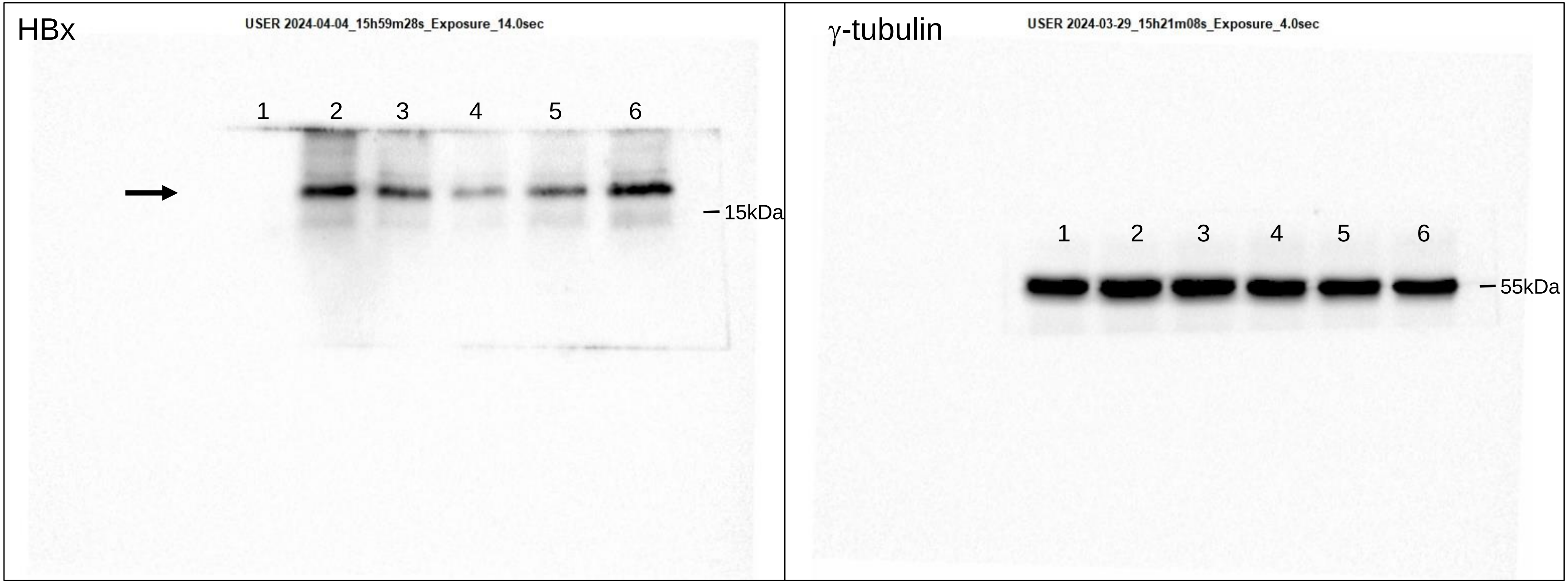

Fig. 6g

HBV (MOI)	50	50	50	50
ATRA (μM)	0	5	5	5
SC shRNA (μg)	1	1	0.9	0
E6AP shRNA (μg)	0	0	0.1	1.0

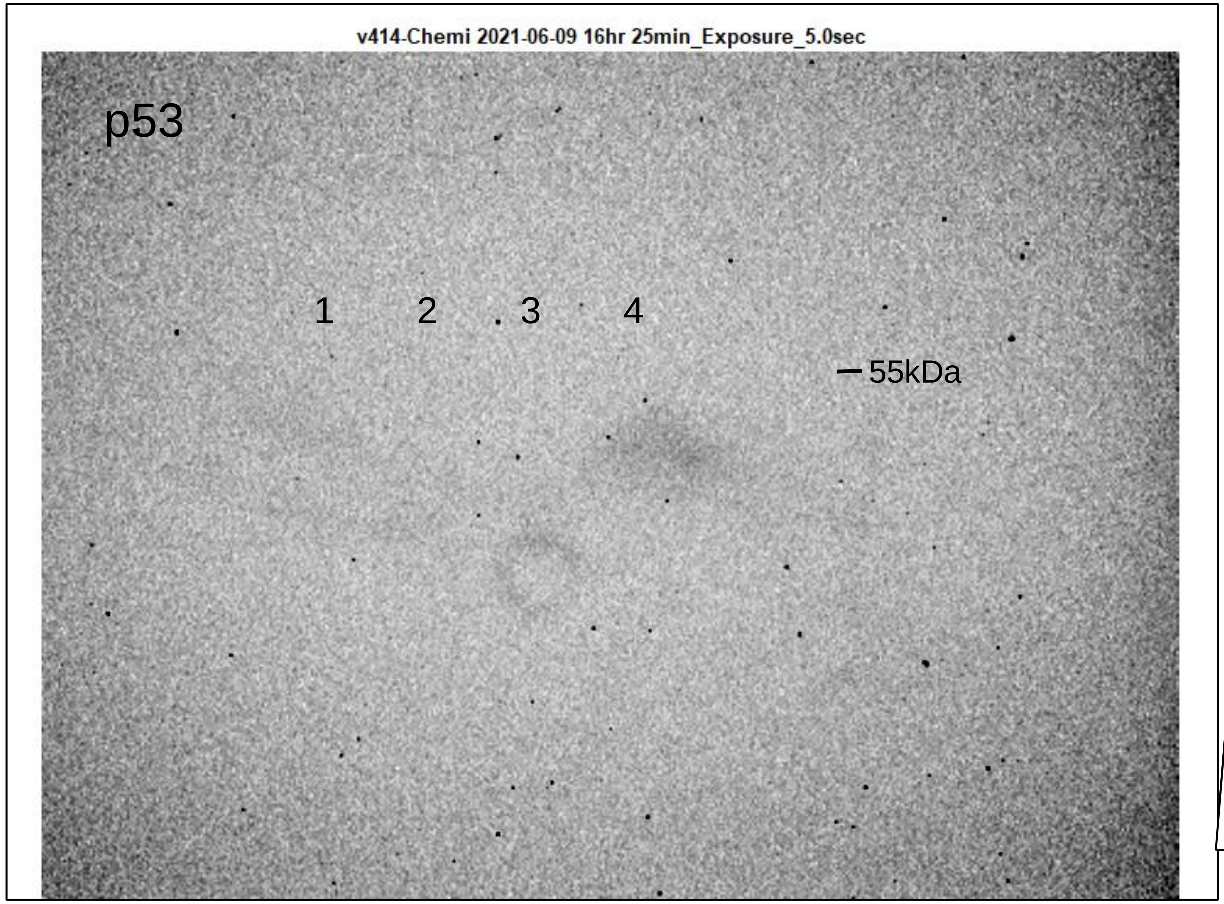

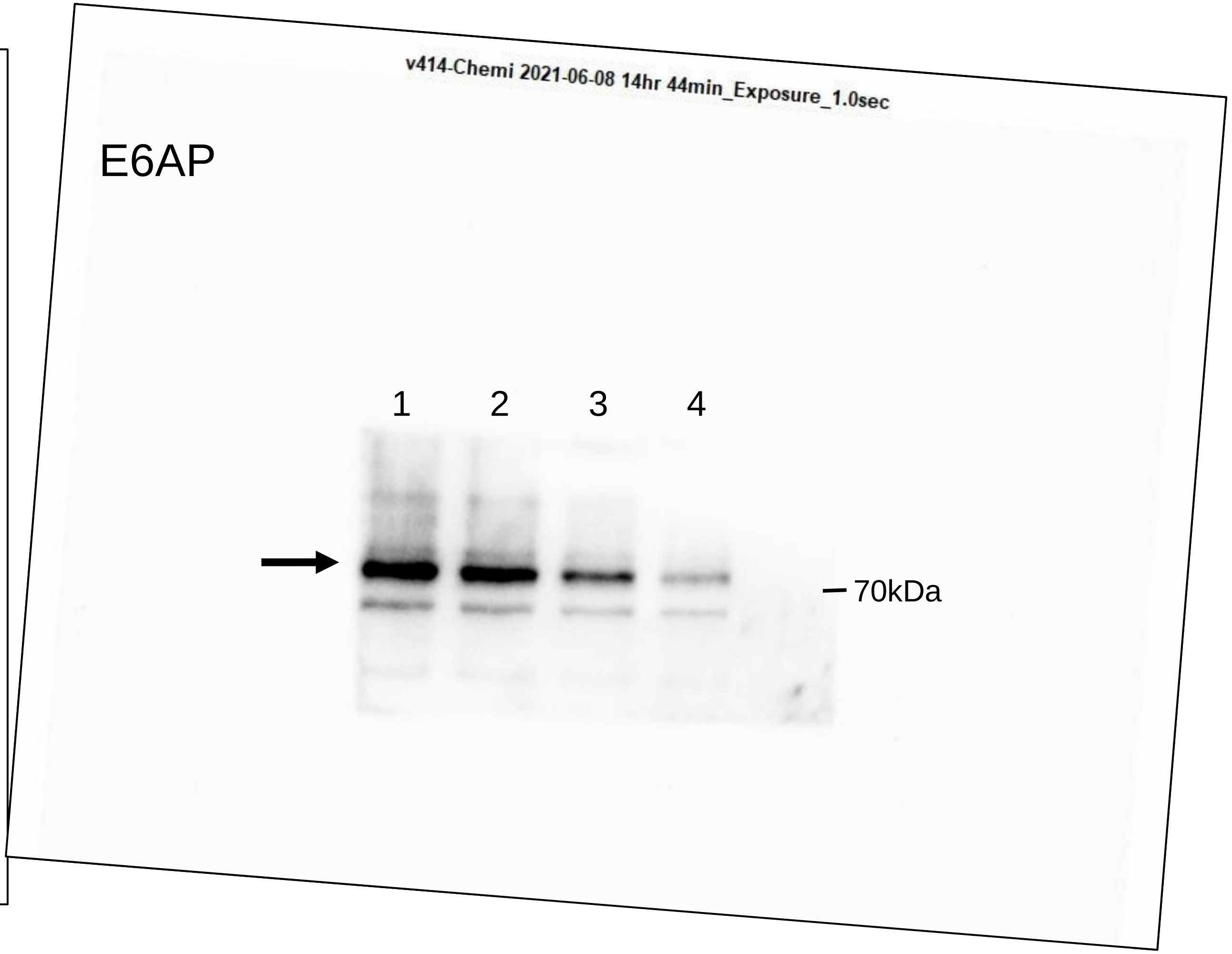

Fig. 6g

HBV (MOI)	50	50	50	50
ATRA (μM)	0	5	5	5
SC shRNA (μg)	1	1	0.9	0
E6AP shRNA (μg)	0	0	0.1	1.0

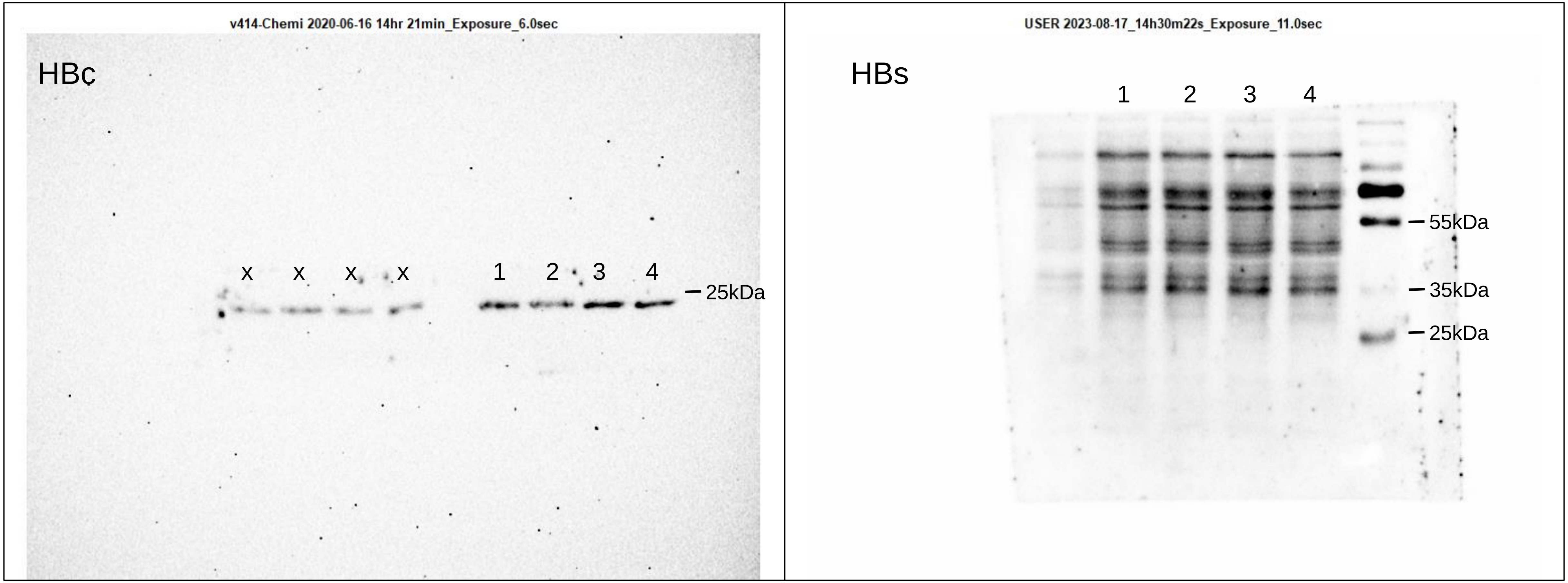

Fig. 6g

HBV (MOI)	50	50	50	50
ATRA (μM)	0	5	5	5
SC shRNA (μg)	1	1	0.9	0
E6AP shRNA (μg)	0	0	0.1	1.0

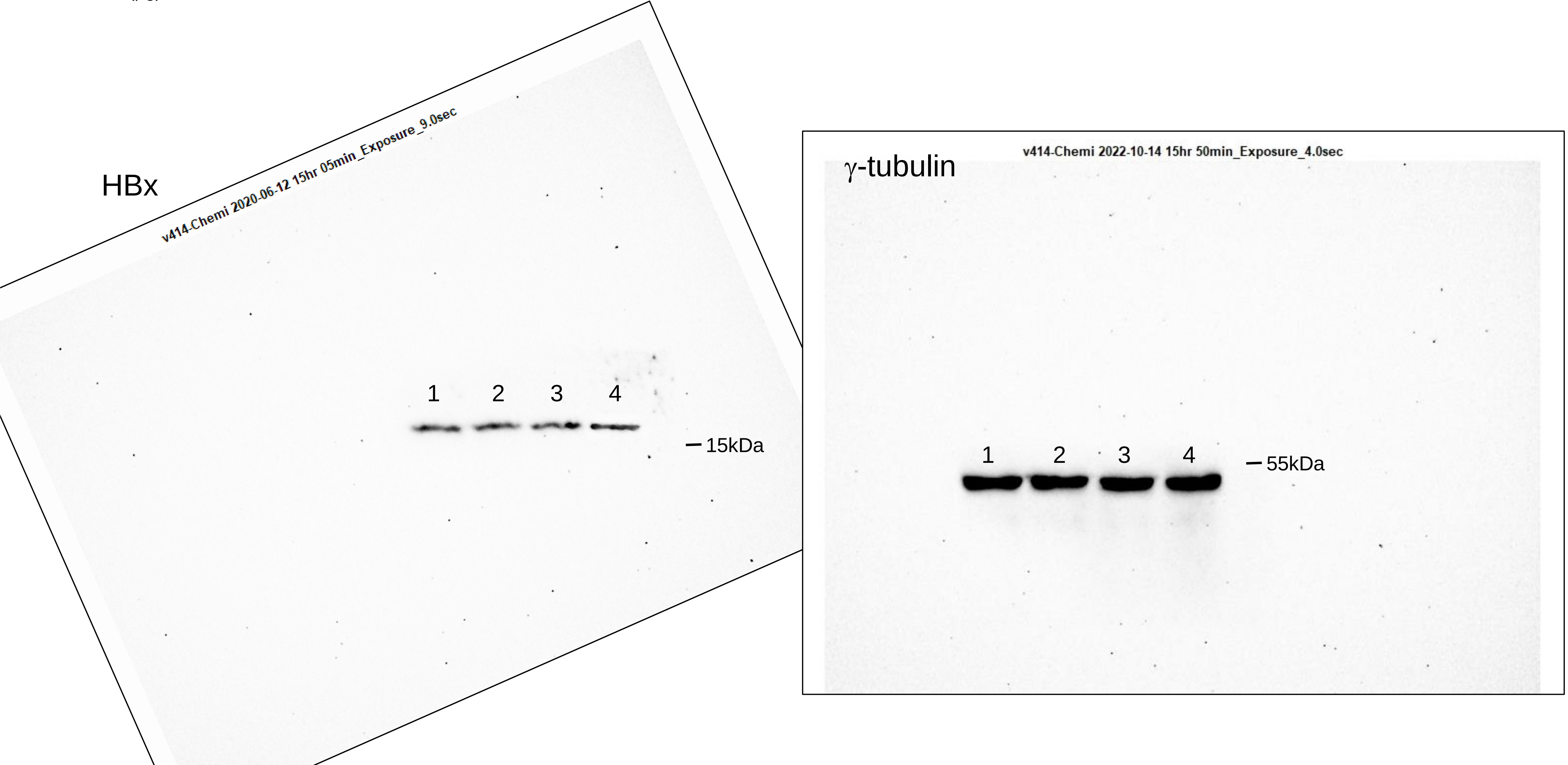

Supplement: S1 Raw image — (PDF) [file pone.0305350.s001.pdf]
