# Supplementary material for: All-trans retinoic acid downregulates HBx levels via E6-associated protein-mediated proteasomal degradation to suppress hepatitis B virus replication
Source: PLoS One. 2024 Jun 11;19(6):e0305350. doi: 10.1371/journal.pone.0305350 (PMC11166335; doi:10.1371/journal.pone.0305350)
Supplement: Attachment — Submitted filename: Major and minor revision.docx [file pone.0305350.s002.docx]

Authors have presented an interesting study in the manuscript entitled “All-trans retinoic acid downregulates HBx levels via E6-associated protein-mediated proteasomal degradation to suppress hepatitis B virus replication” where they have tried to solve the complex riddle of cross talk between HBx, P53, ATRA and E6AP. However, there are few concerns that need to be addressed.

Comments:

1. Result 2 shows that expression of p53, Siah-1 and E6AP varies depending upon the concentration of ATRA used in the experiment. Why 5mM concentration was selected and not the lower concentration for the study from fig 3 onwards.

2. HBx protein has been shown to be required for successful HBV infection and replication. In the absence of HBx, only basal level HBV replication is expected. In result 2 (a), authors have shown that ATRA failed to diminish the HBV protein expression in HepG2 cells infected with HBx null HBV and have concluded that HBx is required for inhibitory effect of ATRA. It would be better to include the same experimental data in Hep3B cells (Effect of ATRA on HBV proteins in Hep3B cells infected with HBx null HBV) to make it more relatable for the readers.

3. In result 3, is there an explanation for ATRA downregulating E6AP levels at low concentrations and upregulating at higher concentrations. What is the reason for its reverse effect (ATRA increases p53 and Siah-1 level at low concentration and decreases at higher concentration) on expression of p53 and Siah-1 levels.

4. Result 5 title does not match with the results explained. The data and the explanation say that ATRA “increases” HBx degradation via E6AP mediated ubiquitination and thus suppresses HBV replication. However, the title of the figure says that ATRA “downregulates” p53-dependent HBx degradation via E6AP ubiquitination to suppress HBV replication.

5. In result 5, the data has been produced at 5mM ATRA concentration. It would be interesting to know that how does lower ATRA concentration affects E6AP mediated ubiquitination and HBx degradation? If results vary compared to effects shown at 5mM ATRA concentration?

6. HepG2.2.15 cells are stably HBV expressing lines and were designed to study the HBV infection, replication, and life cycle. It would be advisable to replicate the key findings from this paper (ATRA treatment increases E6AP which in turn ubiquitinates HBx to suppress HBV replication) in this cell line.

7. Figure legend 2(a), It says “HepG2-NRCP” rather than “HepG2-NTCP”. Please correct that.
